# Supplementary material for: Catalytic Enantioselective Synthesis of N-C Axially Chiral N-(2,6-Disubstituted-phenyl)sulfonamides through Chiral Pd-Catalyzed N-Allylation
Source: Molecules. 2022 Nov 13;27(22):7819. doi: 10.3390/molecules27227819 (PMC9698006; doi:10.3390/molecules27227819)
Supplement: Supplementary file 1 [file molecules-27-07819-s001.zip › molecules-1999254-supplementary.pdf]

Supplementary Materials

Catalytic Enantioselective Synthesis of N-C Axially Chiral Sulfonamides Bearing ortho-disubstituted-Phenyl Groups

Sota Fukasawa,[1] Tatsuya Toyoda,[1] Ryohei Kasahara,[1] Chisato Nakamura,[1] Yuuki Kikuchi,[1] Akiko Hori,[2] Gary J. Richards[2] and Osamu Kitagawa*[1]

[1]Department of Applied Chemistry (Japanese Association of Bio-intelligence for Well-being), Shibaura Institute of Technology, 3-7-5 Toyosu, Kohto-ku, Tokyo 135-8548, Japan.

[2]Graduate School of Engineering and Science, Shibaura Institute of Technology, 307 Fukasaku, Minuma-ku, Saitama 337-8570, Japan.

*****Correspondence: kitagawa@shibaura-it.ac.jp; Tel.: +81-3-5859-8161 (O.K.)

(Contents)

Copies of ^{1}H-and^{13}C-NMR chart of all compounds **1-6** S2-S44

Chiral HPLC chart for the determination of the ee S45-S64

Xray crystal data of **2o** S65-S68

Kinetic data for the evaluation of the rotational barriers of **2i** and **2r** S69

Copies of ^{1}H- and ^{13}C-NMR spectra

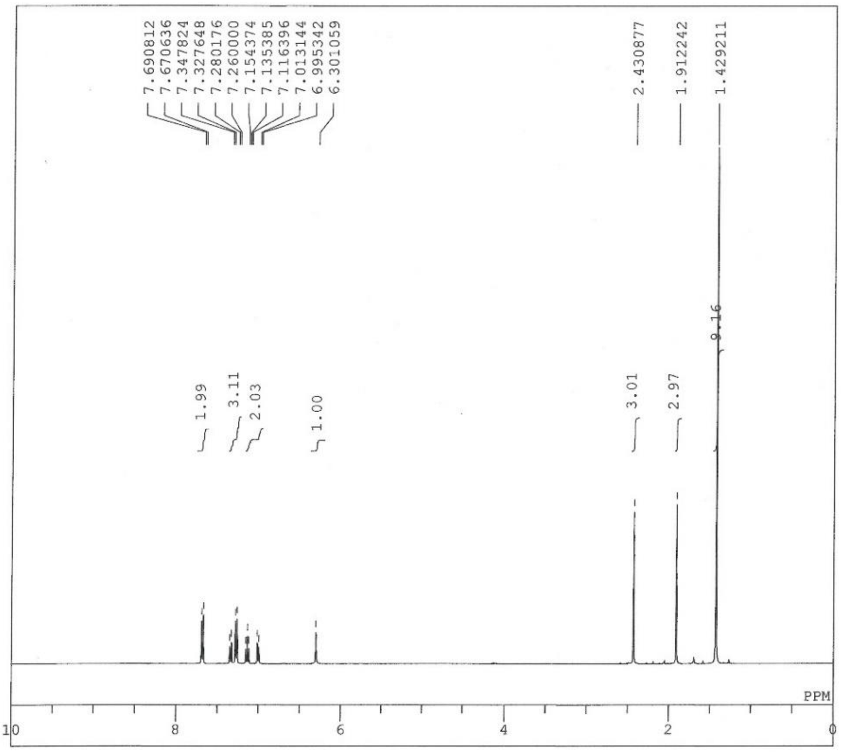

DFILE SF131 20210916-1.als
COMNT Qn-BOC 1H
DATIM 2021-09-16 14:24:05
OBNUC 1H
EXMOD single_pulse.ex2
OBFRQ 399.78 MHz
OBSET 4.62 KHz
OBFIN 5.98 Hz
POINT 13107
FREQU 6218.81 Hz
SCANS 32
ACQTM 2.1076 sec
PD 5.0000 sec
PW1 5.17 usec
IRNUC 1H
CTEMP 18.8 c
SLVNT CDCL3
EXREF 7.26 ppm
BF 0.12 Hz
RGAIN 30

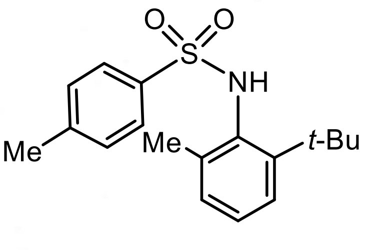

1a

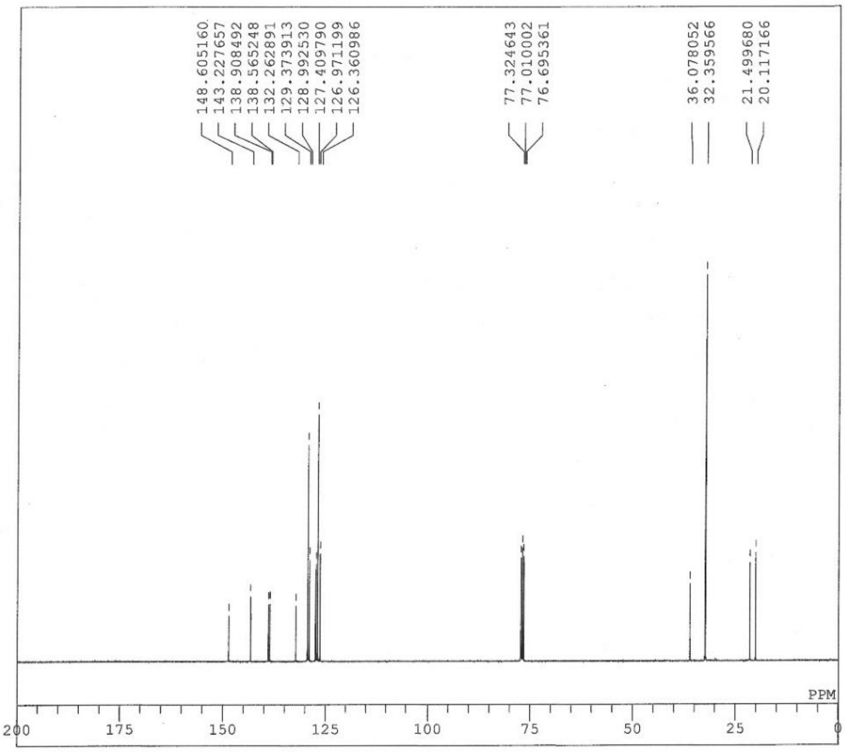

DFILE SF131-13C 20211008-1.als
COMNT
DATIM 2021-10-08 09:19:14
OBNUC 13C
EXMOD single_pulse_dec
OBFRQ 100.53 MHz
OBSET 5.35 KHz
OBFIN 5.86 Hz
POINT 32768
FREQU 31407.03 Hz
SCANS 1024
ACQTM 1.0433 sec
PD 2.0000 sec
PW1 3.03 usec
IRNUC 1H
CTEMP 18.7 c
SLVNT CDCL3
EXREF 77.01 ppm
BF 1.20 Hz
RGAIN 60

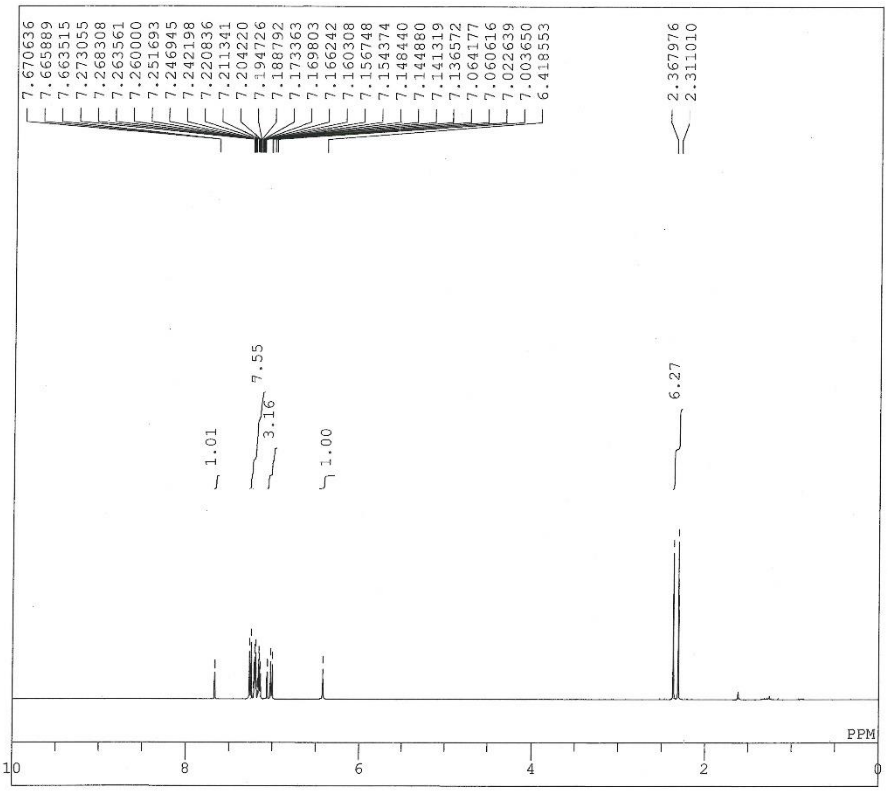

DFILE RK031 20211214-1.als
COMNT Qn-BOC 1H
DATIM 2021-12-14 11:23:11
OBNUC 1H
EXMOD single_pulse.ex2
OBFRQ 399.78 MHz
OBSET 4.62 KHz
OBFIN 5.98 Hz
POINT 16384
FREQU 7773.63 Hz
SCANS 32
ACQTM 2.1076 sec
PD 5.0000 sec
PW1 5.80 usec
IRNUC 1H
CTEMP 17.5 c
SLVNT CDCL3
EXREF 7.26 ppm
BF 0.12 Hz
RGAIN 30

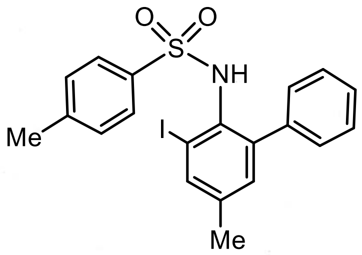

1b

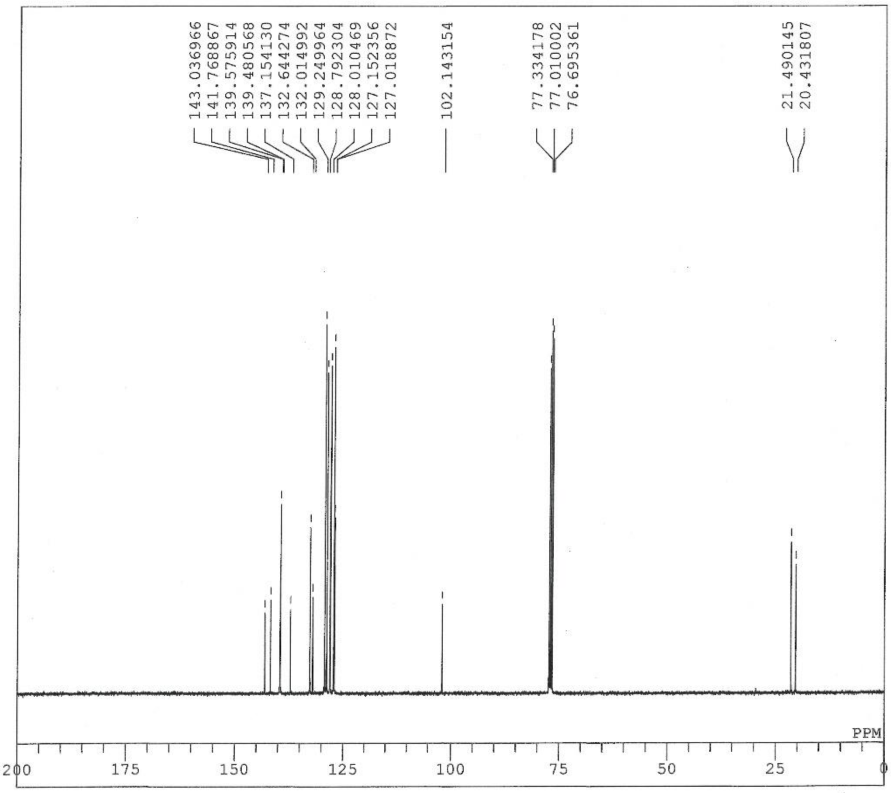

DFILE SF068-13C 20211005-1.als
COMNT
DATIM 2021-10-05 09:40:09
OBNUC 13C
EXMOD single_pulse_dec
OBFRQ 100.53 MHz
OBSET 5.35 KHz
OBFIN 5.86 Hz
POINT 32768
FREQU 31407.03 Hz
SCANS 1024
ACQTM 1.0433 sec
PD 2.0000 sec
PW1 3.03 usec
IRNUC 1H
CTEMP 19.0 c
SLVNT CDCL3
EXREF 77.01 ppm
BF 1.20 Hz
RGAIN 60

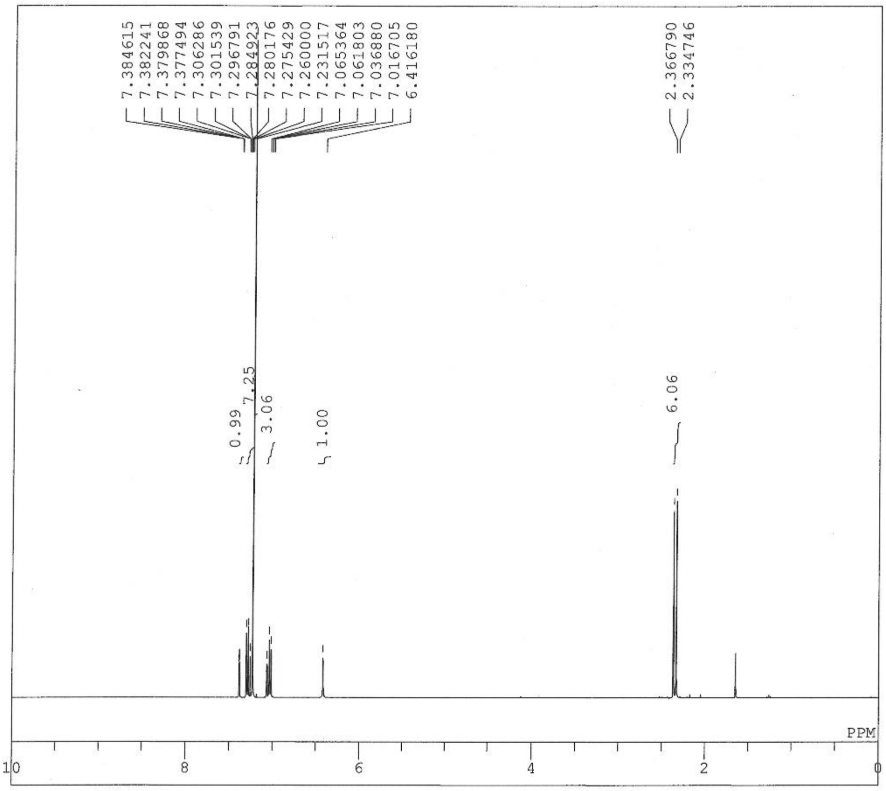

DFILE SF133 20211012-1.als
COMNT Qn-BOC 1H
DATIM 2021-10-12 12:33:47
OBNUC 1H
EXMOD single_pulse.ex2
OBFRQ 399.78 MHz
OBSET 4.62 KHz
OBFIN 5.98 Hz
POINT 13107
FREQU 6218.81 Hz
SCANS 32
ACQTM 2.1076 sec
PD 5.0000 sec
PW1 5.17 usec
IRNUC 1H
CTEMP 18.7 c
SLVNT CDCL3
EXREF 7.26 ppm
BF 0.12 Hz
RGAIN 34

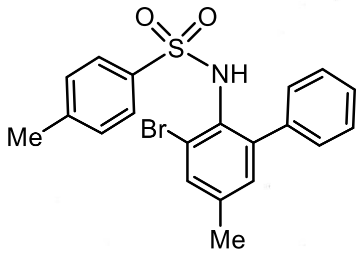

1c

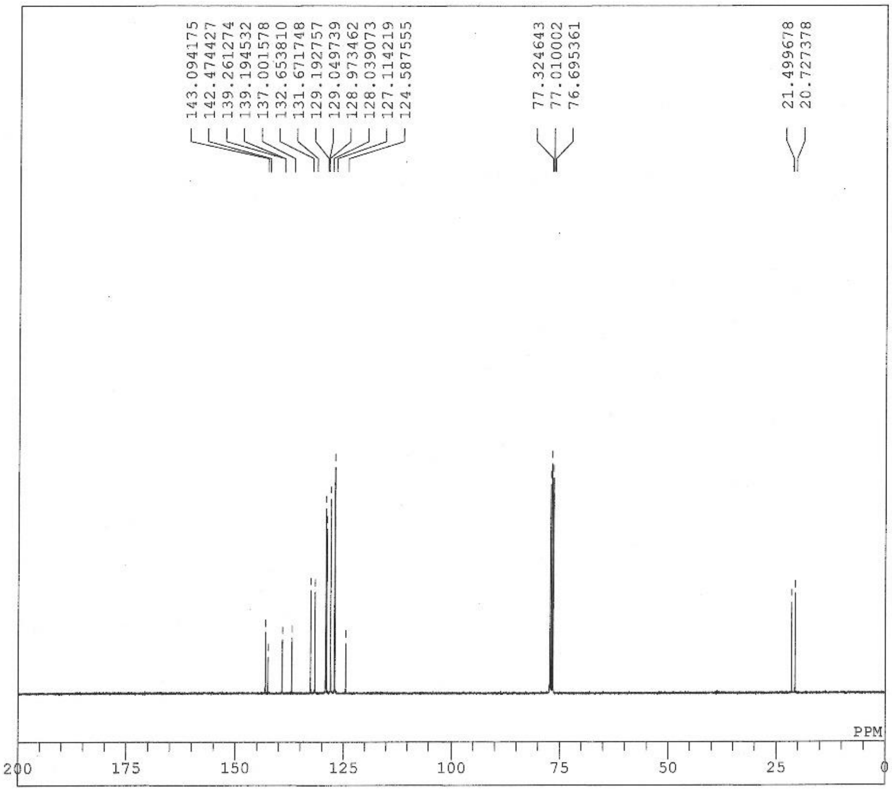

DFILE SF133-13C 20211011-1.als
COMNT
DATIM 2021-10-11 17:31:11
OBNUC 13C
EXMOD single_pulse_dec
OBFRQ 100.53 MHz
OBSET 5.35 KHz
OBFIN 5.86 Hz
POINT 32768
FREQU 31407.03 Hz
SCANS 1024
ACQTM 1.0433 sec
PD 2.0000 sec
PW1 3.03 usec
IRNUC 1H
CTEMP 19.0 c
SLVNT CDCL3
EXREF 77.01 ppm
BF 1.20 Hz
RGAIN 60

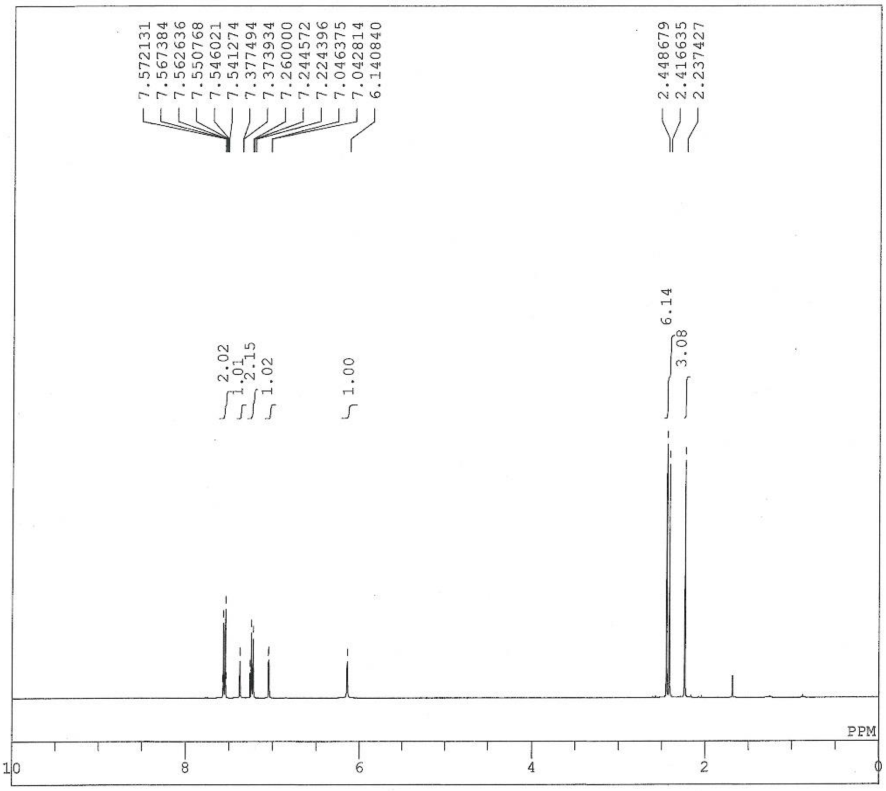

DFILE TT026 20211008-1.als
COMNT Qn-BOC 1H
DATIM 2021-10-08 13:47:14
OBNUC 1H
EXMOD single_pulse.ex2
OBFRQ 399.78 MHz
OBSET 4.62 KHz
OBFIN 5.98 Hz
POINT 16384
FREQU 7773.63 Hz
SCANS 32
ACQTM 2.1076 sec
PD 5.0000 sec
PW1 5.17 usec
IRNUC 1H
CTEMP 18.6 c
SLVNT CDCL3
EXREF 7.26 ppm
BF 0.12 Hz
RGAIN 30

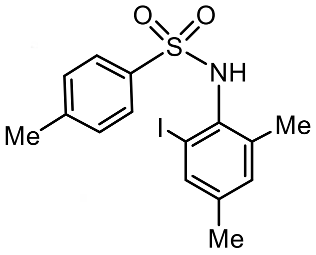

1d

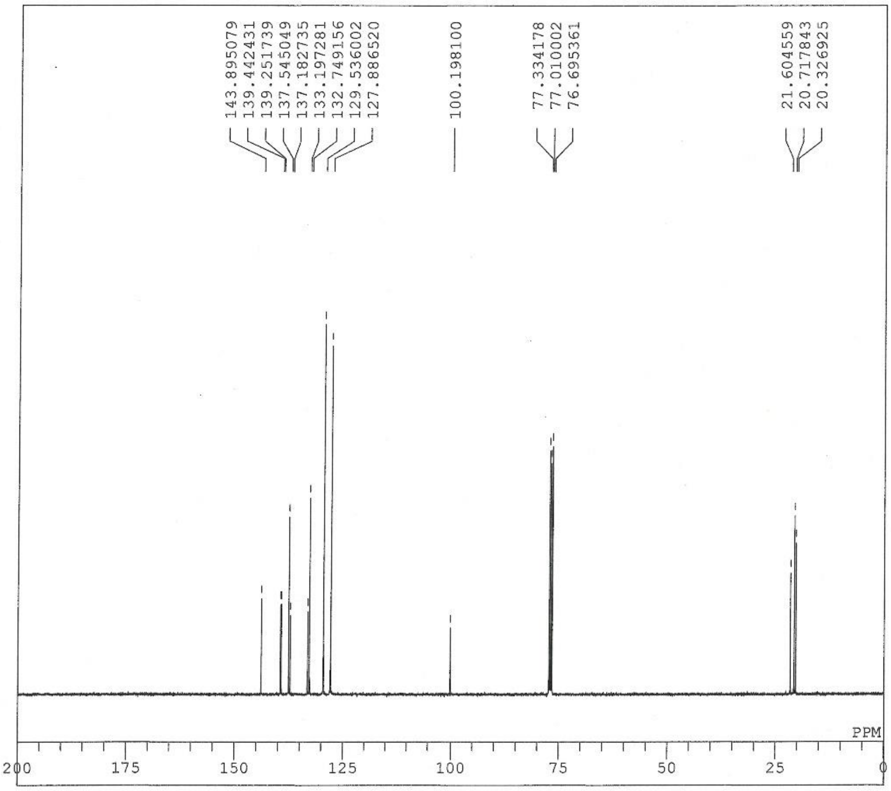

DFILE TT026-13C 20211007-1.als
COMNT
DATIM 2021-10-07 18:36:27
OBNUC 13C
EXMOD single_pulse_dec
OBFRQ 100.53 MHz
OBSET 5.35 KHz
OBFIN 5.86 Hz
POINT 32768
FREQU 31407.03 Hz
SCANS 1024
ACQTM 1.0433 sec
PD 2.0000 sec
PW1 3.03 usec
IRNUC 1H
CTEMP 18.7 c
SLVNT CDCL3
EXREF 77.01 ppm
BF 1.20 Hz
RGAIN 60

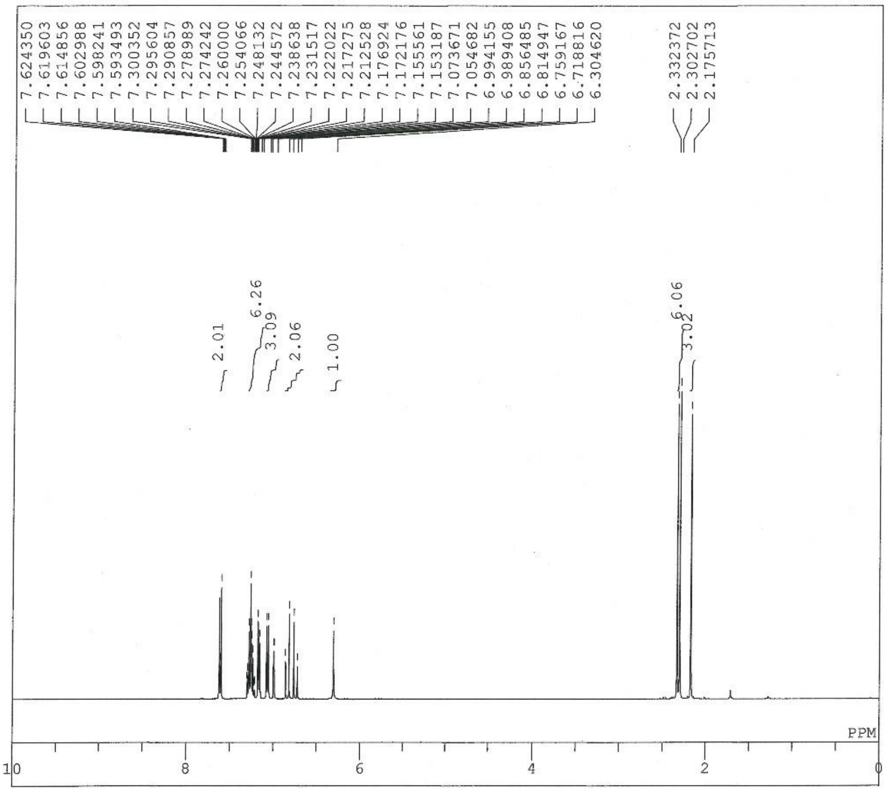

DFILE TT041 20211014-1.als
COMNT Qn-BOC 1H
DATIM 2021-10-14 15:38:48
OBNUC 1H
EXMOD single_pulse.ex2
OBFRQ 399.78 MHz
OBSET 4.62 KHz
OBFIN 5.98 Hz
POINT 13107
FREQU 6218.81 Hz
SCANS 32
ACQTM 2.1076 sec
PD 5.0000 sec
PW1 5.17 usec
IRNUC 1H
CTEMP 18.3 c
SLVNT CDCL3
EXREF 7.26 ppm
BF 0.12 Hz
RGAIN 30

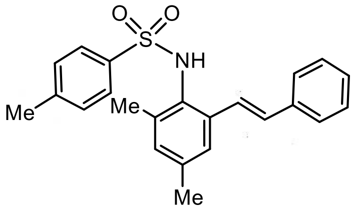

1e

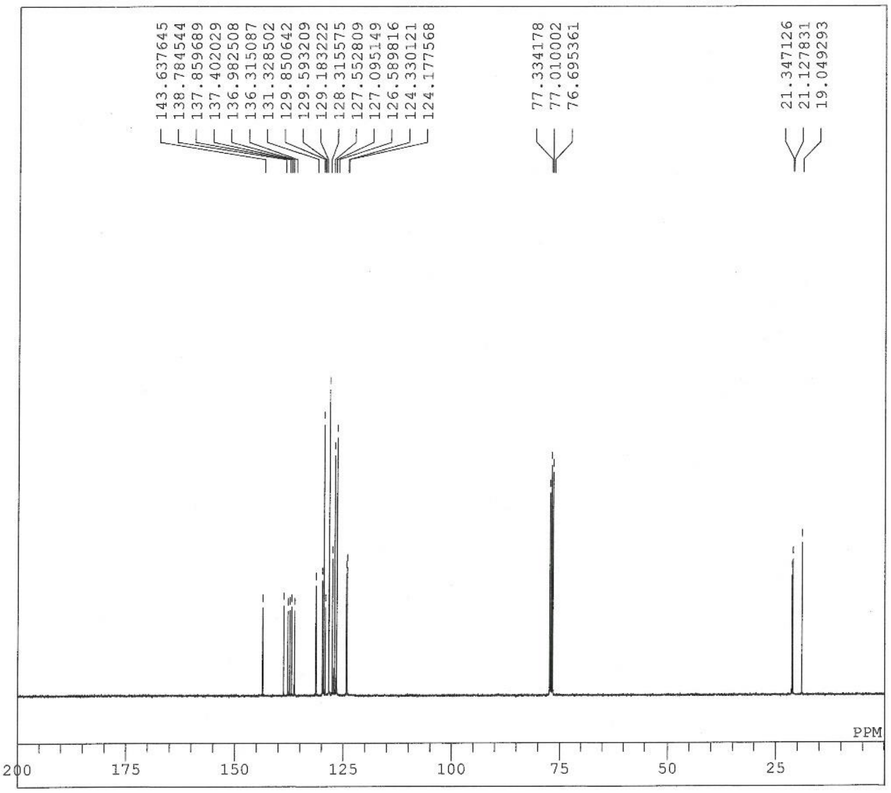

DFILE TT041-13C 20211014-1.als
COMNT
DATIM 2021-10-14 18:10:29
OBNUC 13C
EXMOD single_pulse_dec
OBFRQ 100.53 MHz
OBSET 5.35 KHz
OBFIN 5.86 Hz
POINT 32768
FREQU 31407.03 Hz
SCANS 1024
ACQTM 1.0433 sec
PD 2.0000 sec
PW1 3.03 usec
IRNUC 1H
CTEMP 18.7 c
SLVNT CDCL3
EXREF 77.01 ppm
BF 1.20 Hz
RGAIN 60

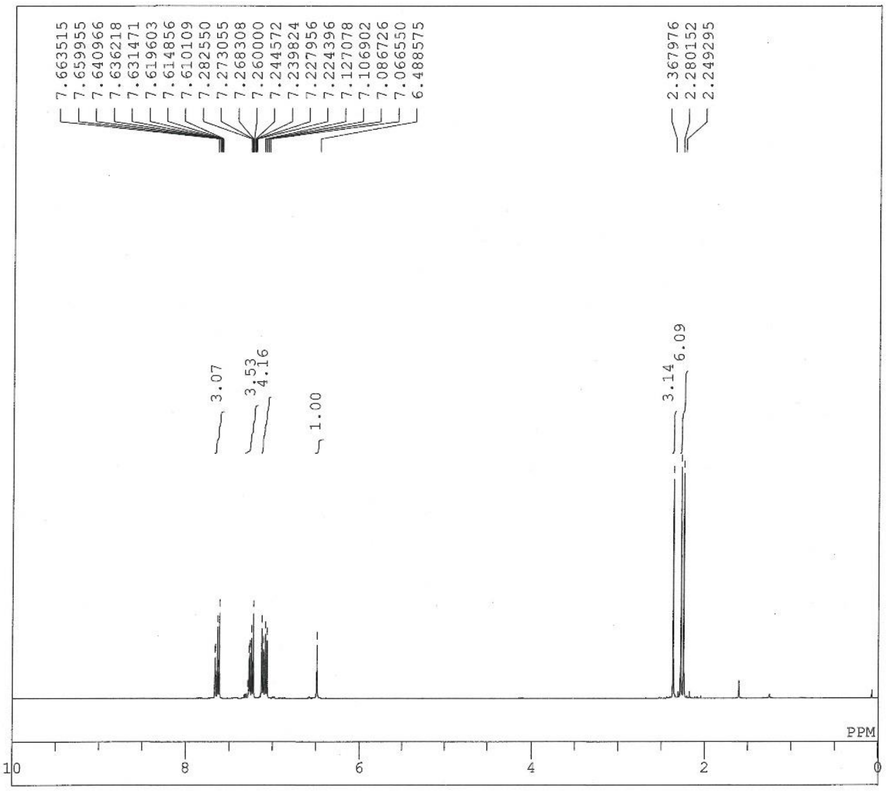

DFILE SF137 20211014-1.als
COMNT Qn-BOC 1H
DATIM 2021-10-14 16:00:16
OBNUC 1H
EXMOD single_pulse.ex2
OBFRQ 399.78 MHz
OBSET 4.62 KHz
OBFIN 5.98 Hz
POINT 13107
FREQU 6218.81 Hz
SCANS 32
ACQTM 2.1076 sec
PD 5.0000 sec
PW1 5.17 usec
IRNUC 1H
CTEMP 18.3 c
SLVNT CDCL3
EXREF 7.26 ppm
BF 0.12 Hz
RGAIN 38

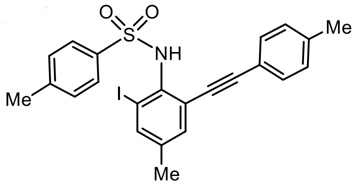

1f

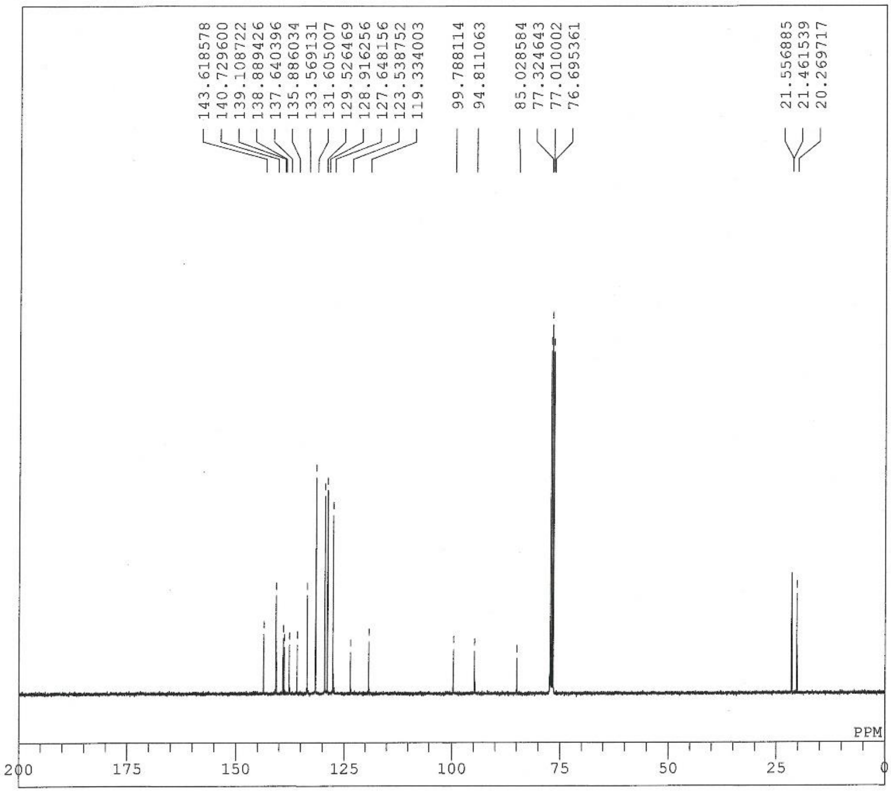

DFILE SF137-13C 20211015-1.als
COMNT
DATIM 2021-10-15 17:31:14
OBNUC 13C
EXMOD single_pulse_dec
OBFRQ 100.53 MHz
OBSET 5.35 KHz
OBFIN 5.86 Hz
POINT 32768
FREQU 31407.03 Hz
SCANS 1024
ACQTM 1.0433 sec
PD 2.0000 sec
PW1 3.03 usec
IRNUC 1H
CTEMP 18.7 c
SLVNT CDCL3
EXREF 77.01 ppm
BF 1.20 Hz
RGAIN 60

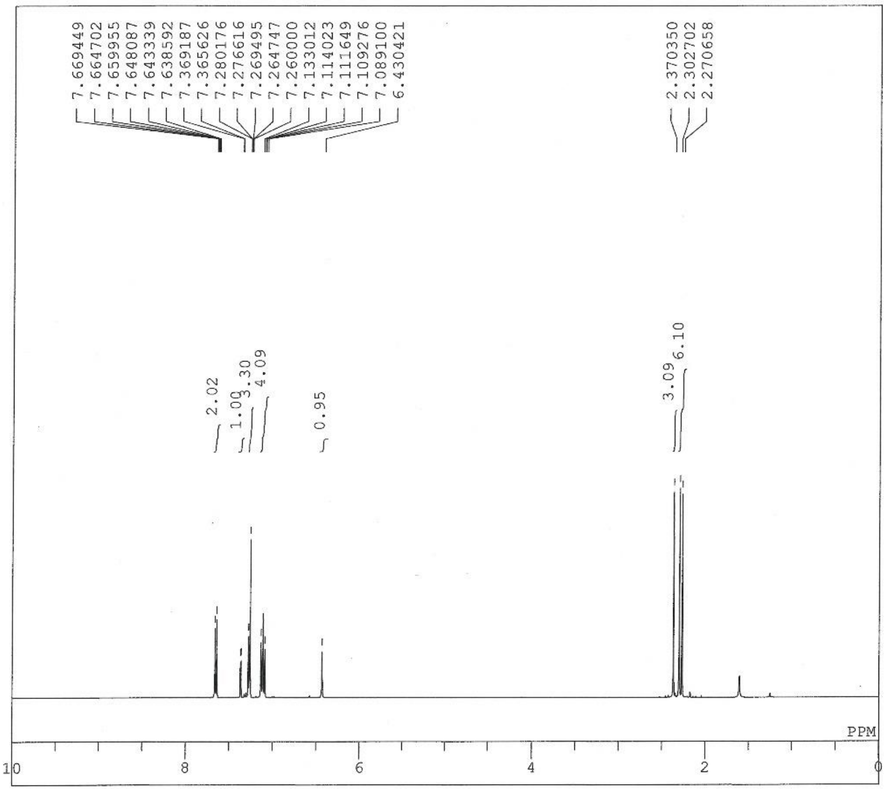

DFILE YK308 20210916-1.als
COMNT Qn-BOC 1H
DATIM 2021-09-16 15:31:22
OBNUC 1H
EXMOD single_pulse.ex2
OBFRQ 399.78 MHz
OBSET 4.62 KHz
OBFIN 5.98 Hz
POINT 16384
FREQU 7773.63 Hz
SCANS 32
ACQTM 2.1076 sec
PD 5.0000 sec
PW1 5.17 usec
IRNUC 1H
CTEMP 19.2 c
SLVNT CDCL3
EXREF 7.26 ppm
BF 0.12 Hz
RGAIN 38

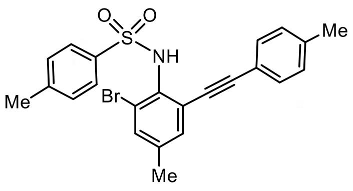

1g

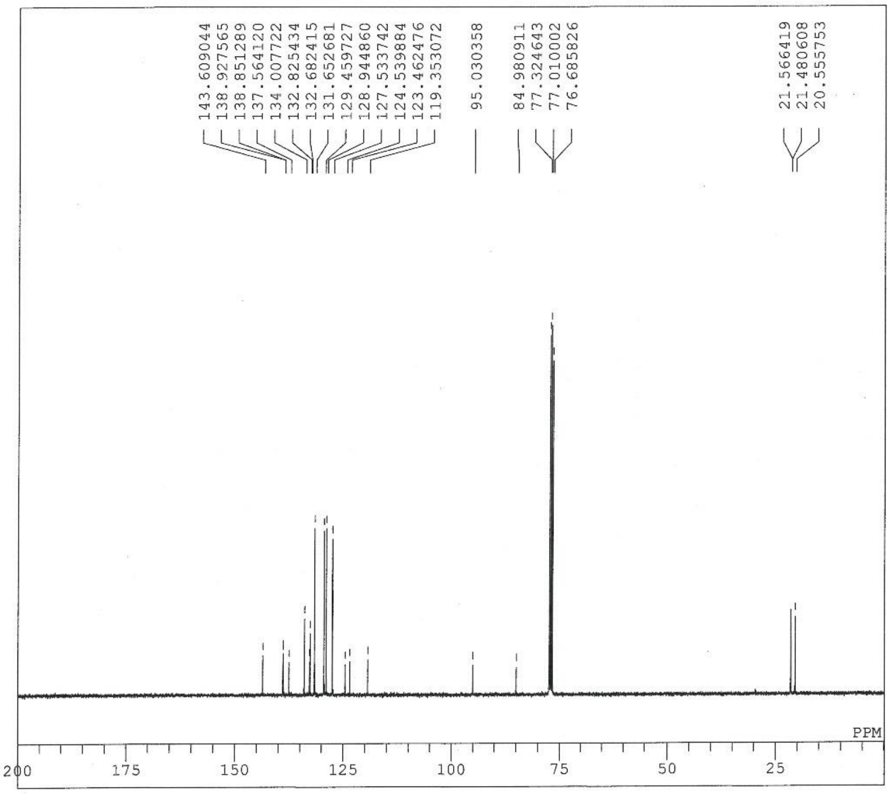

DFILE YK308-13C 20210915-1.als
COMNT
DATIM 2021-09-15 09:29:30
OBNUC 13C
EXMOD single_pulse_dec
OBFRQ 100.53 MHz
OBSET 5.35 KHz
OBFIN 5.86 Hz
POINT 32768
FREQU 31407.03 Hz
SCANS 1024
ACQTM 1.0433 sec
PD 2.0000 sec
PW1 3.03 usec
IRNUC 1H
CTEMP 19.1 c
SLVNT CDCL3
EXREF 77.01 ppm
BF 1.20 Hz
RGAIN 60

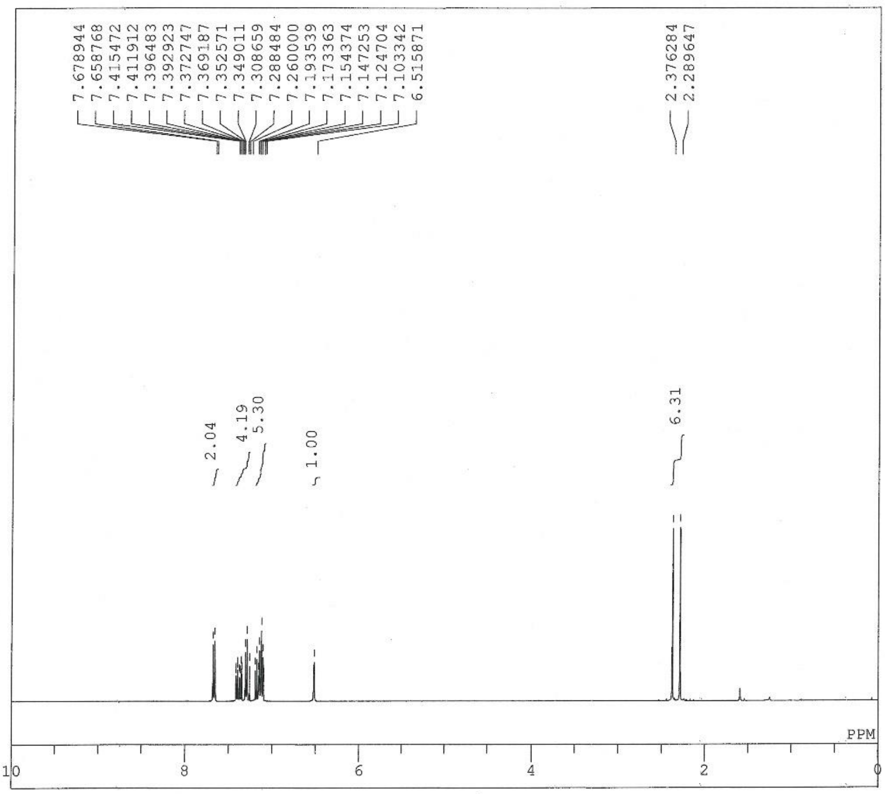

DFILE SF107 20211121-1.als
COMNT Qn-BOC 1H
DATIM 2021-11-21 17:28:03
OBNUC 1H
EXMOD single_pulse.ex2
OBFRQ 399.78 MHz
OBSET 4.62 KHz
OBFIN 5.98 Hz
POINT 16384
FREQU 7773.63 Hz
SCANS 32
ACQTM 2.1076 sec
PD 5.0000 sec
PW1 5.80 usec
IRNUC 1H
CTEMP 18.4 c
SLVNT CDCL3
EXREF 7.26 ppm
BF 0.12 Hz
RGAIN 34

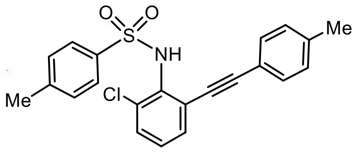

1h

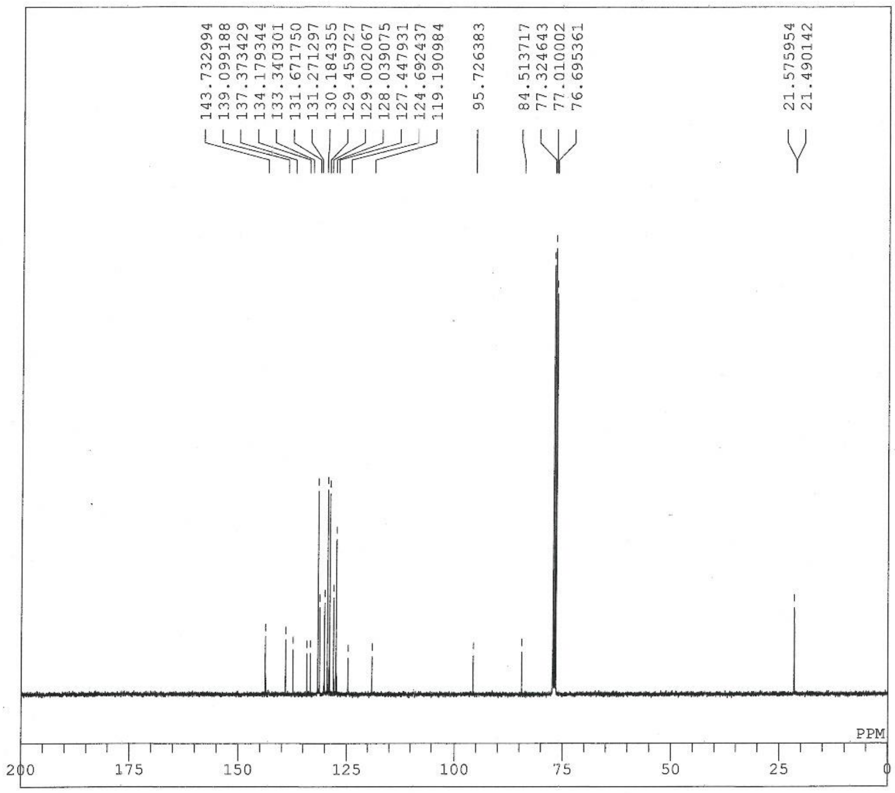

DFILE SF107-13C 20211121-1.als
COMNT
DATIM 2021-11-21 15:25:46
OBNUC 13C
EXMOD single_pulse_dec
OBFRQ 100.53 MHz
OBSET 5.35 KHz
OBFIN 5.86 Hz
POINT 32768
FREQU 31407.03 Hz
SCANS 1024
ACQTM 1.0433 sec
PD 2.0000 sec
PW1 3.23 usec
IRNUC 1H
CTEMP 18.6 c
SLVNT CDCL3
EXREF 77.01 ppm
BF 1.20 Hz
RGAIN 60

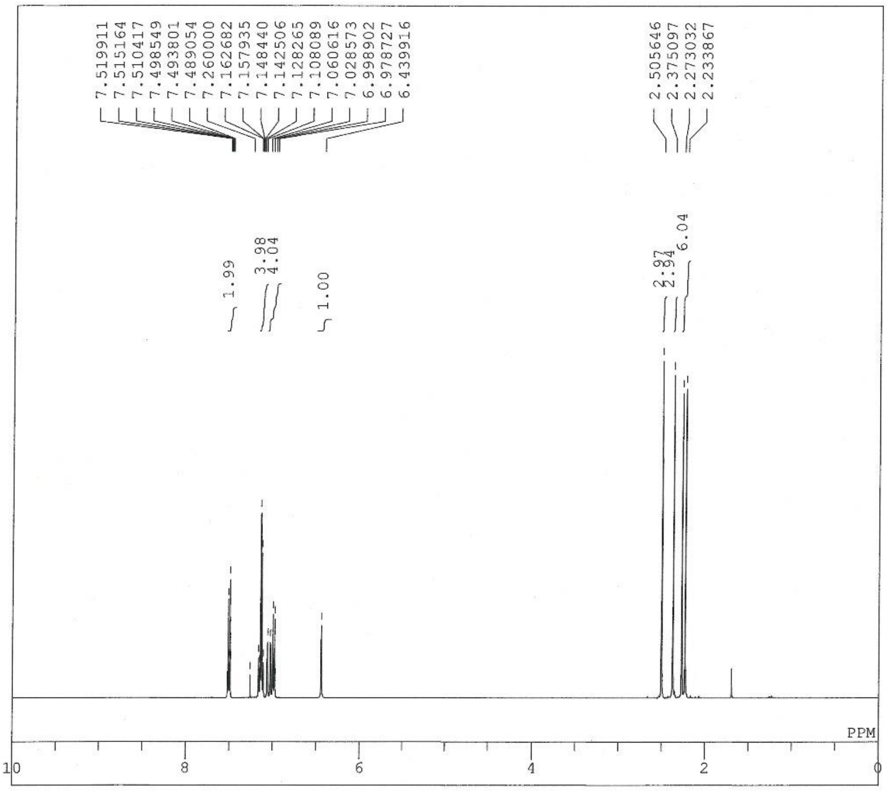

DFILE TT045 20211005-1.als
COMNT Qn-BOC 1H
DATIM 2021-10-05 14:50:54
OBNUC 1H
EXMOD single_pulse.ex2
OBFRQ 399.78 MHz
OBSET 4.62 KHz
OBFIN 5.98 Hz
POINT 13107
FREQU 6218.81 Hz
SCANS 32
ACQTM 2.1076 sec
PD 5.0000 sec
PW1 5.17 usec
IRNUC 1H
CTEMP 18.8 c
SLVNT CDCL3
EXREF 7.26 ppm
BF 0.12 Hz
RGAIN 30

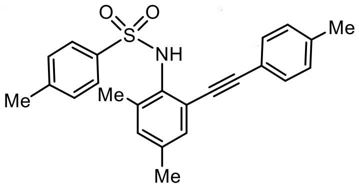

1i

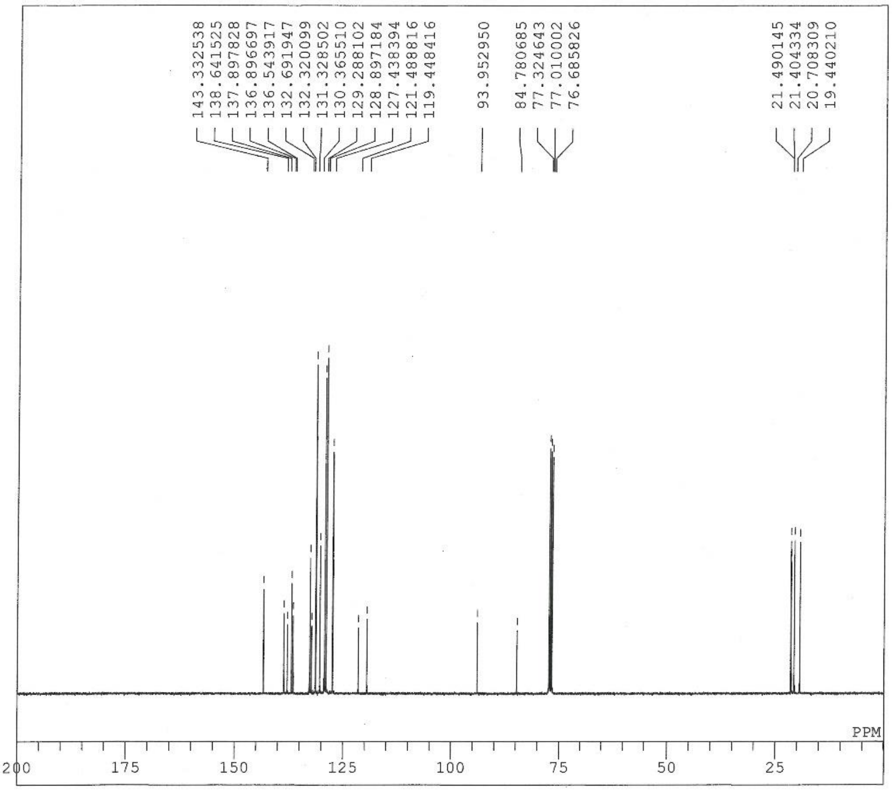

DFILE TT045-13C 20211002-1.als
COMNT
DATIM 2021-10-02 15:12:02
OBNUC 13C
EXMOD single_pulse_dec
OBFRQ 100.53 MHz
OBSET 5.35 KHz
OBFIN 5.86 Hz
POINT 32768
FREQU 31407.03 Hz
SCANS 1024
ACQTM 1.0433 sec
PD 2.0000 sec
PW1 3.03 usec
IRNUC 1H
CTEMP 19.0 c
SLVNT CDCL3
EXREF 77.01 ppm
BF 1.20 Hz
RGAIN 60

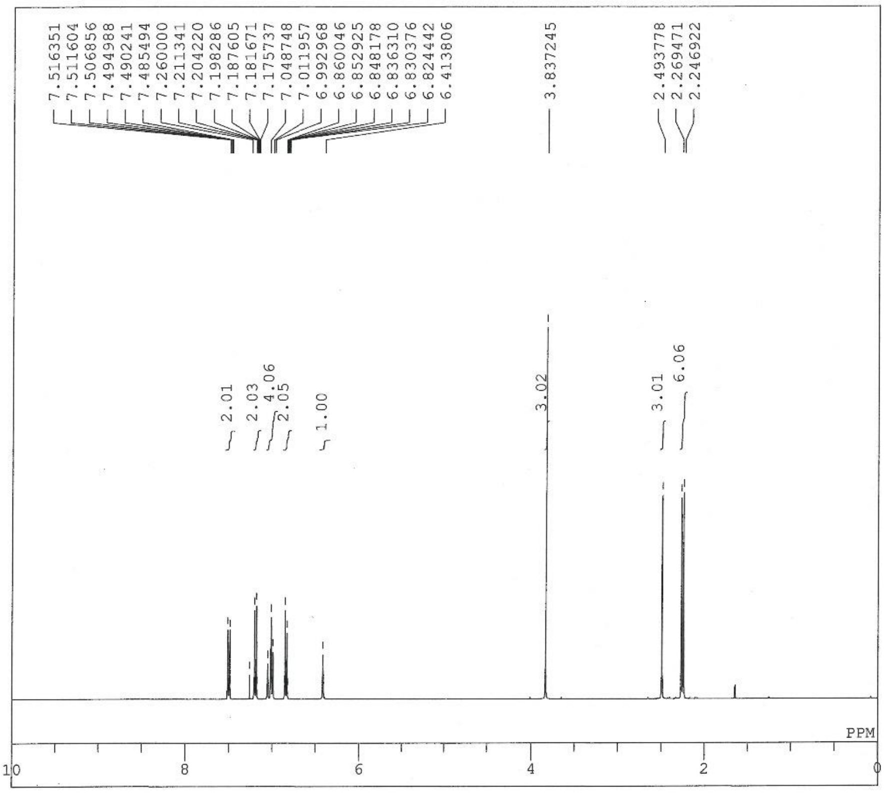

DFILE SF105 20210928-1.als
COMNT Qn-BOC 1H
DATIM 2021-09-28 16:19:29
OBNUC 1H
EXMOD single_pulse.ex2
OBFRQ 399.78 MHz
OBSET 4.62 KHz
OBFIN 5.98 Hz
POINT 13107
FREQU 6218.81 Hz
SCANS 32
ACQTM 2.1076 sec
PD 5.0000 sec
PW1 5.17 usec
IRNUC 1H
CTEMP 19.1 c
SLVNT CDCL3
EXREF 7.26 ppm
BF 0.12 Hz
RGAIN 30

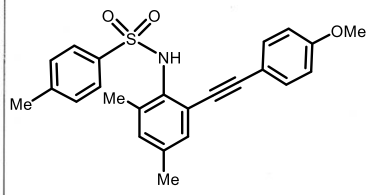

1j

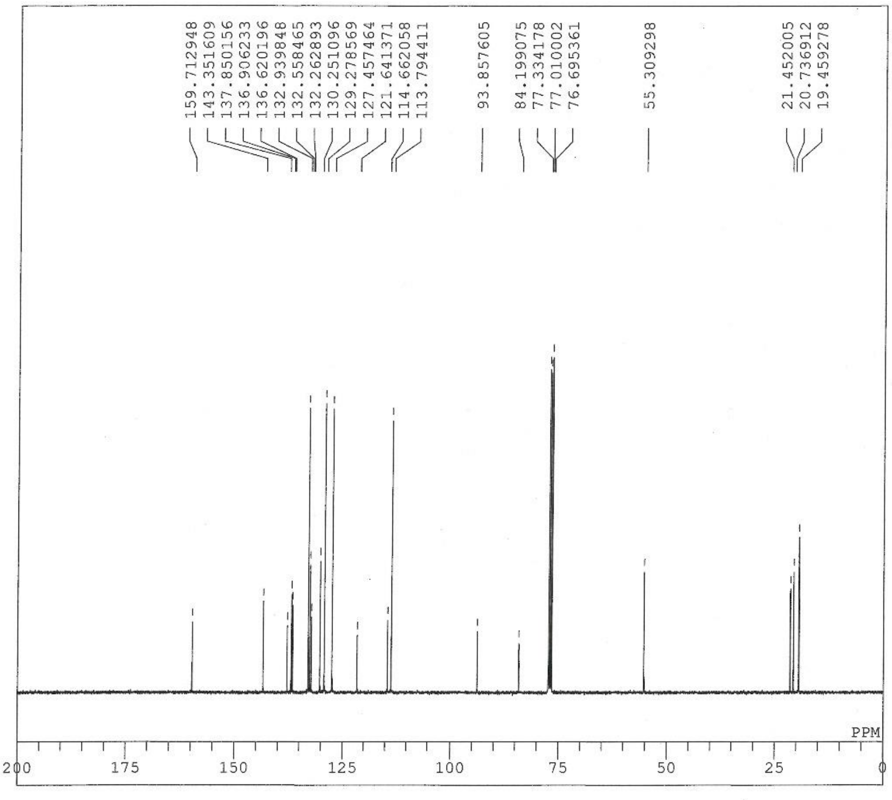

DFILE SF105-13C 20210928-1.als
COMNT
DATIM 2021-09-28 18:58:42
OBNUC 13C
EXMOD single_pulse_dec
OBFRQ 100.53 MHz
OBSET 5.35 KHz
OBFIN 5.86 Hz
POINT 32768
FREQU 31407.03 Hz
SCANS 1024
ACQTM 1.0433 sec
PD 2.0000 sec
PW1 3.03 usec
IRNUC 1H
CTEMP 19.1 c
SLVNT CDCL3
EXREF 77.01 ppm
BF 1.20 Hz
RGAIN 60

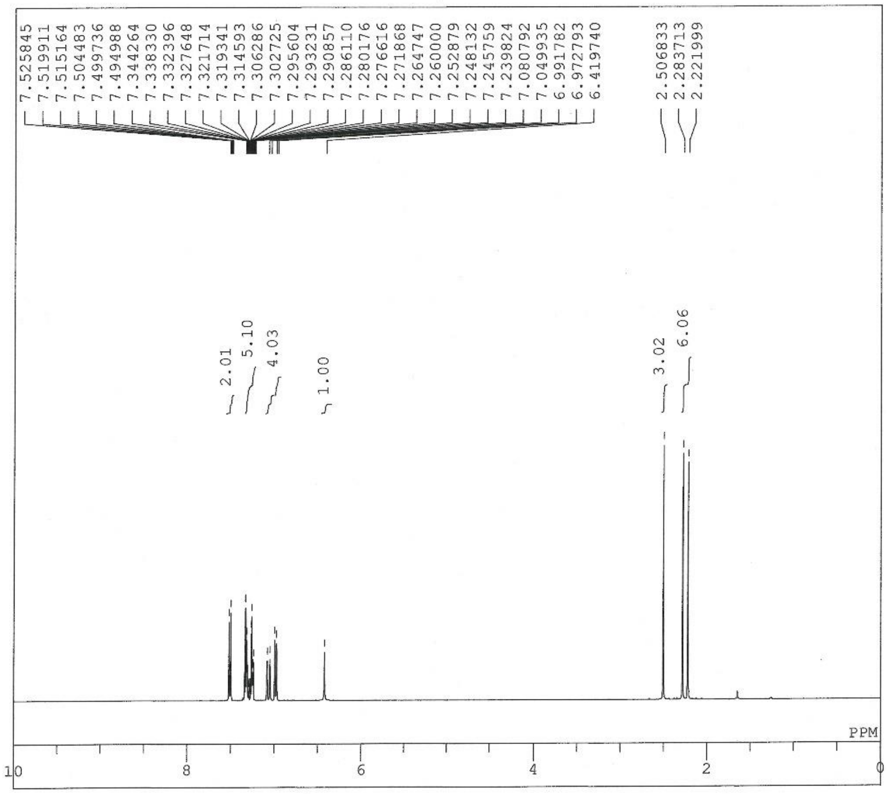

DFILE YO019 20210929-1.als
COMNT Qn-BOC 1H
DATIM 2021-09-29 10:05:24
OBNUC 1H
EXMOD single_pulse.ex2
OBFRQ 399.78 MHz
OBSET 4.62 KHz
OBFIN 5.98 Hz
POINT 13107
FREQU 6218.81 Hz
SCANS 32
ACQTM 2.1076 sec
PD 5.0000 sec
PW1 5.17 usec
IRNUC 1H
CTEMP 18.6 c
SLVNT CDCL3
EXREF 7.26 ppm
BF 0.12 Hz
RGAIN 30

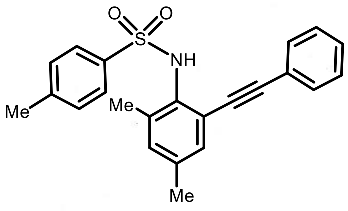

1k

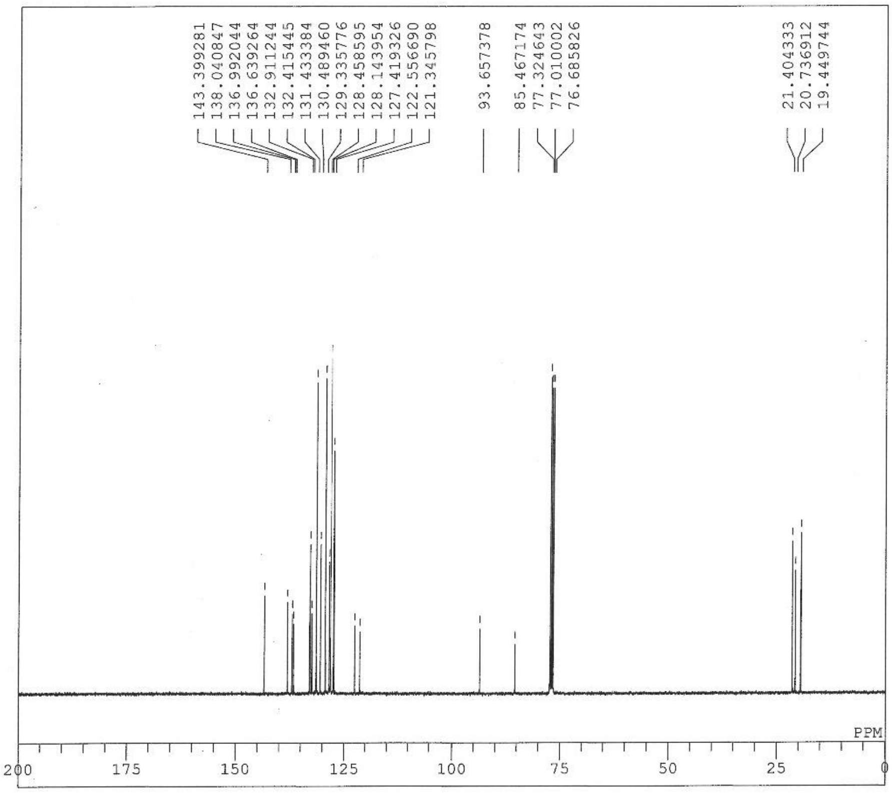

DFILE YO019-13C 20210929-1.als
COMNT
DATIM 2021-09-29 18:13:15
OBNUC 13C
EXMOD single_pulse_dec
OBFRQ 100.53 MHz
OBSET 5.35 KHz
OBFIN 5.86 Hz
POINT 32768
FREQU 31407.03 Hz
SCANS 1024
ACQTM 1.0433 sec
PD 2.0000 sec
PW1 3.03 usec
IRNUC 1H
CTEMP 18.9 c
SLVNT CDCL3
EXREF 77.01 ppm
BF 1.20 Hz
RGAIN 60

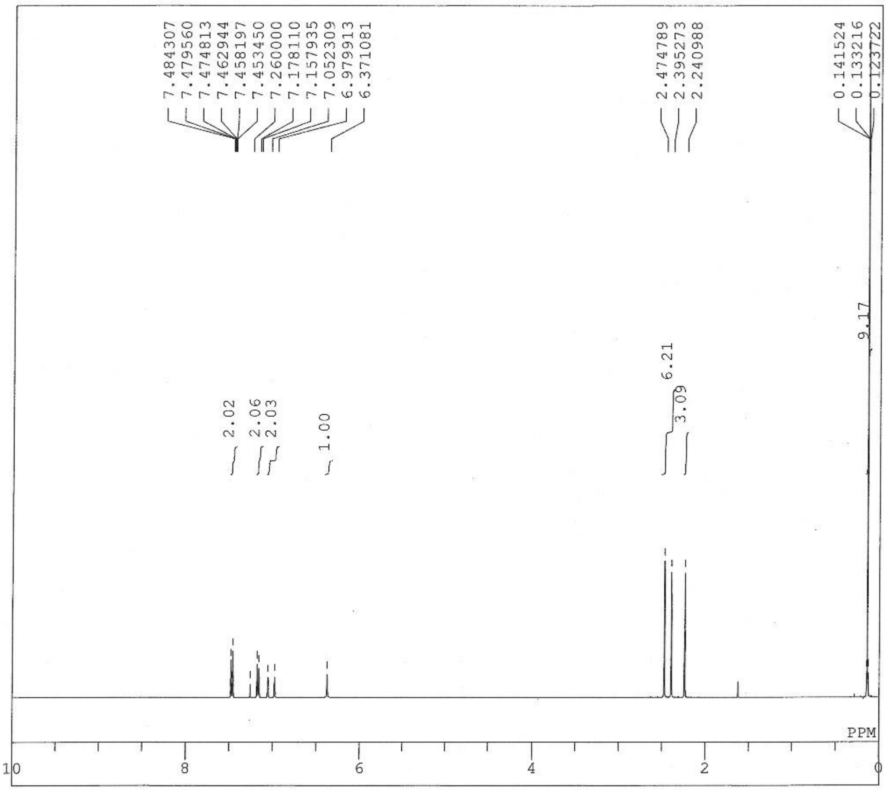

DFILE TT030 20210929-1.als
COMNT Qn-BOC 1H
DATIM 2021-09-29 10:16:13
OBNUC 1H
EXMOD single_pulse.ex2
OBFRQ 399.78 MHz
OBSET 4.62 KHz
OBFIN 5.98 Hz
POINT 13107
FREQU 6218.81 Hz
SCANS 32
ACQTM 2.1076 sec
PD 5.0000 sec
PW1 5.17 usec
IRNUC 1H
CTEMP 19.0 c
SLVNT CDCL3
EXREF 7.26 ppm
BF 0.12 Hz
RGAIN 30

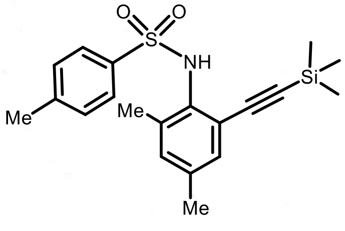

1l

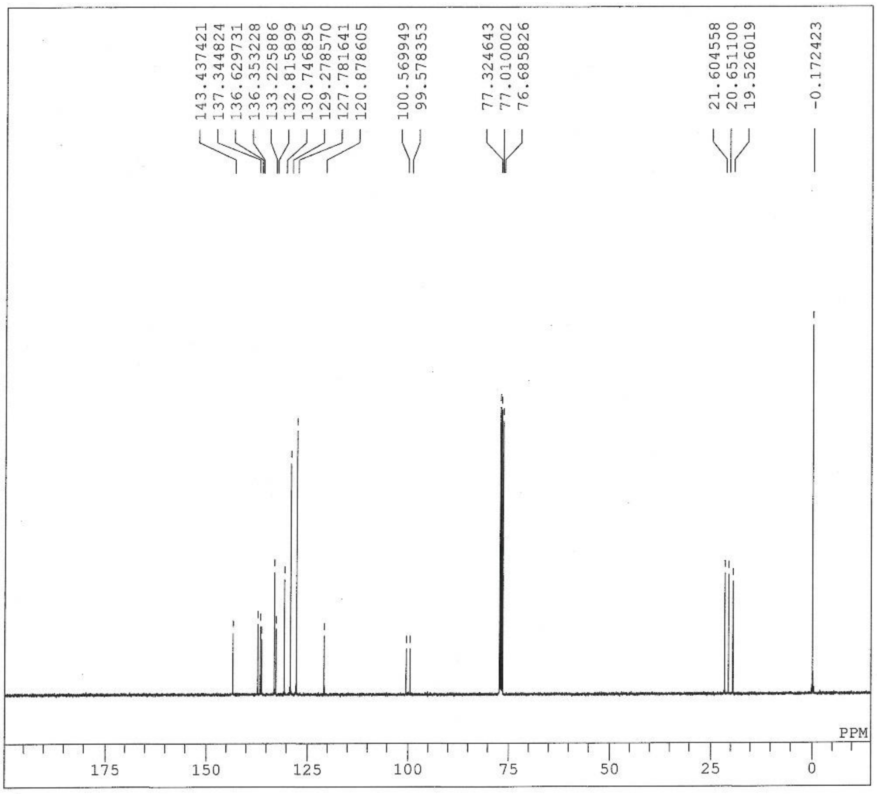

DFILE TT030-13C 20210930-1.als
COMNT
DATIM 2021-09-30 08:58:03
OBNUC 13C
EXMOD single_pulse_dec
OBFRQ 100.53 MHz
OBSET 5.35 KHz
OBFIN 5.86 Hz
POINT 32768
FREQU 31407.03 Hz
SCANS 1024
ACQTM 1.0433 sec
PD 2.0000 sec
PW1 3.03 usec
IRNUC 1H
CTEMP 19.0 c
SLVNT CDCL3
EXREF 77.01 ppm
BF 1.20 Hz
RGAIN 60

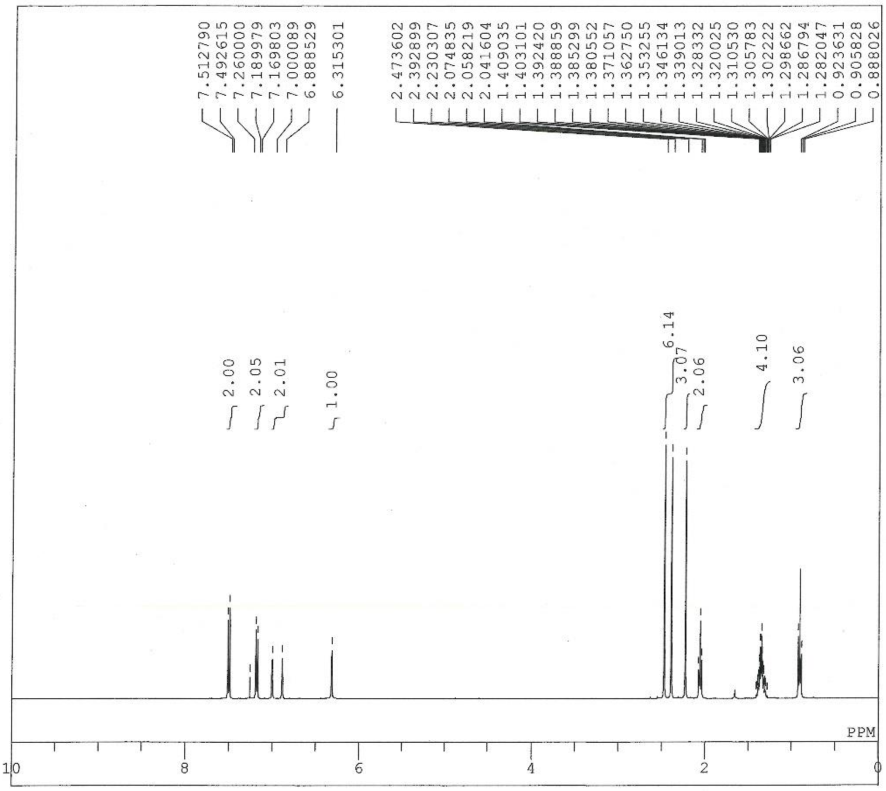

DFILE SF095 20210929-1.als
COMNT Qn-BOC 1H
DATIM 2021-09-29 09:54:42
OBNUC 1H
EXMOD single_pulse.ex2
OBFRQ 399.78 MHz
OBSET 4.62 KHz
OBFIN 5.98 Hz
POINT 13107
FREQU 6218.81 Hz
SCANS 32
ACQTM 2.1076 sec
PD 5.0000 sec
PW1 5.17 usec
IRNUC 1H
CTEMP 18.8 c
SLVNT CDCL3
EXREF 7.26 ppm
BF 0.12 Hz
RGAIN 30

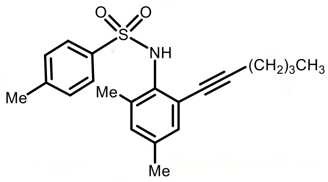

1m

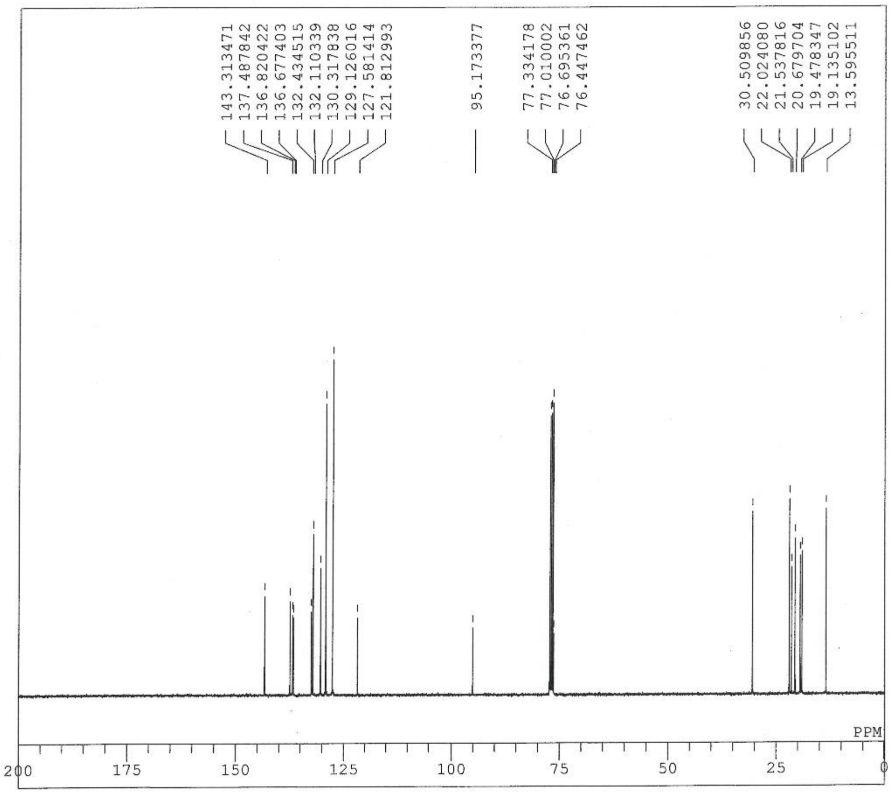

DFILE SF095-13C 20210929-1.als
COMNT
DATIM 2021-09-29 08:43:57
OBNUC 13C
EXMOD single_pulse_dec
OBFRQ 100.53 MHz
OBSET 5.35 KHz
OBFIN 5.86 Hz
POINT 32768
FREQU 31407.03 Hz
SCANS 1024
ACQTM 1.0433 sec
PD 2.0000 sec
PW1 3.03 usec
IRNUC 1H
CTEMP 19.2 c
SLVNT CDCL3
EXREF 77.01 ppm
BF 1.20 Hz
RGAIN 60

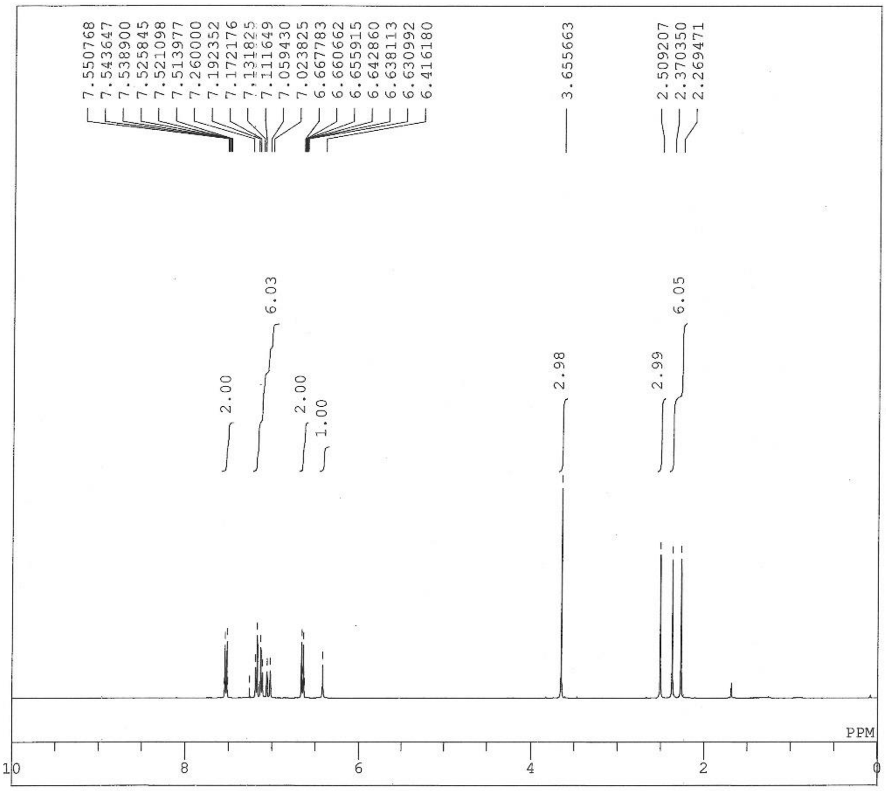

DFILE TF007 20210921-1.als
COMNT Qn-BOC 1H
DATIM 2021-09-21 08:42:19
OBNUC 1H
EXMOD single_pulse.ex2
OBFRQ 399.78 MHz
OBSET 4.62 KHz
OBFIN 5.98 Hz
POINT 13107
FREQU 6218.81 Hz
SCANS 32
ACQTM 2.1076 sec
PD 5.0000 sec
PW1 5.17 usec
IRNUC 1H
CTEMP 19.0 c
SLVNT CDCL3
EXREF 7.26 ppm
BF 0.12 Hz
RGAIN 30

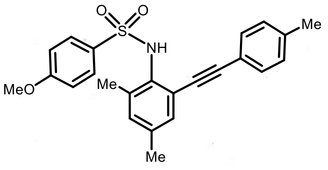

1n

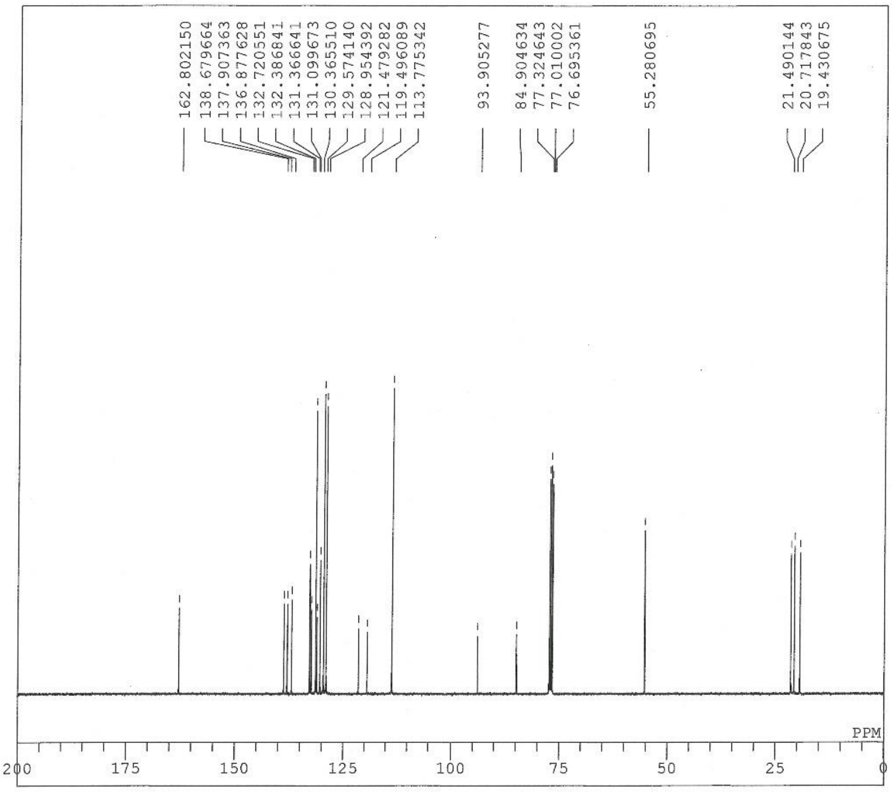

DFILE TF007-13C 20210921-1.als
COMNT
DATIM 2021-09-21 09:41:44
OBNUC 13C
EXMOD single_pulse_dec
OBFRQ 100.53 MHz
OBSET 5.35 KHz
OBFIN 5.86 Hz
POINT 26214
FREQU 25125.24 Hz
SCANS 1024
ACQTM 1.0433 sec
PD 2.0000 sec
PW1 3.03 usec
IRNUC 1H
CTEMP 19.3 c
SLVNT CDCL3
EXREF 77.01 ppm
BF 1.20 Hz
RGAIN 60

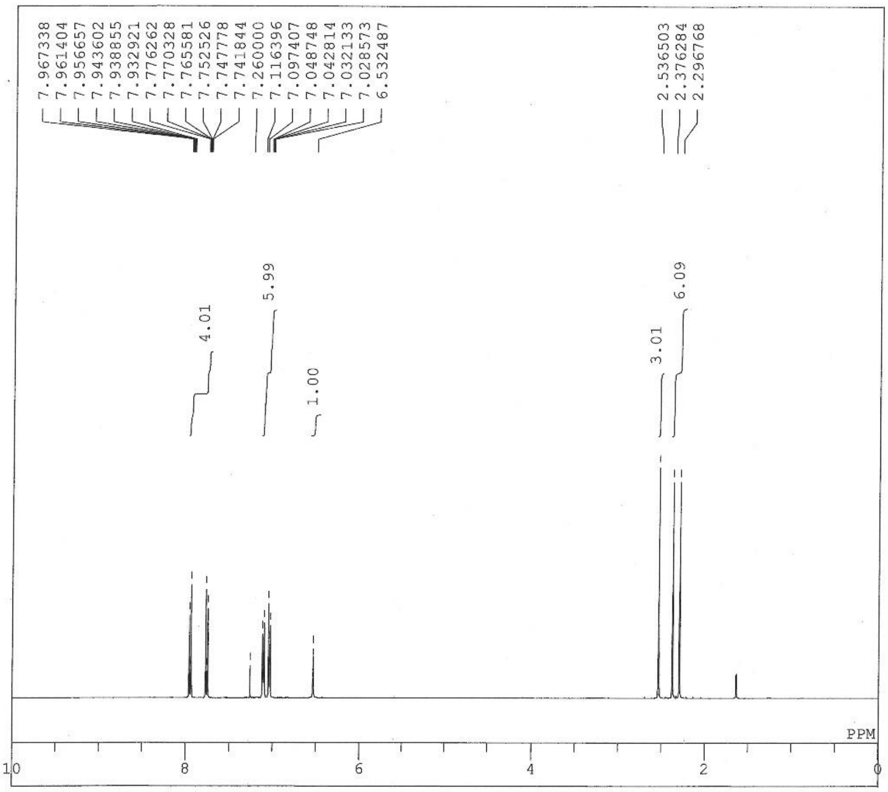

DFILE TT010 20210930-1.als
COMNT Qn-BOC 1H
DATIM 2021-09-30 10:19:17
OBNUC 1H
EXMOD single_pulse.ex2
OBFRQ 399.78 MHz
OBSET 4.62 KHz
OBFIN 5.98 Hz
POINT 13107
FREQU 6218.81 Hz
SCANS 32
ACQTM 2.1076 sec
PD 5.0000 sec
PW1 5.17 usec
IRNUC 1H
CTEMP 19.1 c
SLVNT CDCL3
EXREF 7.26 ppm
BF 0.12 Hz
RGAIN 34

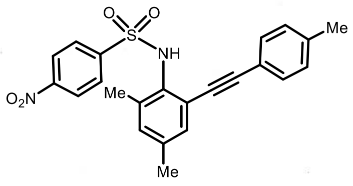

1o

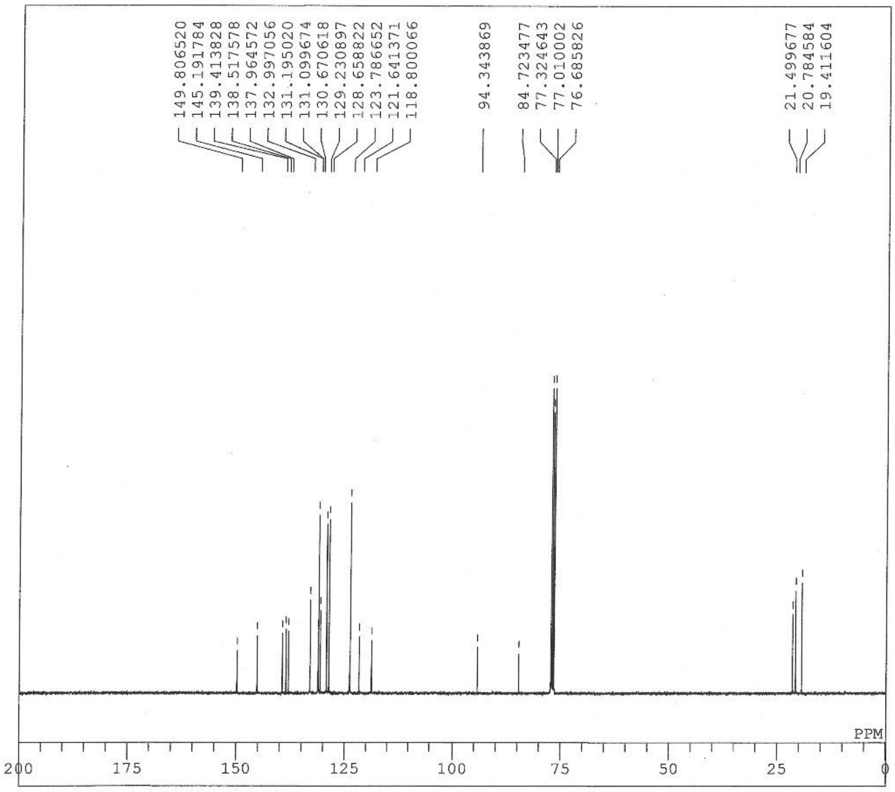

DFILE TT010-13C 20210930-1.als
COMNT
DATIM 2021-09-30 18:12:17
OBNUC 13C
EXMOD single_pulse_dec
OBFRQ 100.53 MHz
OBSET 5.35 KHz
OBFIN 5.86 Hz
POINT 26214
FREQU 25125.24 Hz
SCANS 1024
ACQTM 1.0433 sec
PD 2.0000 sec
PW1 3.03 usec
IRNUC 1H
CTEMP 20.3 c
SLVNT CDCL3
EXREF 77.01 ppm
BF 1.20 Hz
RGAIN 60

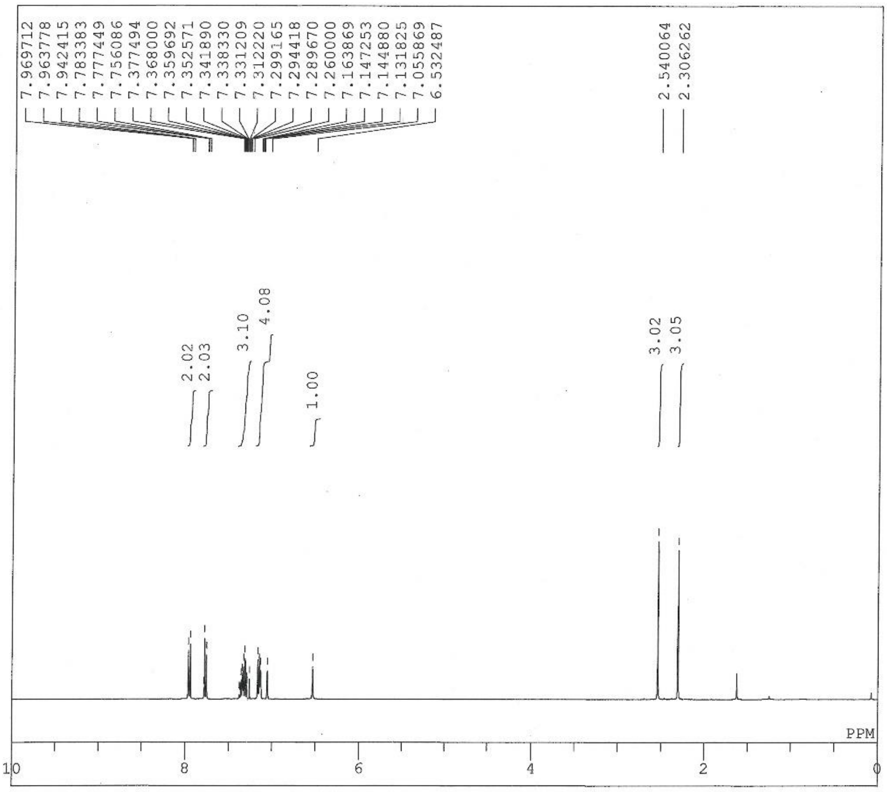

DFILE SF112 20211207-1.als
COMNT Qn-BOC 1H
DATIM 2021-12-06 15:50:34
OBNUC 1H
EXMOD single_pulse.ex2
OBFRQ 399.78 MHz
OBSET 4.62 KHz
OBFIN 5.98 Hz
POINT 13107
FREQU 6218.81 Hz
SCANS 32
ACQTM 2.1076 sec
PD 5.0000 sec
PW1 5.80 usec
IRNUC 1H
CTEMP 18.0 c
SLVNT CDCL3
EXREF 7.26 ppm
BF 0.12 Hz
RGAIN 34

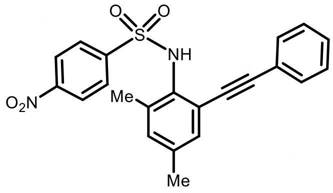

1p

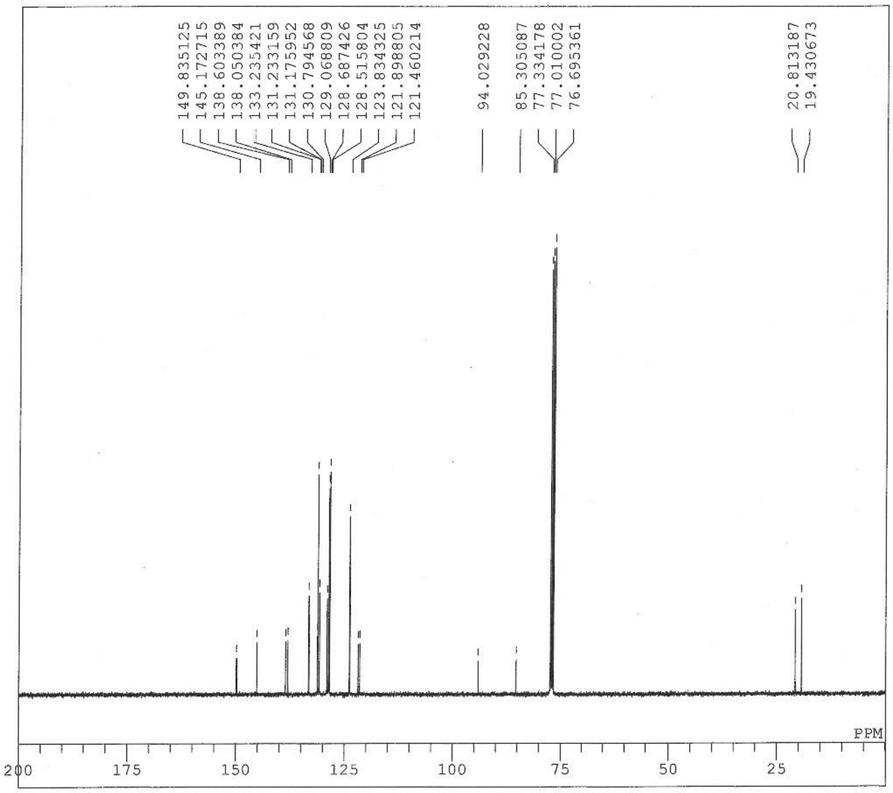

DFILE SF112 13C 20211207-1.als
COMNT
DATIM 2021-12-07 10:32:06
OBNUC 13C
EXMOD single_pulse_dec
OBFRQ 100.53 MHz
OBSET 5.35 KHz
OBFIN 5.86 Hz
POINT 26214
FREQU 25125.24 Hz
SCANS 1024
ACQTM 1.0433 sec
PD 2.0000 sec
PW1 3.23 usec
IRNUC 1H
CTEMP 18.1 c
SLVNT CDCL3
EXREF 77.01 ppm
BF 1.20 Hz
RGAIN 60

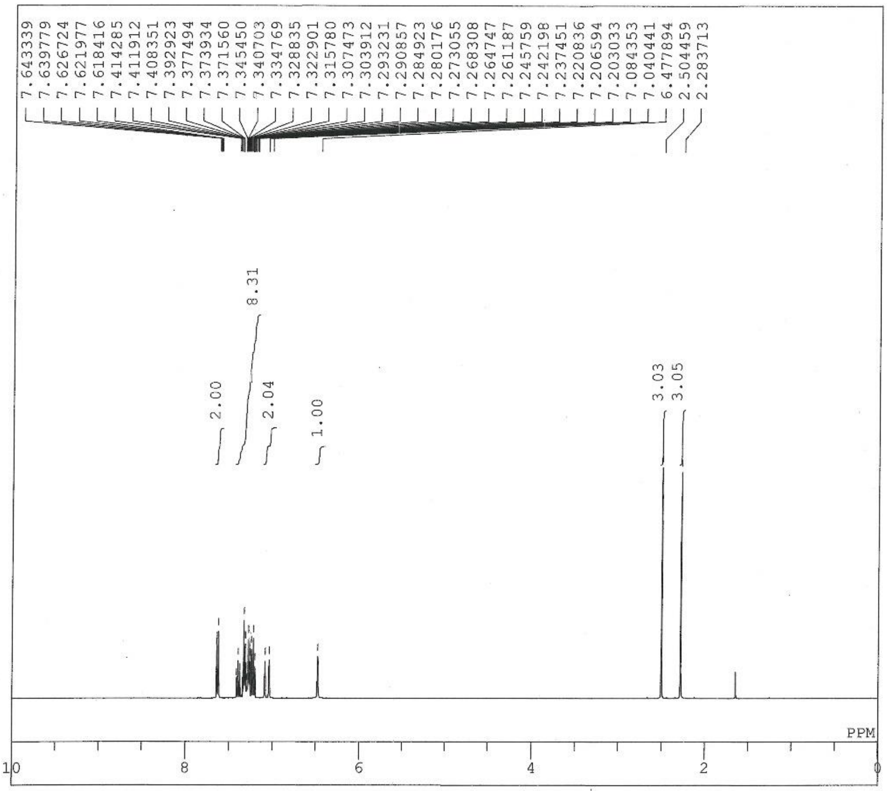

DFILE SF121 20210930-1.als
COMNT Qn-BOC 1H
DATIM 2021-09-30 10:30:05
OBNUC 1H
EXMOD single_pulse.ex2
OBFRQ 399.78 MHz
OBSET 4.62 KHz
OBFIN 5.98 Hz
POINT 13107
FREQU 6218.81 Hz
SCANS 32
ACQTM 2.1076 sec
PD 5.0000 sec
PW1 5.17 usec
IRNUC 1H
CTEMP 18.9 c
SLVNT CDCL3
EXREF 7.26 ppm
BF 0.12 Hz
RGAIN 30

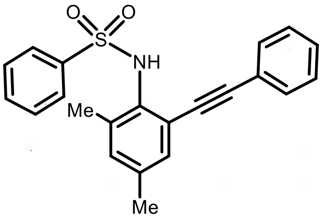

1q

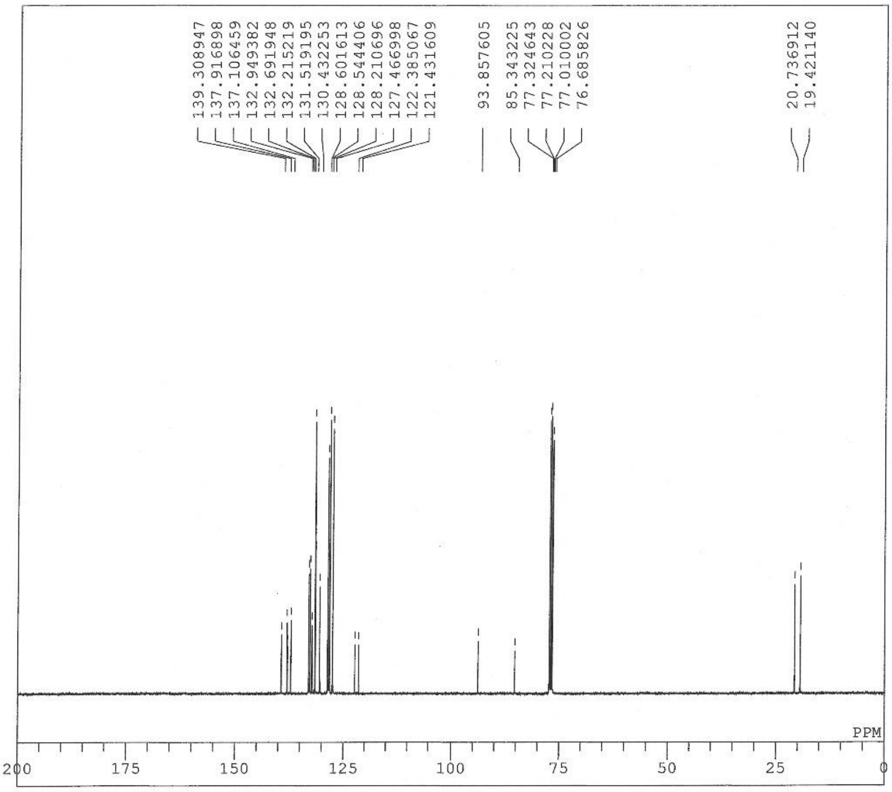

DFILE SF121-13C 20211002-1.als
COMNT
DATIM 2021-10-02 09:53:39
OBNUC 13C
EXMOD single_pulse_dec
OBFRQ 100.53 MHz
OBSET 5.35 KHz
OBFIN 5.86 Hz
POINT 26214
FREQU 25125.24 Hz
SCANS 1024
ACQTM 1.0433 sec
PD 2.0000 sec
PW1 3.03 usec
IRNUC 1H
CTEMP 19.0 c
SLVNT CDCL3
EXREF 77.01 ppm
BF 1.20 Hz
RGAIN 60

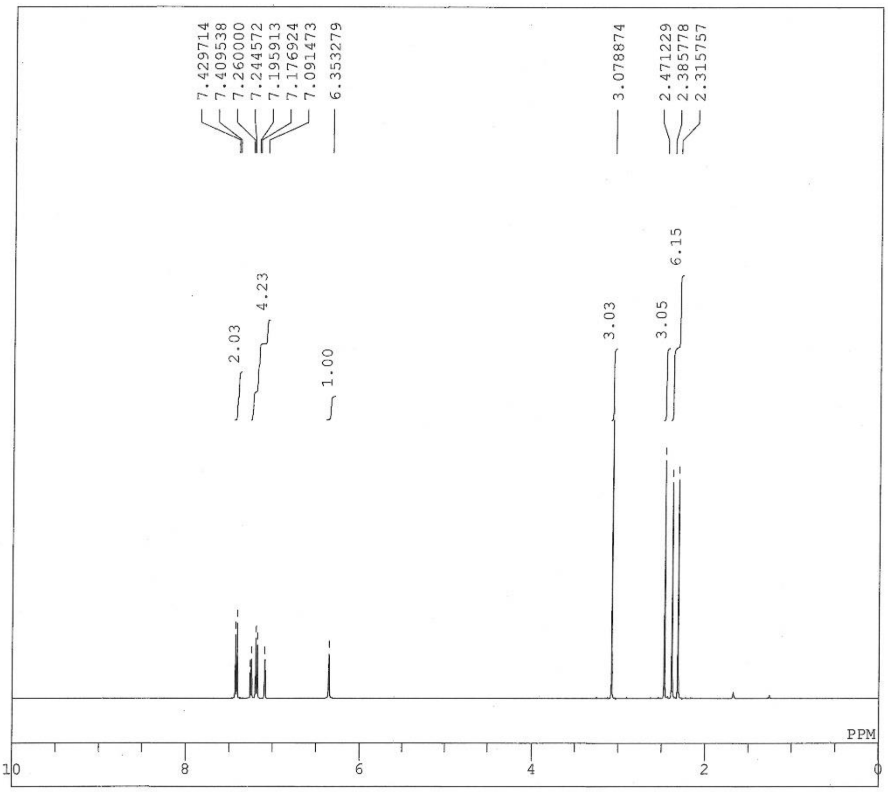

DFILE	SF088 20211213-1.als
COMNT	Qn-BOC 1H
DATIM	2021-12-13 13:28:17
OBNUC	1H
EXMOD	single_pulse.ex2
OBFRQ	399.78 MHz
OBSET	4.62 KHz
OBFIN	5.98 Hz
POINT	16384
FREQU	7773.63 Hz
SCANS	32
ACQTM	2.1076 sec
PD	5.0000 sec
PW1	5.80 usec
IRNUC	1H
CTEMP	18.5 c
SLVNT	CDCL3
EXREF	7.26 ppm
BF	0.12 Hz
RGAIN	30

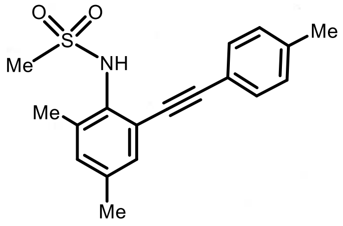

1r

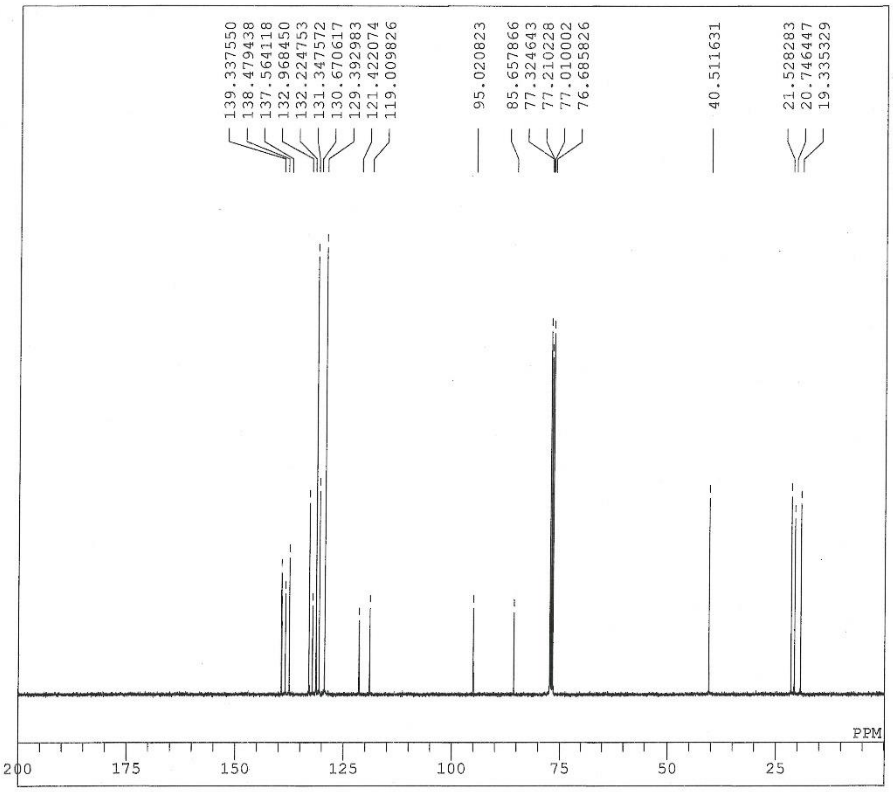

DFILE	SF088 13C 20211213-1.als
COMNT	
DATIM	2021-12-13 09:38:13
OBNUC	13C
EXMOD	single_pulse_dec
OBFRQ	100.53 MHz
OBSET	5.35 KHz
OBFIN	5.86 Hz
POINT	32768
FREQU	31407.03 Hz
SCANS	1024
ACQTM	1.0433 sec
PD	2.0000 sec
PW1	3.23 usec
IRNUC	1H
CTEMP	18.5 c
SLVNT	CDCL3
EXREF	77.01 ppm
BF	1.20 Hz
RGAIN	60

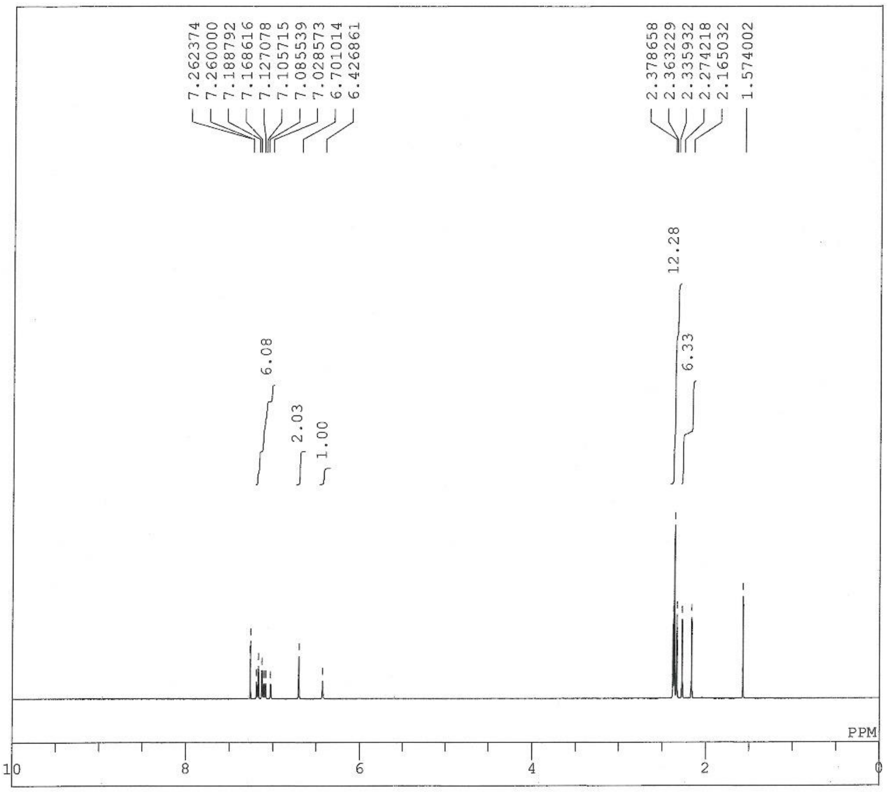

DFILE SF102 20211129-1.als
COMNT Qn-BOC 1H
DATIM 2021-11-29 11:57:15
OBNUC 1H
EXMOD single_pulse.ex2
OBFRQ 399.78 MHz
OBSET 4.62 KHz
OBFIN 5.98 Hz
POINT 13107
FREQU 6218.81 Hz
SCANS 32
ACQTM 2.1076 sec
PD 5.0000 sec
PW1 5.80 usec
IRNUC 1H
CTEMP 17.8 c
SLVNT CDCL3
EXREF 7.26 ppm
BF 0.12 Hz
RGAIN 36

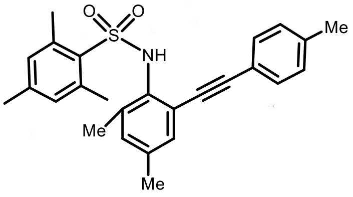

1s

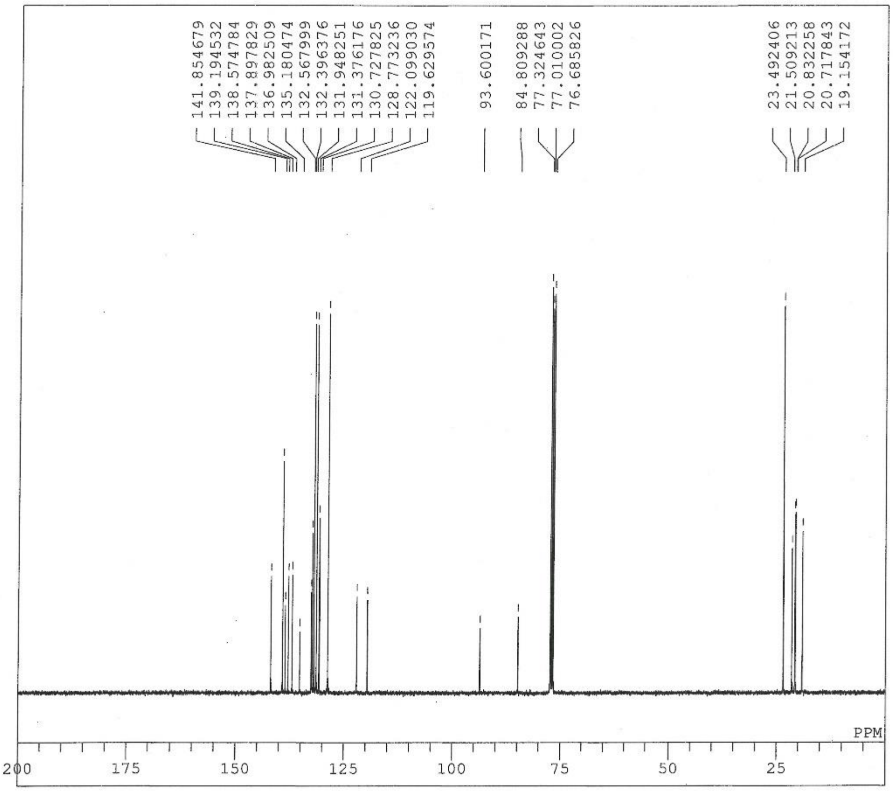

DFILE SF102 13C-2 20211130-1.a
COMNT
DATIM 2021-11-30 18:17:36
OBNUC 13C
EXMOD single_pulse_dec
OBFRQ 100.53 MHz
OBSET 5.35 KHz
OBFIN 5.86 Hz
POINT 26214
FREQU 25125.24 Hz
SCANS 1024
ACQTM 1.0433 sec
PD 2.0000 sec
PW1 3.23 usec
IRNUC 1H
CTEMP 19.8 c
SLVNT CDCL3
EXREF 77.01 ppm
BF 1.20 Hz
RGAIN 60

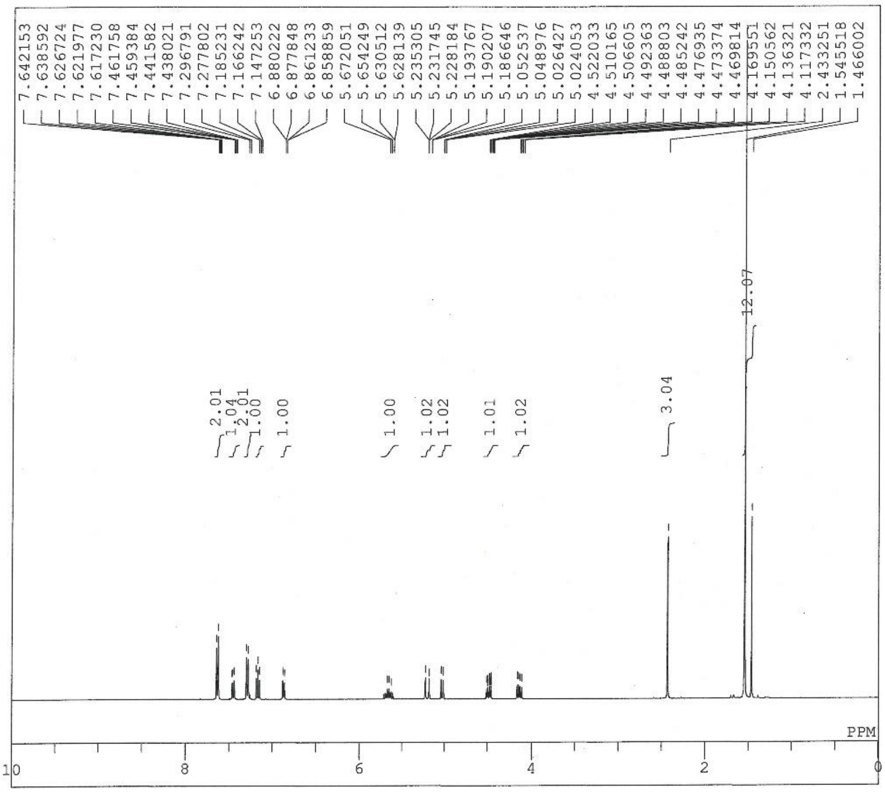

DFILE YO015 20211008-1.als
COMNT Qn-BOC 1H
DATIM 2021-10-08 13:58:12
OBNUC 1H
EXMOD single_pulse.ex2
OBFRQ 399.78 MHz
OBSET 4.62 KHz
OBFIN 5.98 Hz
POINT 13107
FREQU 6218.81 Hz
SCANS 32
ACQTM 2.1076 sec
PD 5.0000 sec
PW1 5.17 usec
IRNUC 1H
CTEMP 18.5 c
SLVNT CDCL3
EXREF 7.26 ppm
BF 0.12 Hz
RGAIN 20

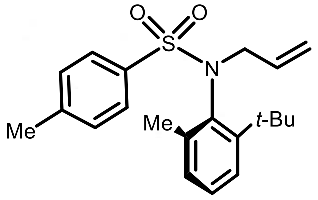

2a

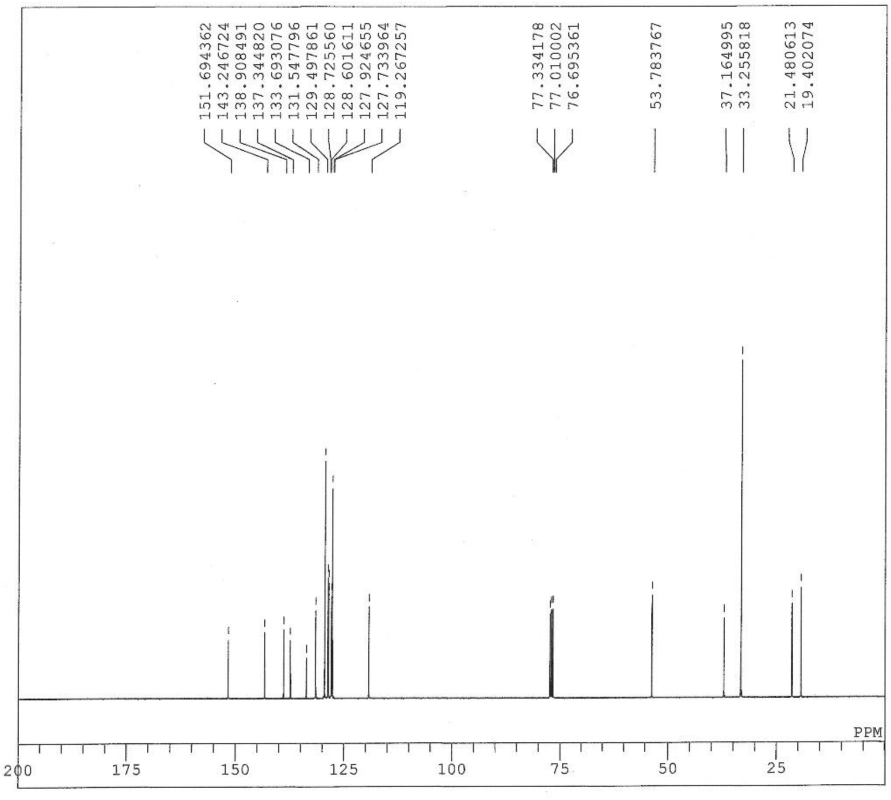

DFILE YO015-13C 20211008-1.als
COMNT
DATIM 2021-10-08 17:31:11
OBNUC 13C
EXMOD single_pulse_dec
OBFRQ 100.53 MHz
OBSET 5.35 KHz
OBFIN 5.86 Hz
POINT 32768
FREQU 31407.03 Hz
SCANS 1024
ACQTM 1.0433 sec
PD 2.0000 sec
PW1 3.03 usec
IRNUC 1H
CTEMP 19.3 c
SLVNT CDCL3
EXREF 77.01 ppm
BF 1.20 Hz
RGAIN 60

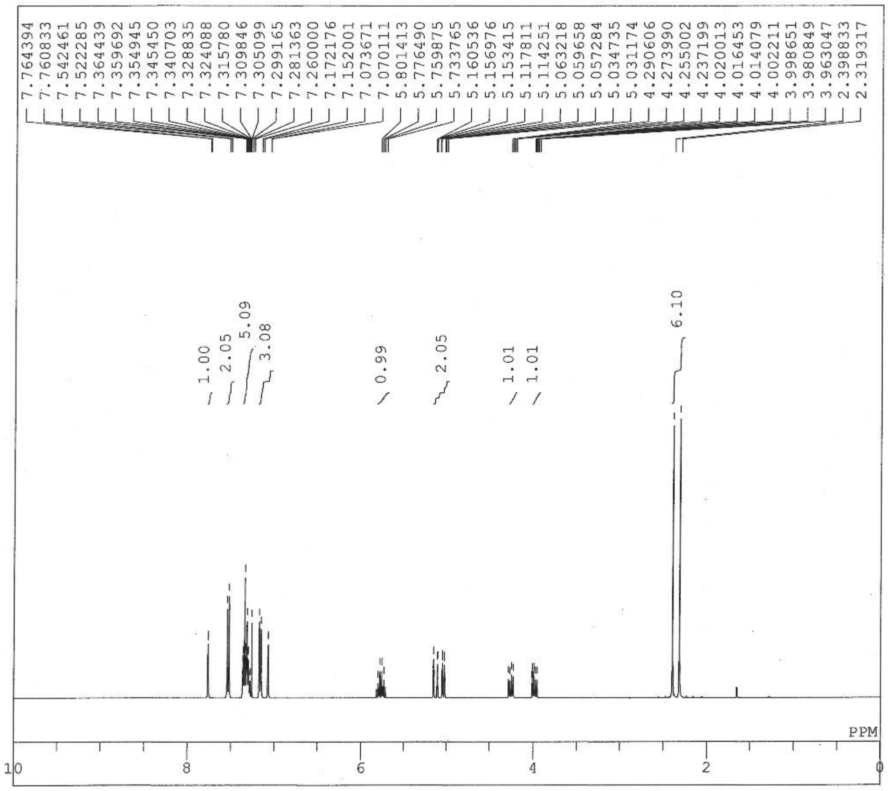

DFILE TT088 20211111-1.als
COMNT Qn-BOC 1H
DATIM 2021-11-11 15:20:11
OBNUC 1H
EXMOD single_pulse.ex2
OBFRQ 399.78 MHz
OBSET 4.62 KHz
OBFIN 5.98 Hz
POINT 13107
FREQU 6218.81 Hz
SCANS 32
ACQTM 2.1076 sec
PD 5.0000 sec
PW1 5.17 usec
IRNUC 1H
CTEMP 19.1 c
SLVNT CDCL3
EXREF 7.26 ppm
BF 0.12 Hz
RGAIN 24

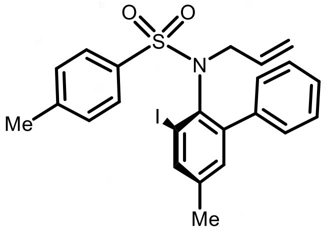

2b

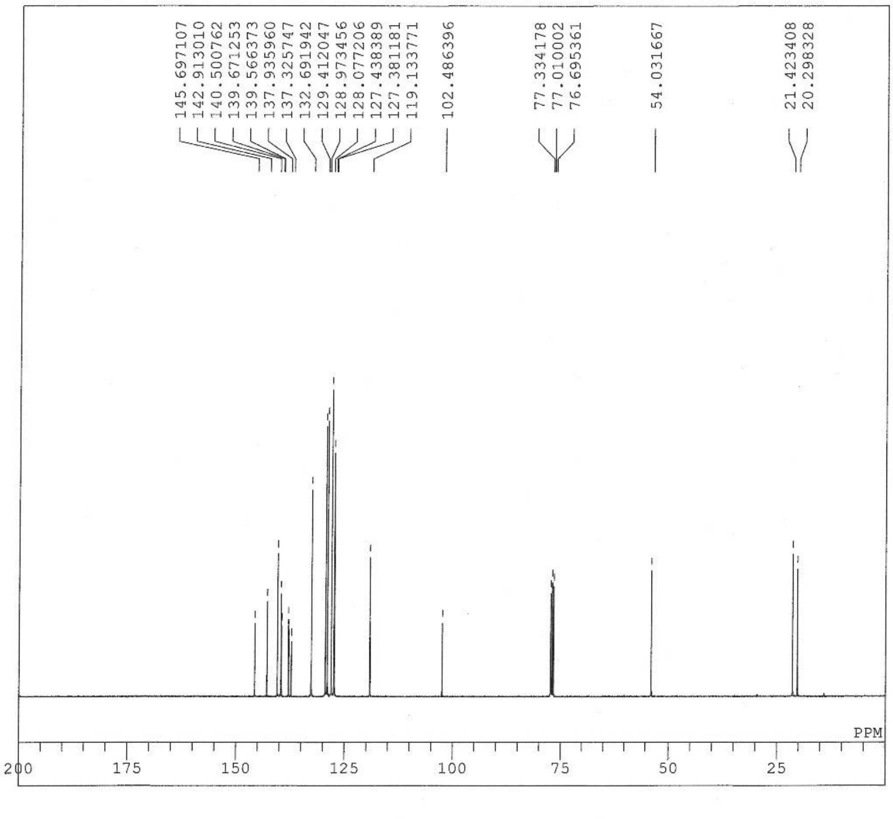

DFILE TT088 carbon 20211027-1.
COMNT
DATIM 2021-10-27 20:34:04
OBNUC 13C
EXMOD single_pulse_dec
OBFRQ 100.53 MHz
OBSET 5.35 KHz
OBFIN 5.86 Hz
POINT 26214
FREQU 25125.24 Hz
SCANS 1024
ACQTM 1.0433 sec
PD 2.0000 sec
PW1 3.03 usec
IRNUC 1H
CTEMP 18.5 c
SLVNT CDCL3
EXREF 77.01 ppm
BF 1.20 Hz
RGAIN 60

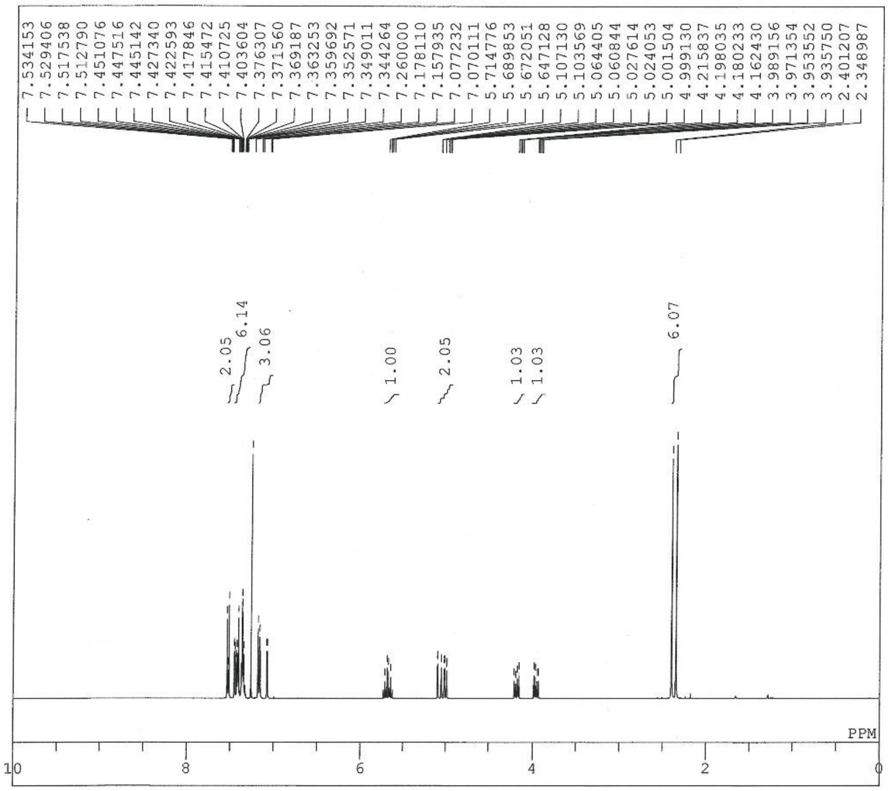

DFILE SF136 20211025-1.als
COMNT Qn-BOC 1H
DATIM 2021-10-25 15:16:18
OBNUC 1H
EXMOD single_pulse.ex2
OBFRQ 399.78 MHz
OBSET 4.62 KHz
OBFIN 5.98 Hz
POINT 16384
FREQU 7773.63 Hz
SCANS 32
ACQTM 2.1076 sec
PD 5.0000 sec
PW1 5.17 usec
IRNUC 1H
CTEMP 17.6 c
SLVNT CDCL3
EXREF 7.26 ppm
BF 0.12 Hz
RGAIN 24

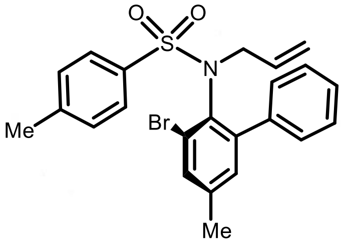

2c

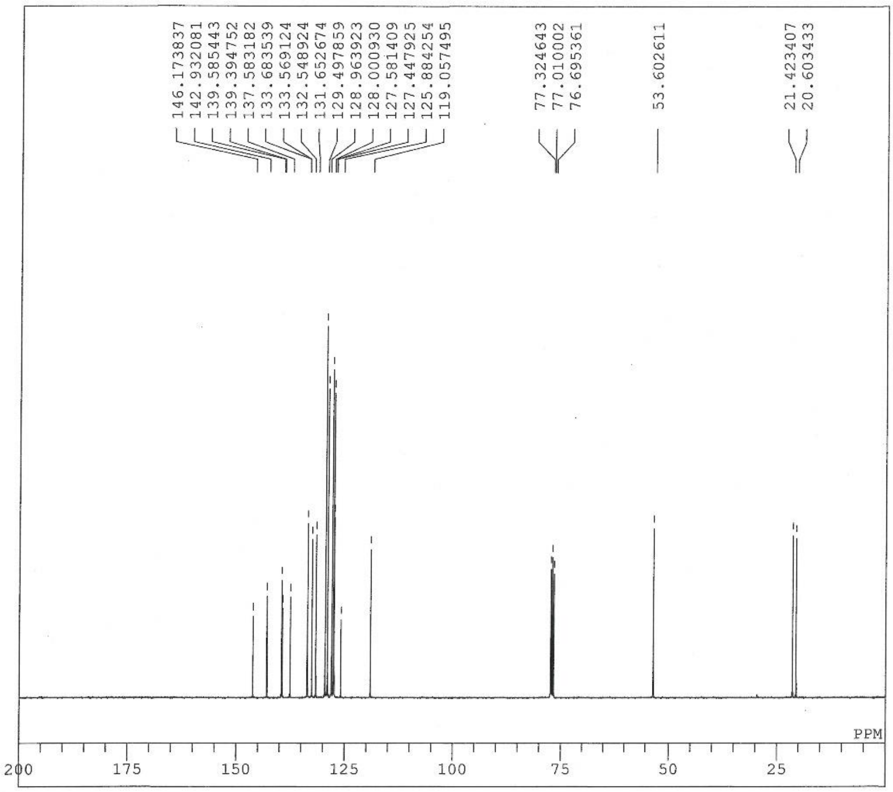

DFILE SF136-13C 20211025-1.als
COMNT
DATIM 2021-10-25 18:54:20
OBNUC 13C
EXMOD single_pulse_dec
OBFRQ 100.53 MHz
OBSET 5.35 KHz
OBFIN 5.86 Hz
POINT 32768
FREQU 31407.03 Hz
SCANS 1024
ACQTM 1.0433 sec
PD 2.0000 sec
PW1 3.03 usec
IRNUC 1H
CTEMP 18.1 c
SLVNT CDCL3
EXREF 77.01 ppm
BF 1.20 Hz
RGAIN 60

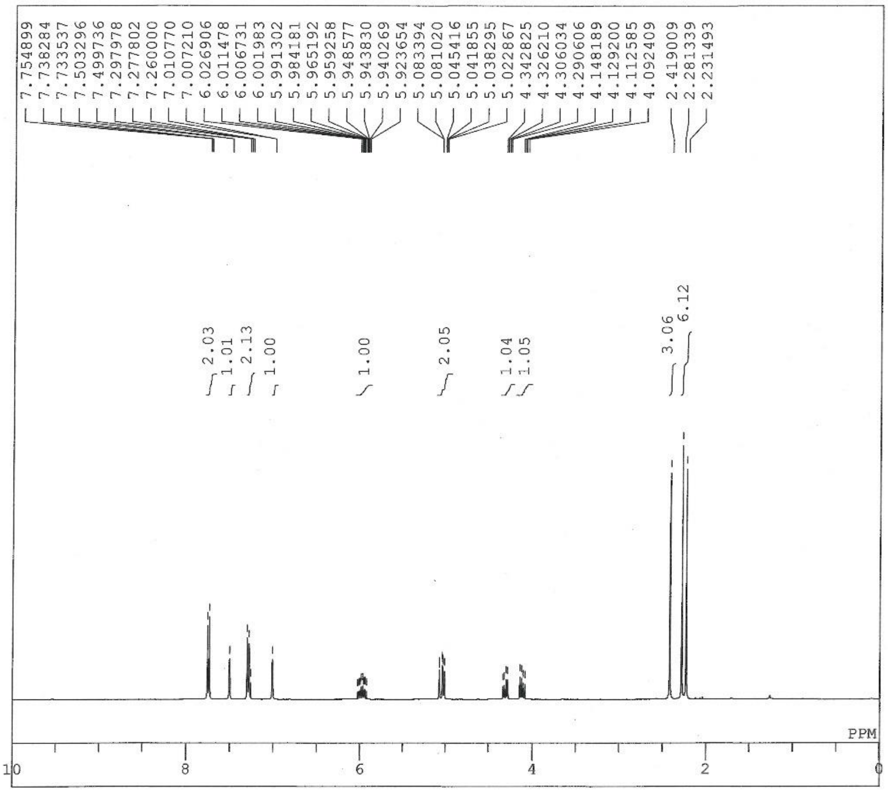

DFILE	RK036 20211029-1.als
COMNT	Qn-BOC 1H
DATIM	2021-10-29 16:08:06
OBNUC	1H
EXMOD	single_pulse.ex2
OBFRQ	399.78 MHz
OBSET	4.62 KHz
OBFIN	5.98 Hz
POINT	16384
FREQU	7773.63 Hz
SCANS	32
ACQTM	2.1076 sec
PD	5.0000 sec
PW1	5.17 usec
IRNUC	1H
CTEMP	18.7 c
SLVNT	CDCL3
EXREF	7.26 ppm
BF	0.12 Hz
RGAIN	22

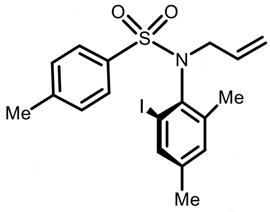

2d

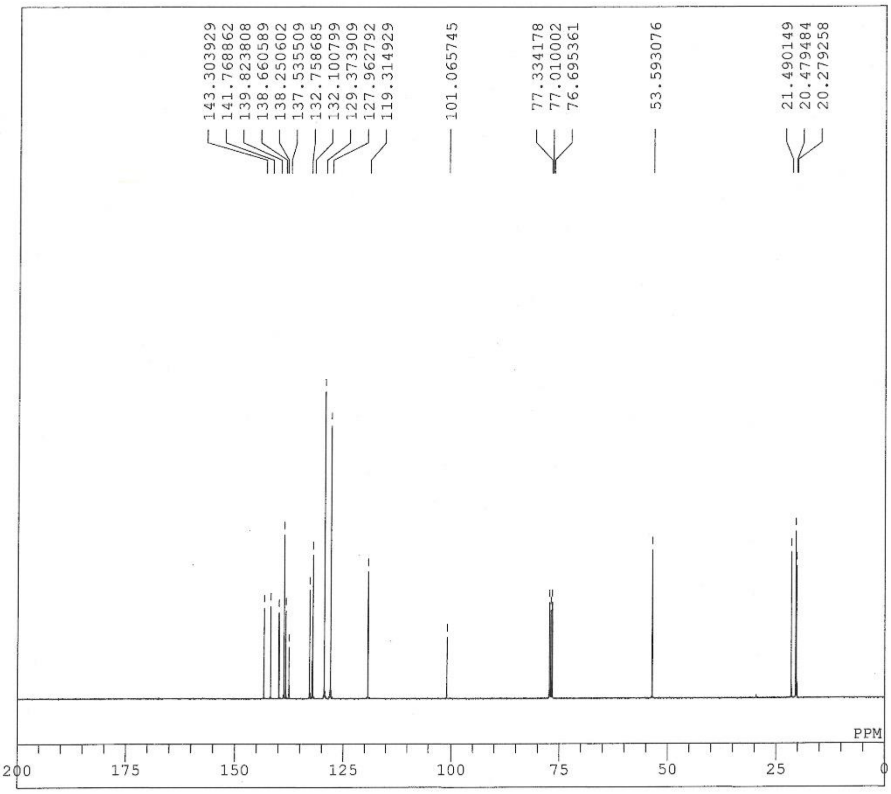

DFILE	RK036-13C 20211102-1.als
COMNT	
DATIM	2021-11-02 09:27:43
OBNUC	13C
EXMOD	single_pulse_dec
OBFRQ	100.53 MHz
OBSET	5.35 KHz
OBFIN	5.86 Hz
POINT	32768
FREQU	31407.03 Hz
SCANS	1024
ACQTM	1.0433 sec
PD	2.0000 sec
PW1	3.03 usec
IRNUC	1H
CTEMP	18.5 c
SLVNT	CDCL3
EXREF	77.01 ppm
BF	1.20 Hz
RGAIN	60

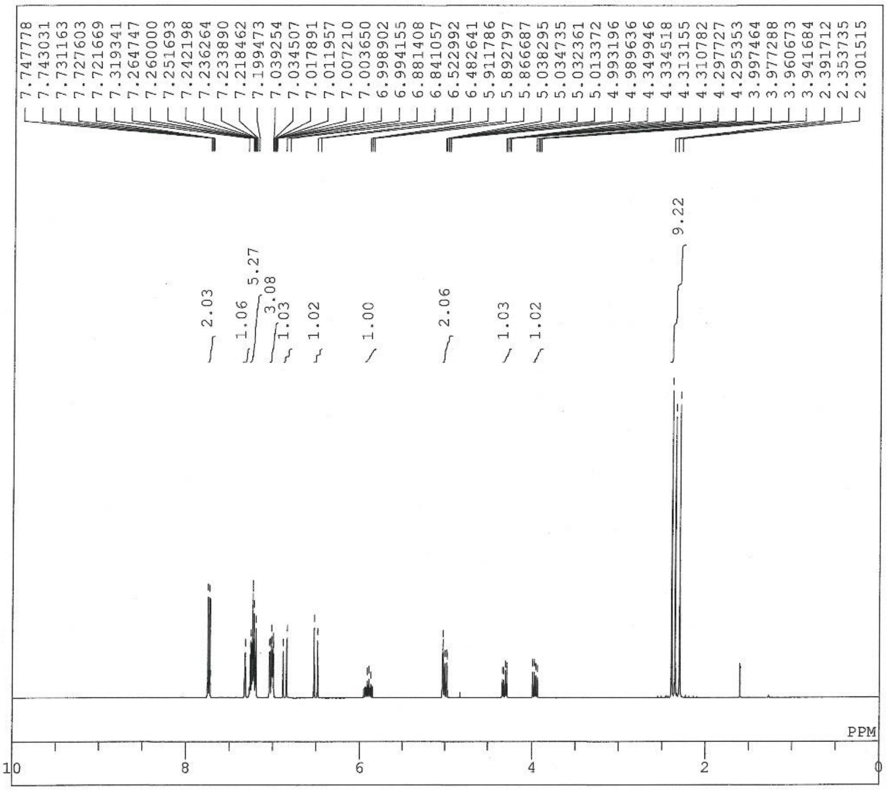

DFILE TT083 20211014-1.als
COMNT Qn-BOC 1H
DATIM 2021-10-14 15:49:22
OBNUC 1H
EXMOD single_pulse.ex2
OBFRQ 399.78 MHz
OBSET 4.62 KHz
OBFIN 5.98 Hz
POINT 16384
FREQU 7773.63 Hz
SCANS 32
ACQTM 2.1076 sec
PD 5.0000 sec
PW1 5.17 usec
IRNUC 1H
CTEMP 18.6 c
SLVNT CDCL3
EXREF 7.26 ppm
BF 0.12 Hz
RGAIN 30

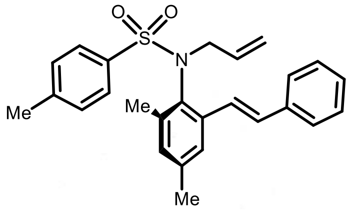

2e

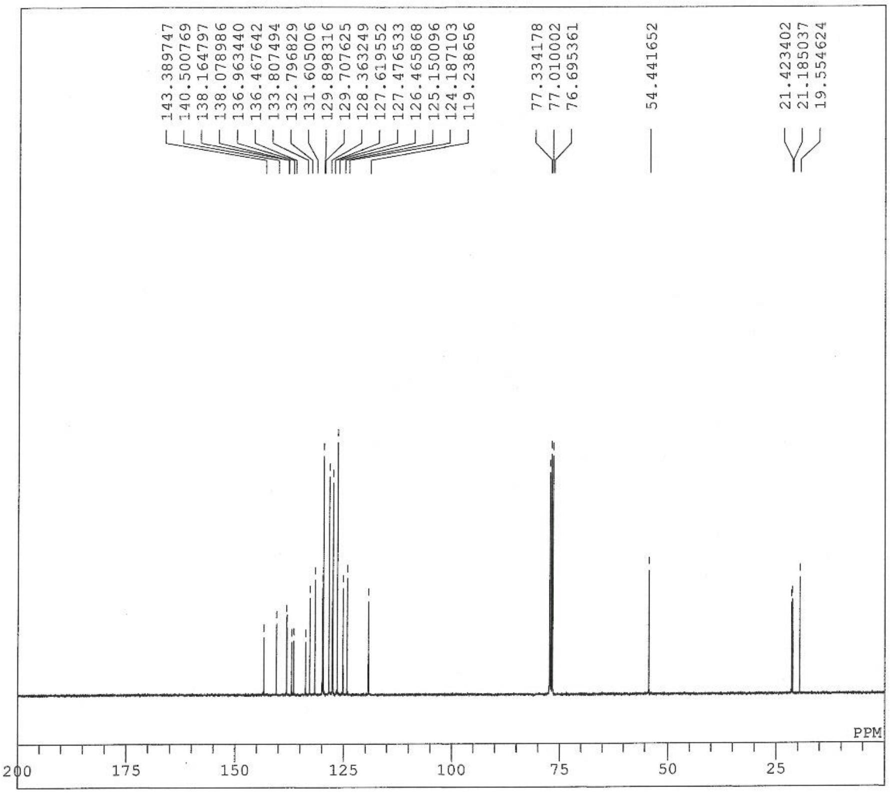

DFILE TT083-13C 20211015-1.als
COMNT
DATIM 2021-10-15 09:11:14
OBNUC 13C
EXMOD single_pulse_dec
OBFRQ 100.53 MHz
OBSET 5.35 KHz
OBFIN 5.86 Hz
POINT 32768
FREQU 31407.03 Hz
SCANS 1024
ACQTM 1.0433 sec
PD 2.0000 sec
PW1 3.03 usec
IRNUC 1H
CTEMP 19.0 c
SLVNT CDCL3
EXREF 77.01 ppm
BF 1.20 Hz
RGAIN 60

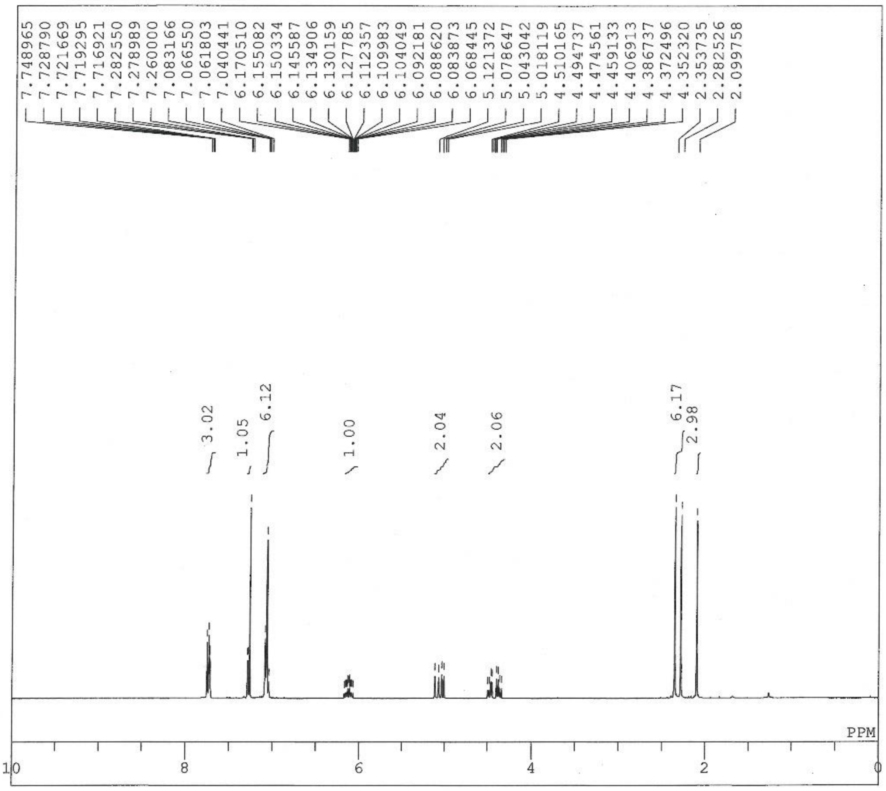

DFILE RK040 20211125-1.als
COMNT Qn-BOC 1H
DATIM 2021-11-25 14:03:08
OBNUC 1H
EXMOD single_pulse.ex2
OBFRQ 399.78 MHz
OBSET 4.62 KHz
OBFIN 5.98 Hz
POINT 16384
FREQU 7773.63 Hz
SCANS 32
ACQTM 2.1076 sec
PD 5.0000 sec
PW1 5.80 usec
IRNUC 1H
CTEMP 18.6 c
SLVNT CDCL3
EXREF 7.26 ppm
BF 0.12 Hz
RGAIN 26

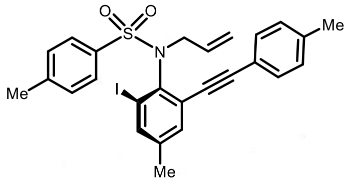

2f

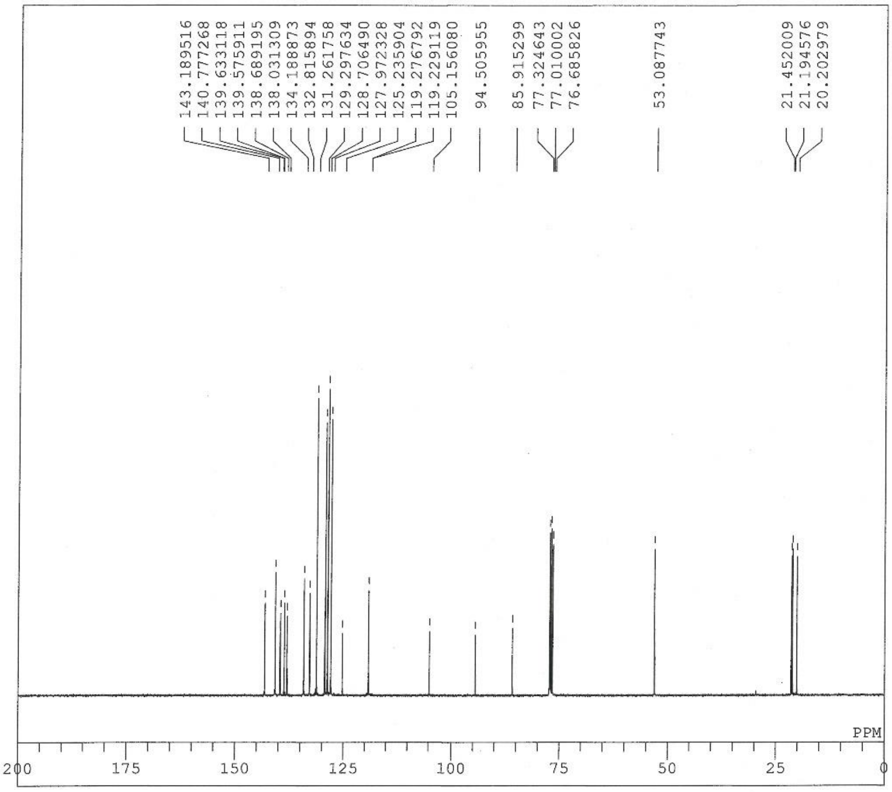

DFILE RK040-13C 20211126-1.jdf
COMNT
DATIM 2021-11-26 17:57:28
OBNUC 13C
EXMOD single_pulse_dec
OBFRQ 100.53 MHz
OBSET 5.35 KHz
OBFIN 5.86 Hz
POINT 32768
FREQU 31407.03 Hz
SCANS 1024
ACQTM 1.0433 sec
PD 2.0000 sec
PW1 3.23 usec
IRNUC 1H
CTEMP 19.3 c
SLVNT CDCL3
EXREF 77.01 ppm
BF 1.20 Hz
RGAIN 60

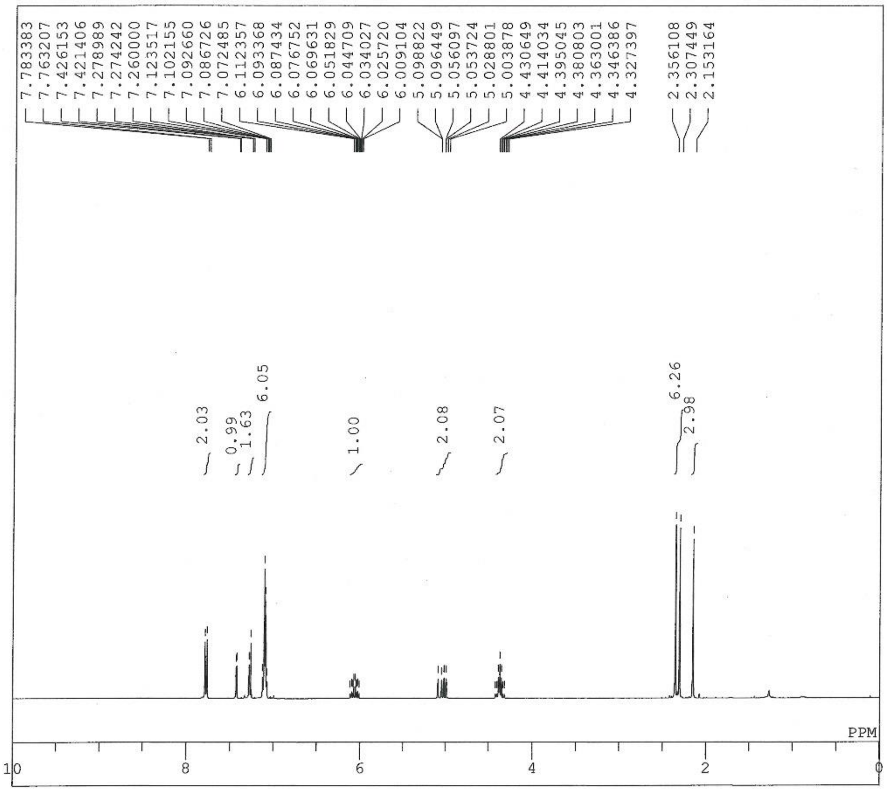

DFILE	RK033 20211023-1.als
COMNT	Qn-BOC 1H
DATIM	2021-10-23 12:56:30
OBNUC	1H
EXMOD	single_pulse.ex2
OBFRQ	399.78 MHz
OBSET	4.62 KHz
OBFIN	5.98 Hz
POINT	13107
FREQU	6218.81 Hz
SCANS	32
ACQTM	2.1076 sec
PD	5.0000 sec
PW1	5.17 usec
IRNUC	1H
CTEMP	18.1 c
SLVNT	CDCL3
EXREF	7.26 ppm
BF	0.12 Hz
RGAIN	22

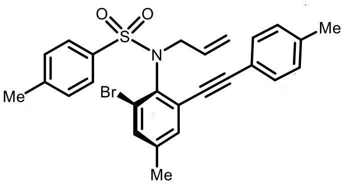

2g

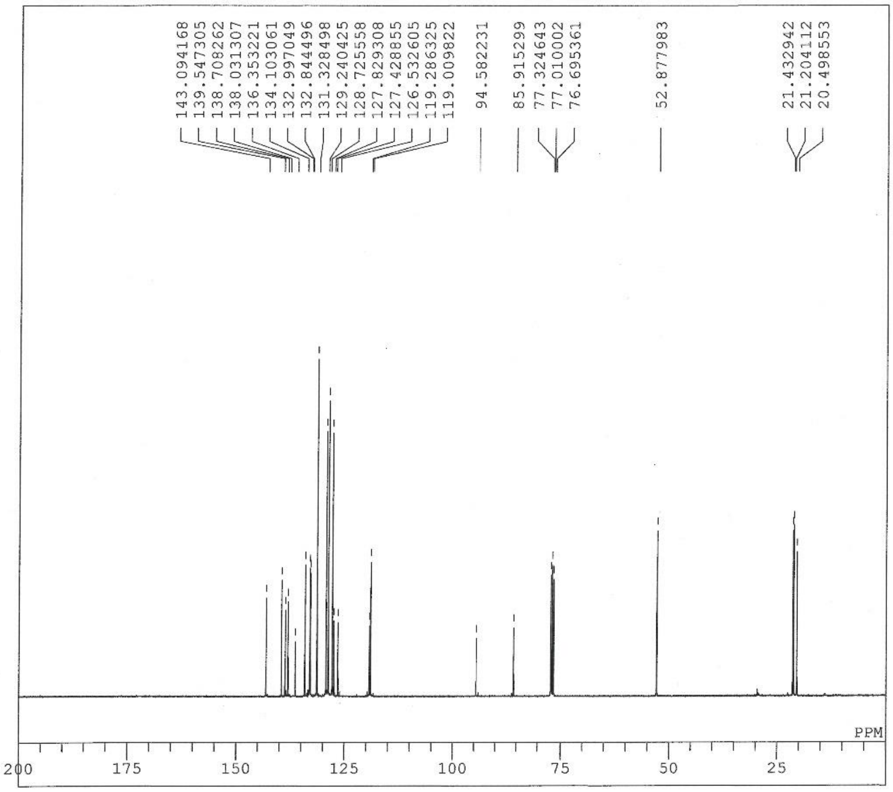

DFILE	RK033-13C 20211023-1.als
COMNT	
DATIM	2021-10-23 10:49:27
OBNUC	13C
EXMOD	single_pulse_dec
OBFRQ	100.53 MHz
OBSET	5.35 KHz
OBFIN	5.86 Hz
POINT	26214
FREQU	25125.24 Hz
SCANS	1024
ACQTM	1.0433 sec
PD	2.0000 sec
PW1	3.03 usec
IRNUC	1H
CTEMP	18.0 c
SLVNT	CDCL3
EXREF	77.01 ppm
BF	1.20 Hz
RGAIN	60

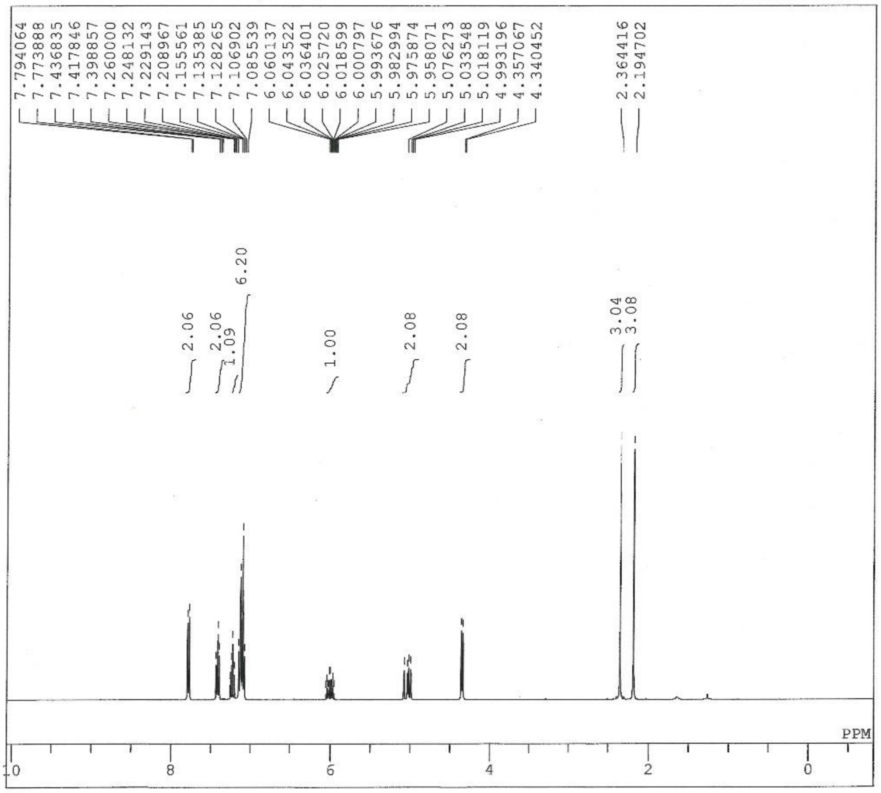

DFILE SF108 20210916-1.als
COMNT Qn-BOC 1H
DATIM 2021-09-16 15:42:12
OBNUC 1H
EXMOD single_pulse.ex2
OBFRQ 399.78 MHz
OBSET 4.62 KHz
OBFIN 5.98 Hz
POINT 16384
FREQU 7773.63 Hz
SCANS 32
ACQTM 2.1076 sec
PD 5.0000 sec
PW1 5.17 usec
IRNUC 1H
CTEMP 19.1 c
SLVNT CDCL3
EXREF 7.26 ppm
BF 0.12 Hz
RGAIN 30

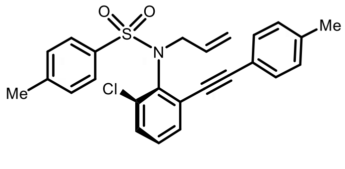

2h

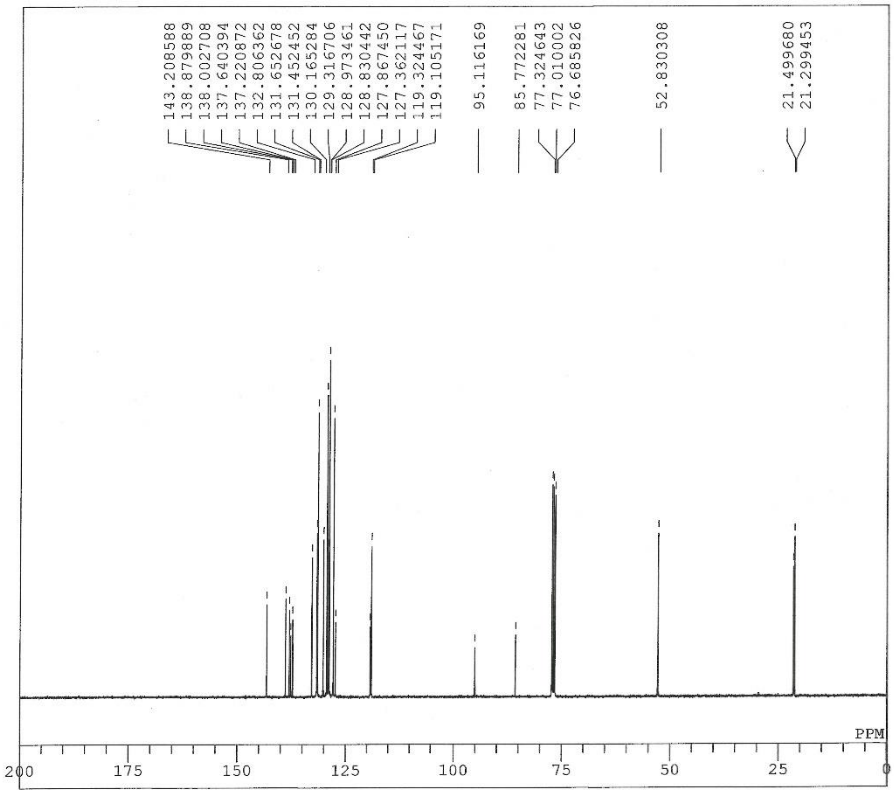

DFILE SF108-13C 20210916-1.als
COMNT
DATIM 2021-09-16 18:27:37
OBNUC 13C
EXMOD single_pulse_dec
OBFRQ 100.53 MHz
OBSET 5.35 KHz
OBFIN 5.86 Hz
POINT 32768
FREQU 31407.03 Hz
SCANS 1024
ACQTM 1.0433 sec
PD 2.0000 sec
PW1 3.03 usec
IRNUC 1H
CTEMP 19.3 c
SLVNT CDCL3
EXREF 77.01 ppm
BF 1.20 Hz
RGAIN 60

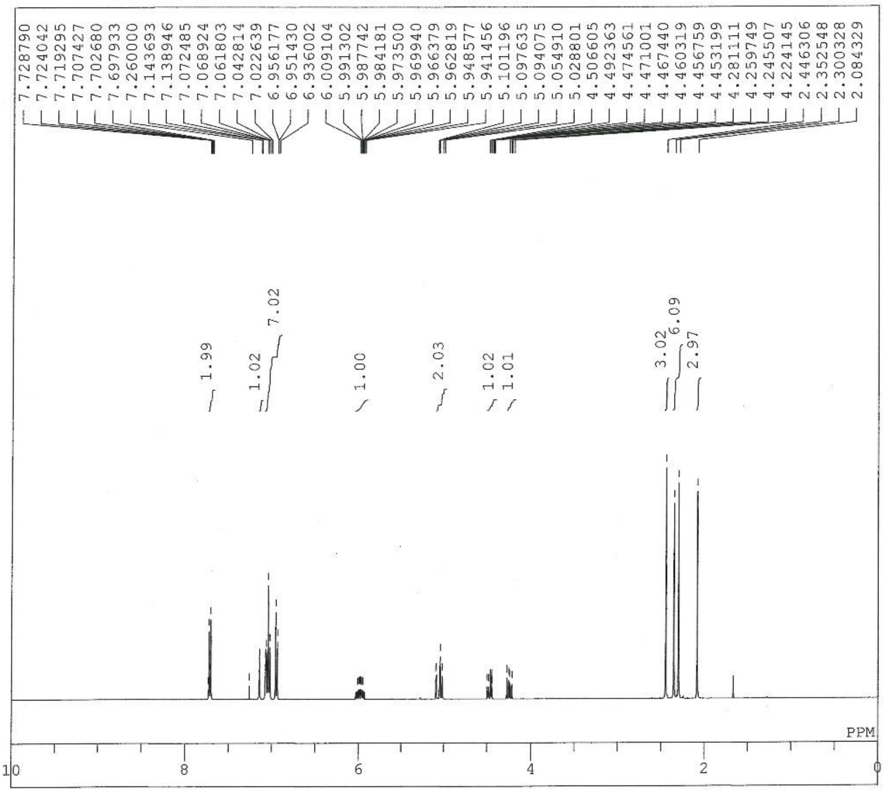

DFILE	SF075 20211008-1.als
COMNT	Qn-BOC 1H
DATIM	2021-10-08 14:09:18
OBNUC	1H
EXMOD	single_pulse.ex2
OBFRQ	399.78 MHz
OBSET	4.62 KHz
OBFIN	5.98 Hz
POINT	16384
FREQU	7773.63 Hz
SCANS	32
ACQTM	2.1076 sec
PD	5.0000 sec
PW1	5.17 usec
IRNUC	1H
CTEMP	18.5 c
SLVNT	CDCL3
EXREF	7.26 ppm
BF	0.12 Hz
RGAIN	26

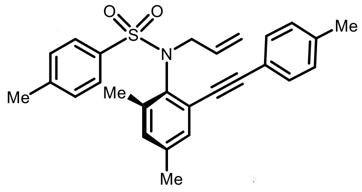

2i

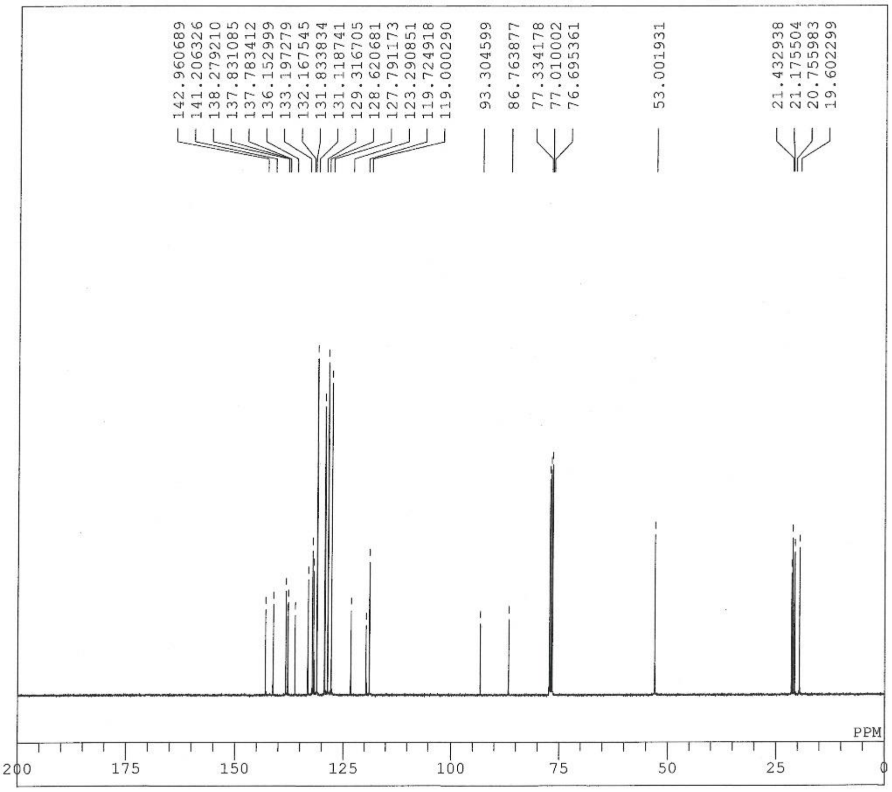

DFILE	SF075-13C 20211004-1.als
COMNT	
DATIM	2021-10-04 18:03:09
OBNUC	13C
EXMOD	single_pulse_dec
OBFRQ	100.53 MHz
OBSET	5.35 KHz
OBFIN	5.86 Hz
POINT	32768
FREQU	31407.03 Hz
SCANS	1024
ACQTM	1.0433 sec
PD	2.0000 sec
PW1	3.03 usec
IRNUC	1H
CTEMP	18.5 c
SLVNT	CDCL3
EXREF	77.01 ppm
BF	1.20 Hz
RGAIN	60

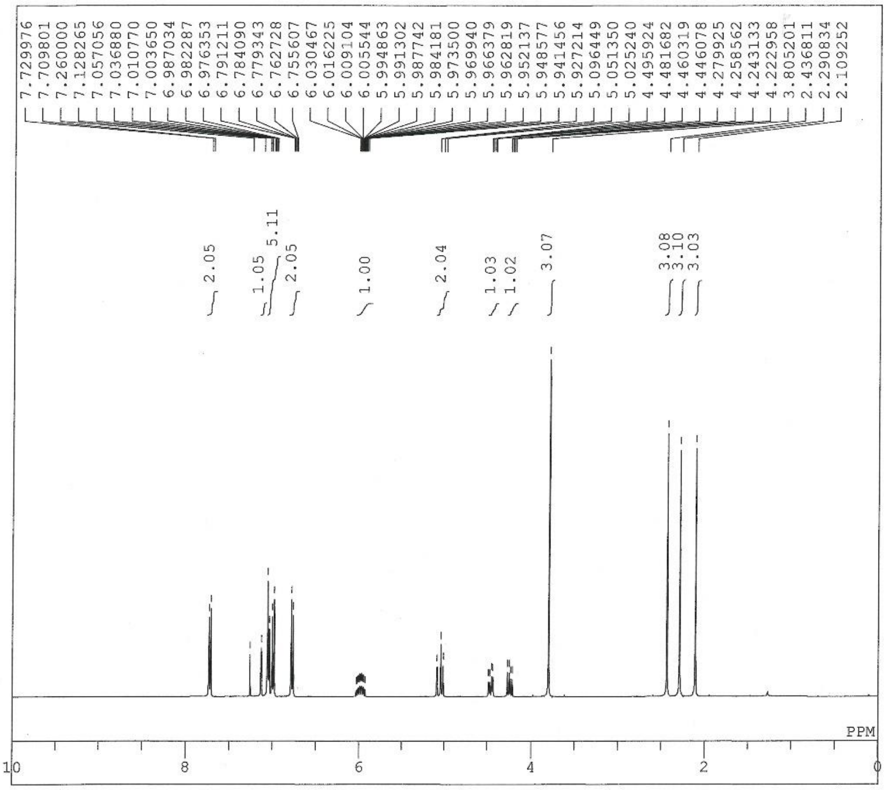

DFILE	TF012 20210930-1.als
COMNT	Qn-BOC 1H
DATIM	2021-09-30 10:08:27
OBNUC	1H
EXMOD	single_pulse.ex2
OBFRQ	399.78 MHz
OBSET	4.62 KHz
OBFIN	5.98 Hz
POINT	16384
FREQU	7773.63 Hz
SCANS	32
ACQTM	2.1076 sec
PD	5.0000 sec
PW1	5.17 usec
IRNUC	1H
CTEMP	19.0 c
SLVNT	CDCL3
EXREF	7.26 ppm
BF	0.12 Hz
RGAIN	22

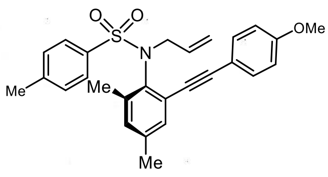

2j

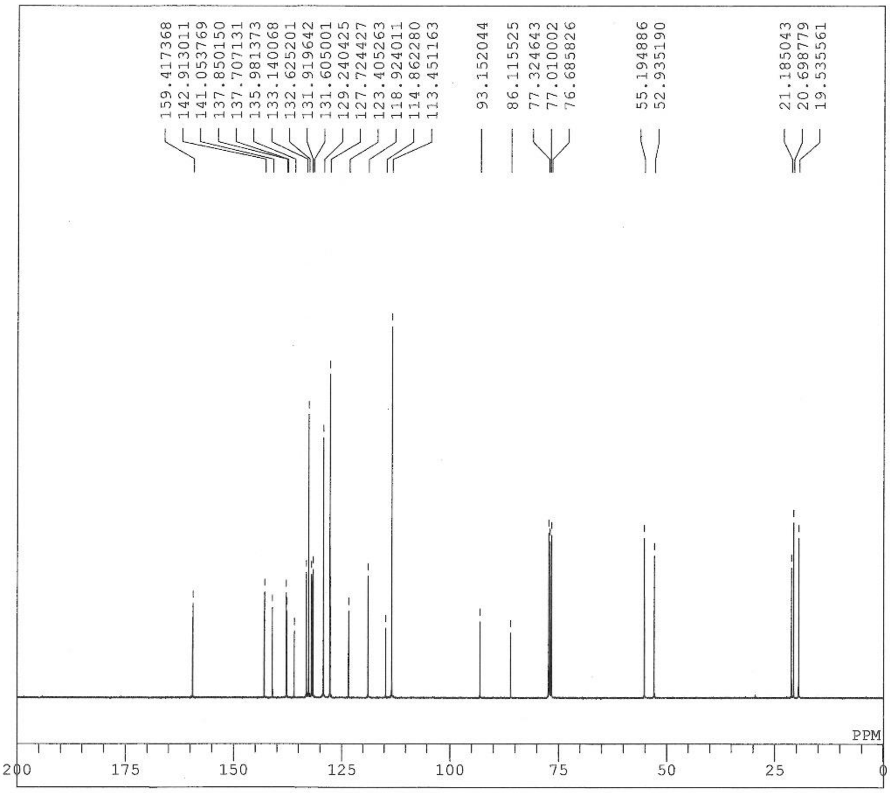

DFILE	TF012-13C 20211002-1.als
COMNT	
DATIM	2021-10-02 10:52:49
OBNUC	13C
EXMOD	single_pulse_dec
OBFRQ	100.53 MHz
OBSET	5.35 KHz
OBFIN	5.86 Hz
POINT	26214
FREQU	25125.24 Hz
SCANS	1024
ACQTM	1.0433 sec
PD	2.0000 sec
PW1	3.03 usec
IRNUC	1H
CTEMP	18.5 c
SLVNT	CDCL3
EXREF	77.01 ppm
BF	1.20 Hz
RGAIN	60

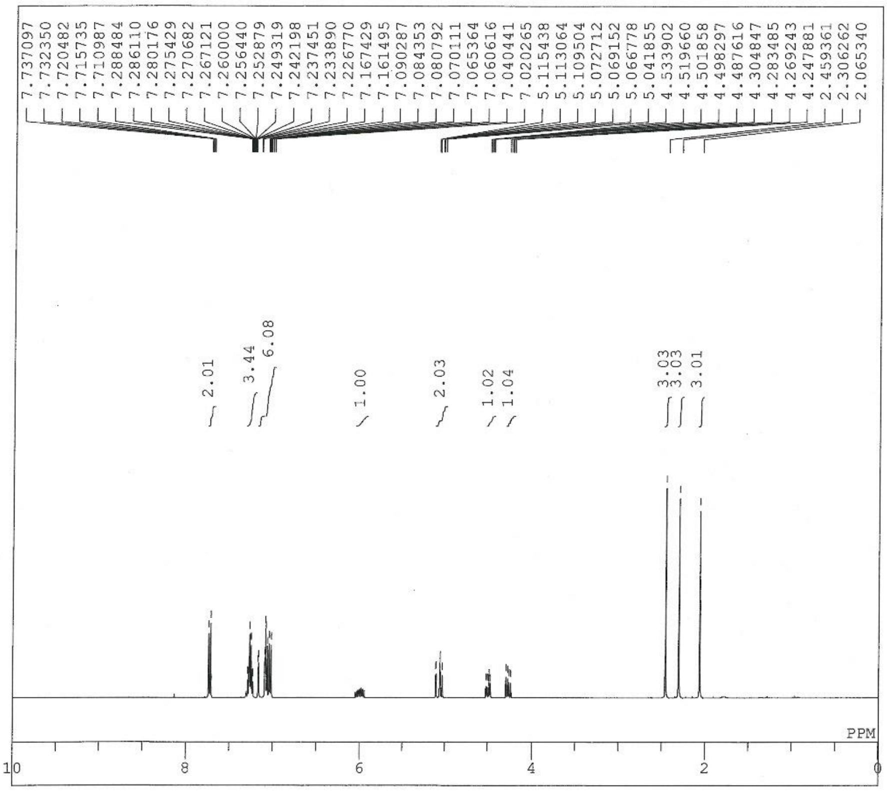

DFILE YO021 20210927-1.als
COMNT Qn-BOC 1H
DATIM 2021-09-27 11:10:30
OBNUC 1H
EXMOD single_pulse.ex2
OBFRQ 399.78 MHz
OBSET 4.62 KHz
OBFIN 5.98 Hz
POINT 16384
FREQU 7773.63 Hz
SCANS 32
ACQTM 2.1076 sec
PD 5.0000 sec
PW1 5.17 usec
IRNUC 1H
CTEMP 18.7 c
SLVNT CDCL3
EXREF 7.26 ppm
BF 0.12 Hz
RGAIN 20

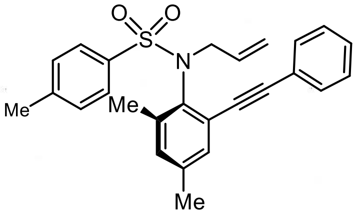

2k

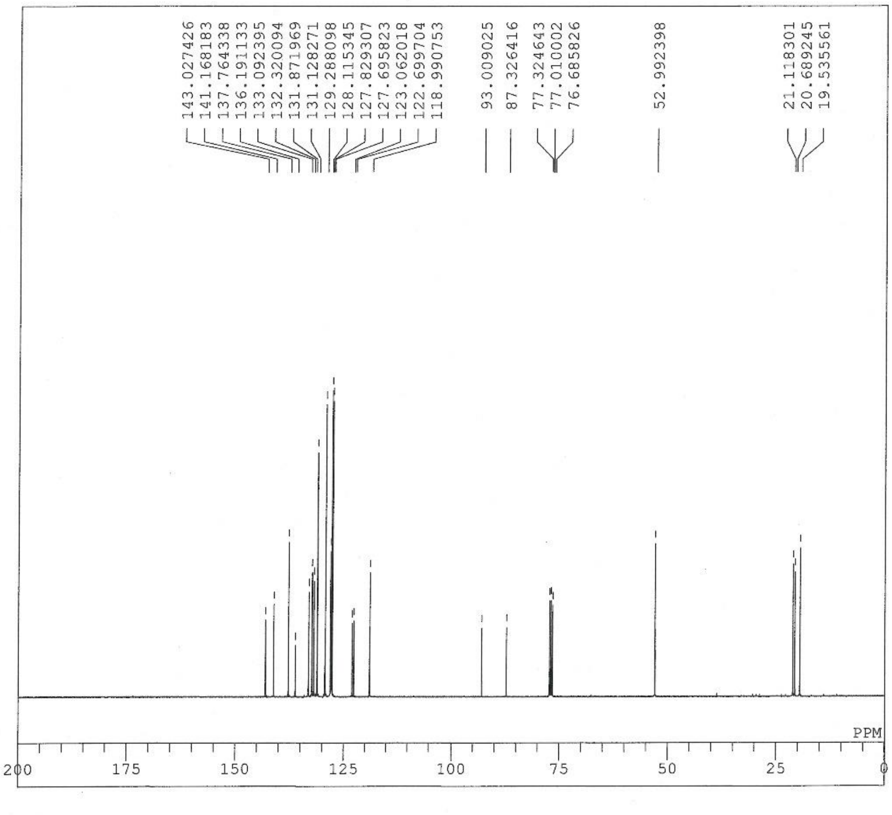

DFILE YO021-13C 20210924-1.als
COMNT
DATIM 2021-09-24 18:08:11
OBNUC 13C
EXMOD single_pulse_dec
OBFRQ 100.53 MHz
OBSET 5.35 KHz
OBFIN 5.86 Hz
POINT 32768
FREQU 31407.03 Hz
SCANS 1024
ACQTM 1.0433 sec
PD 2.0000 sec
PW1 3.03 usec
IRNUC 1H
CTEMP 19.5 c
SLVNT CDCL3
EXREF 77.01 ppm
BF 1.20 Hz
RGAIN 60

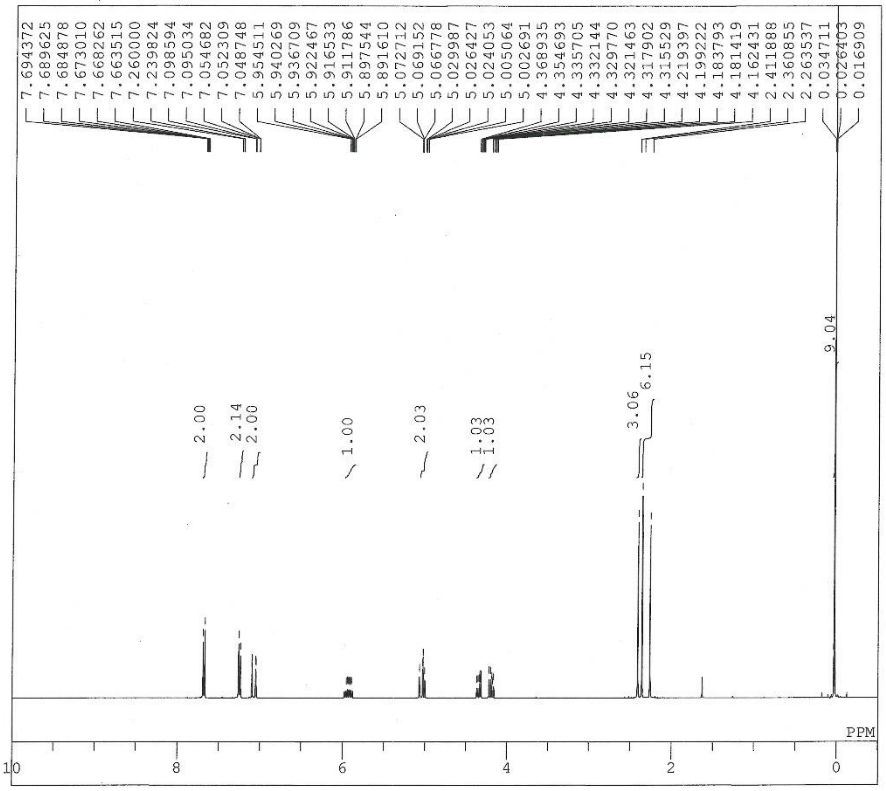

DFILE	TT064 20211123-1.als
COMNT	Qn-BOC 1H
DATIM	2021-11-23 14:49:48
OBNUC	1H
EXMOD	single_pulse.ex2
OBFRQ	399.78 MHz
OBSET	4.62 KHz
OBFIN	5.98 Hz
POINT	13107
FREQU	6218.81 Hz
SCANS	32
ACQTM	2.1076 sec
PD	5.0000 sec
PW1	5.80 usec
IRNUC	1H
CTEMP	18.8 c
SLVNT	CDCL3
EXREF	7.26 ppm
BF	0.12 Hz
RGAIN	30

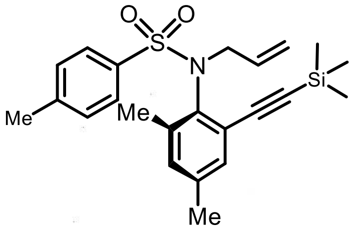

2l

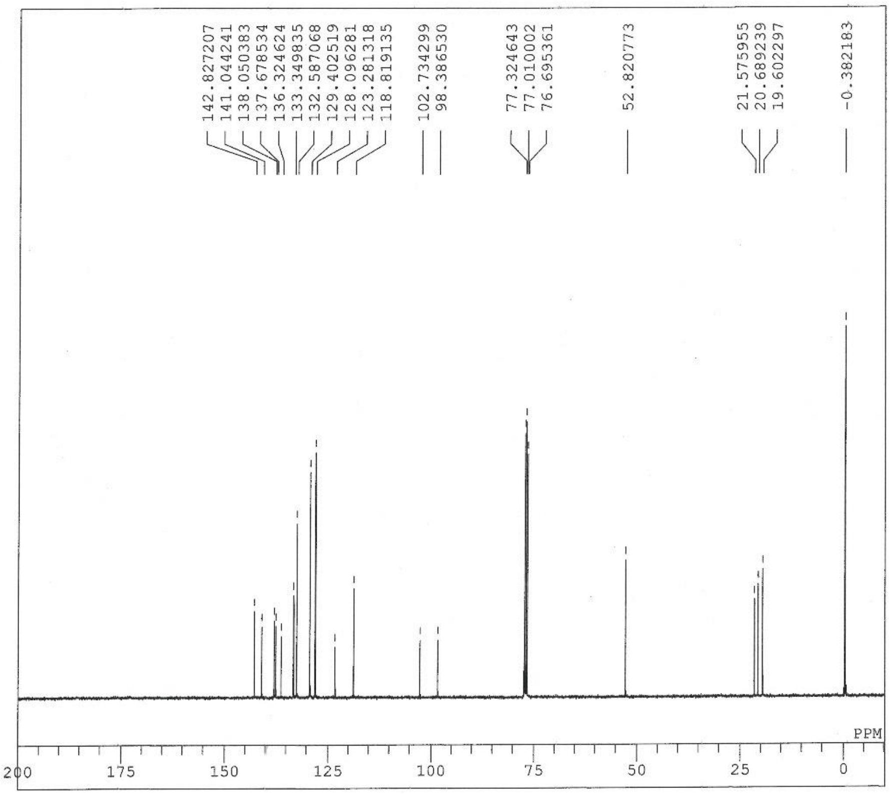

DFILE	TT064-13C 20211123-1.als
COMNT	
DATIM	2021-11-23 18:01:60
OBNUC	13C
EXMOD	single_pulse_dec
OBFRQ	100.53 MHz
OBSET	5.35 KHz
OBFIN	5.86 Hz
POINT	32768
FREQU	31407.03 Hz
SCANS	1024
ACQTM	1.0433 sec
PD	2.0000 sec
PW1	3.23 usec
IRNUC	1H
CTEMP	19.1 c
SLVNT	CDCL3
EXREF	77.01 ppm
BF	1.20 Hz
RGAIN	60

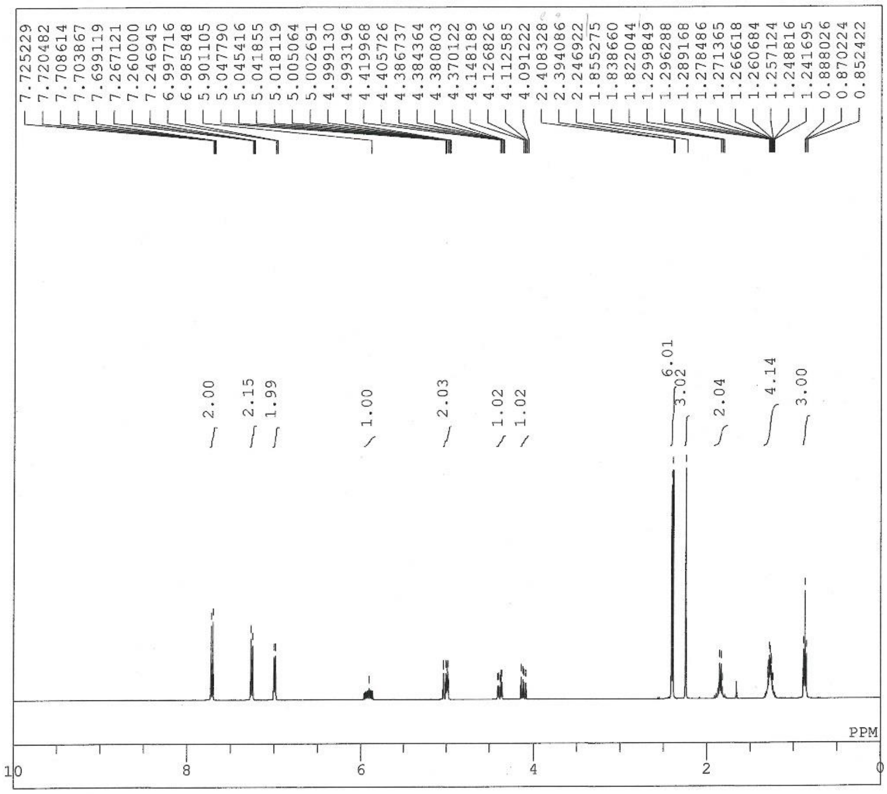

DFILE SF138 20211121-1.jdf
COMNT Qn-BOC 1H
DATIM 2021-11-21 17:14:58
OBNUC 1H
EXMOD single_pulse.ex2
OBFRQ 399.78 MHz
OBSET 4.62 KHz
OBFIN 5.98 Hz
POINT 16384
FREQU 7773.63 Hz
SCANS 32
ACQTM 2.1076 sec
PD 5.0000 sec
PW1 5.80 usec
IRNUC 1H
CTEMP 18.5 c
SLVNT CDCL3
EXREF 7.26 ppm
BF 0.12 Hz
RGAIN 26

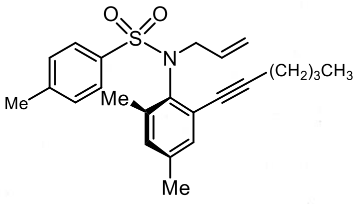

2m

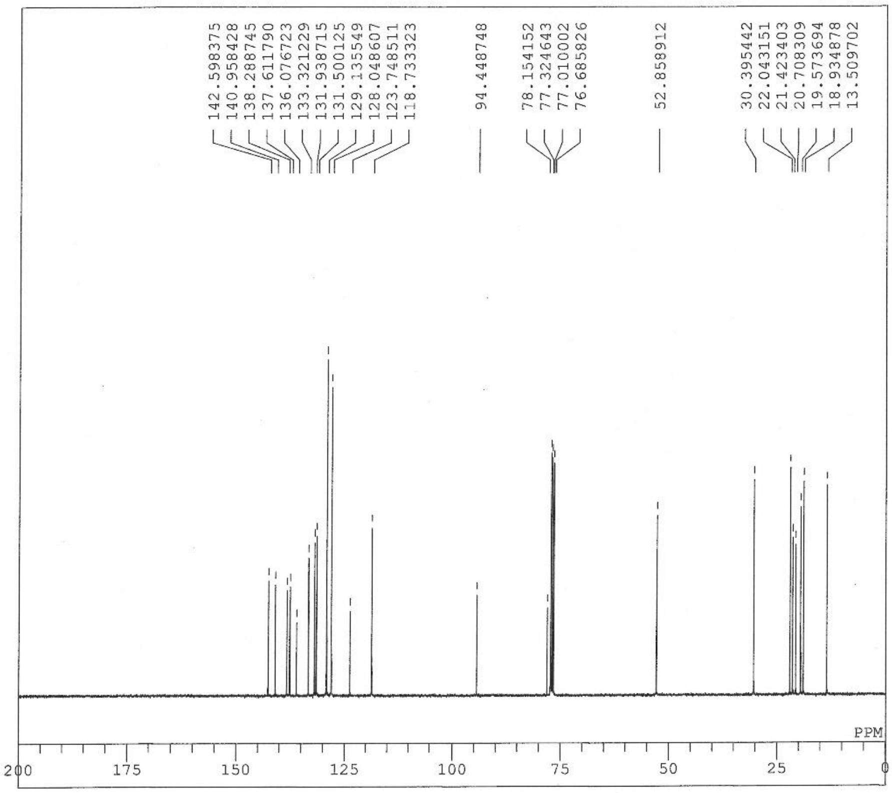

DFILE SF138-13C 20211121-1.jdf
COMNT
DATIM 2021-11-21 17:01:13
OBNUC 13C
EXMOD single_pulse_dec
OBFRQ 100.53 MHz
OBSET 5.35 KHz
OBFIN 5.86 Hz
POINT 32768
FREQU 31407.03 Hz
SCANS 1024
ACQTM 1.0433 sec
PD 2.0000 sec
PW1 3.23 usec
IRNUC 1H
CTEMP 19.0 c
SLVNT CDCL3
EXREF 77.01 ppm
BF 1.20 Hz
RGAIN 60

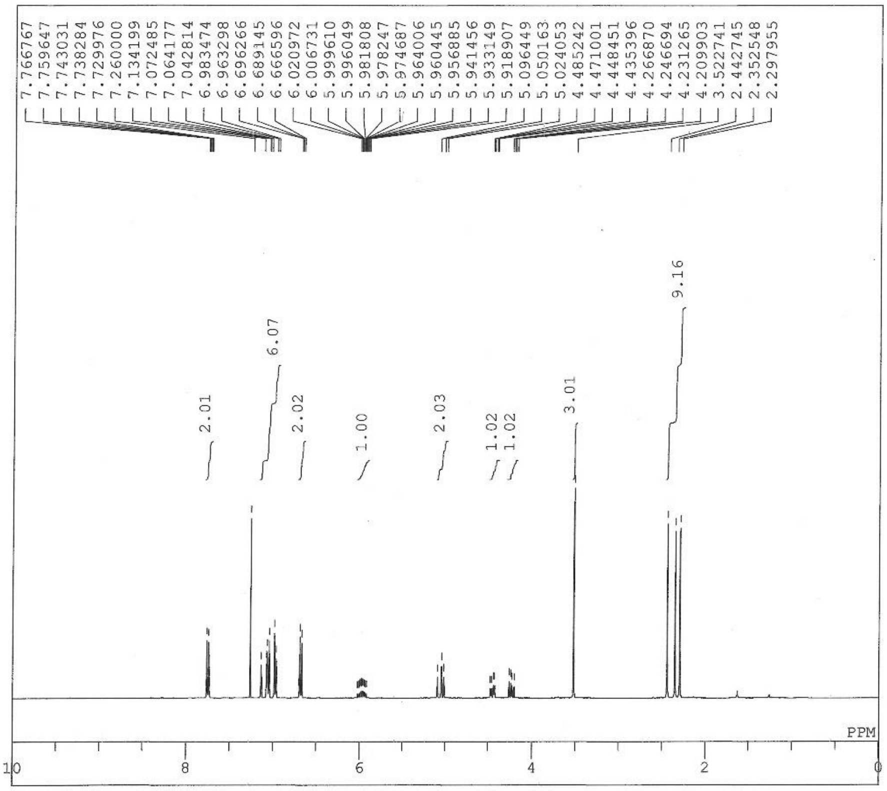

DFILE TF008 20211203 sin-1.als
COMNT Qn-BOC 1H
DATIM 2021-12-03 15:28:44
OBNUC 1H
EXMOD single_pulse.ex2
OBFRQ 399.78 MHz
OBSET 4.62 KHz
OBFIN 5.98 Hz
POINT 13107
FREQU 6218.81 Hz
SCANS 32
ACQTM 2.1076 sec
PD 5.0000 sec
PW1 5.80 usec
IRNUC 1H
CTEMP 18.6 c
SLVNT CDCL3
EXREF 7.26 ppm
BF 0.12 Hz
RGAIN 30

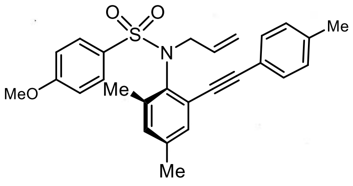

2n

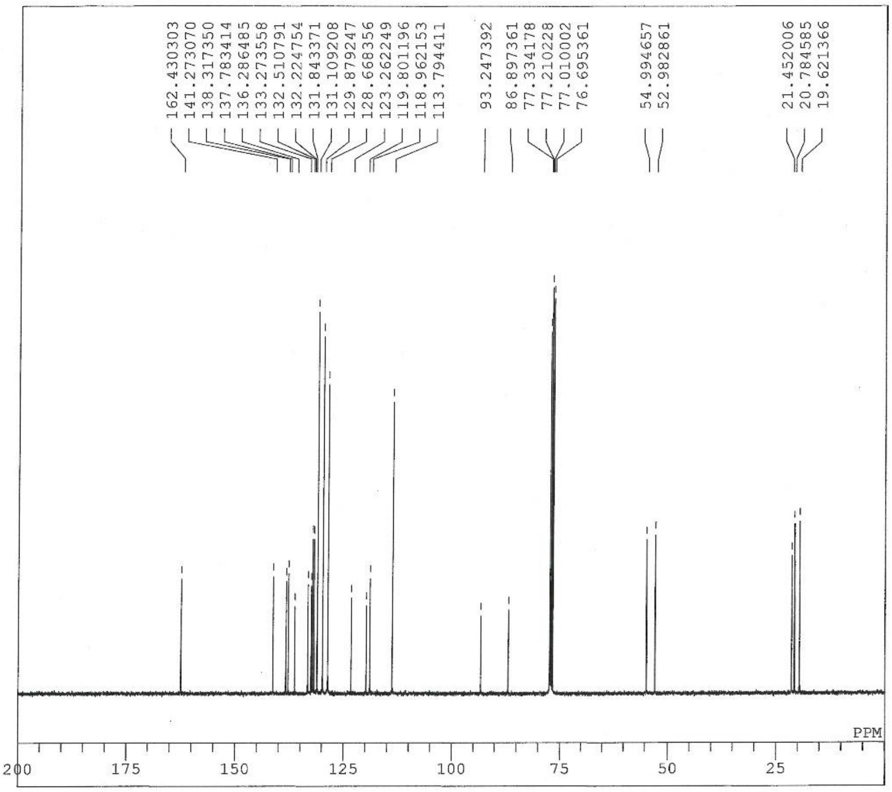

DFILE TF008 13C 20211204-1.als
COMNT
DATIM 2021-12-04 10:39:27
OBNUC 13C
EXMOD single_pulse_dec
OBFRQ 100.53 MHz
OBSET 5.35 KHz
OBFIN 5.86 Hz
POINT 32768
FREQU 31407.03 Hz
SCANS 1024
ACQTM 1.0433 sec
PD 2.0000 sec
PW1 3.23 usec
IRNUC 1H
CTEMP 18.4 c
SLVNT CDCL3
EXREF 77.01 ppm
BF 1.20 Hz
RGAIN 60

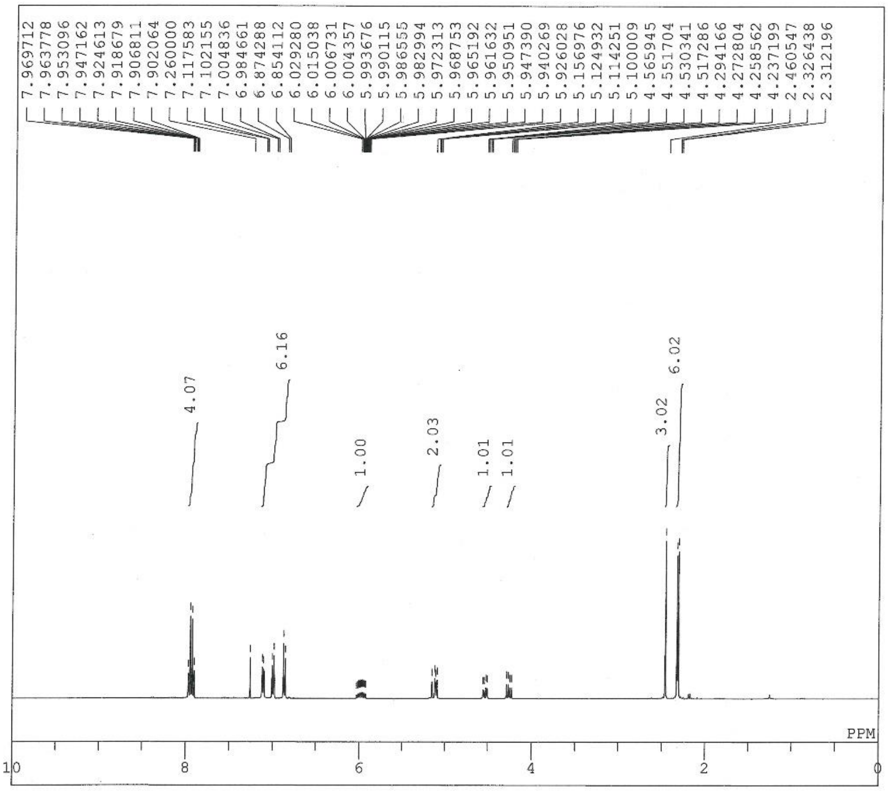

DFILE TT011 20211204-1.als
COMNT Qn-BOC 1H
DATIM 2021-12-04 10:51:14
OBNUC 1H
EXMOD single_pulse.ex2
OBFRQ 399.78 MHz
OBSET 4.62 KHz
OBFIN 5.98 Hz
POINT 13107
FREQU 6218.81 Hz
SCANS 32
ACQTM 2.1076 sec
PD 5.0000 sec
PW1 5.80 usec
IRNUC 1H
CTEMP 18.0 c
SLVNT CDCL3
EXREF 7.26 ppm
BF 0.12 Hz
RGAIN 30

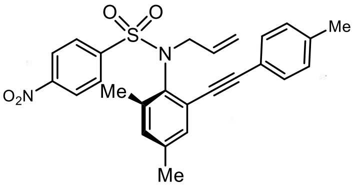

2o

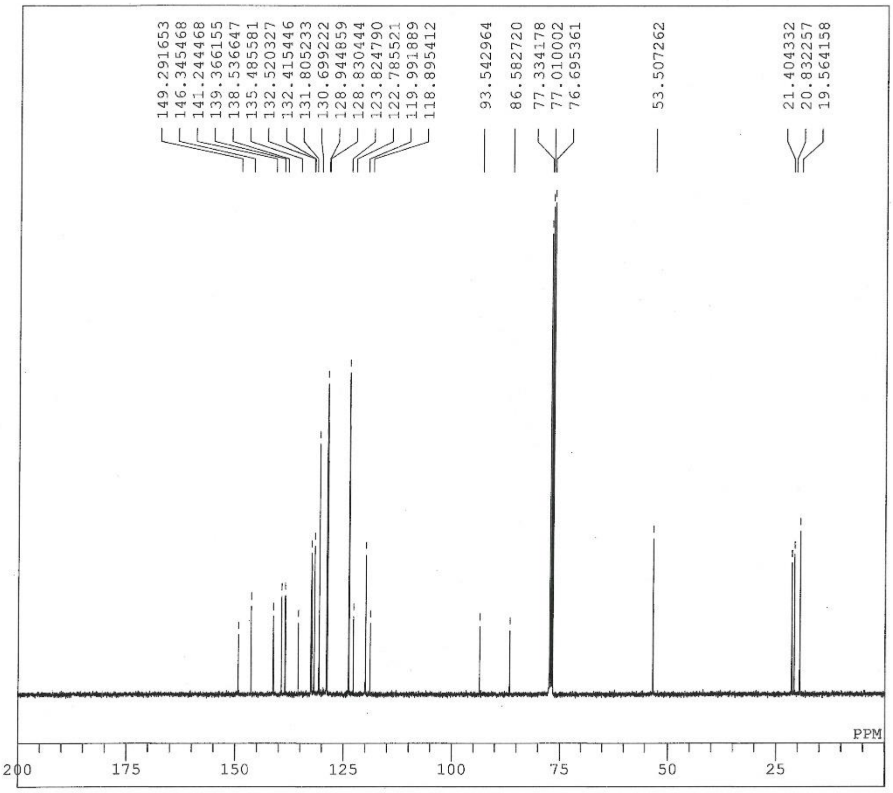

DFILE TT011 13C 20211203-1.als
COMNT
DATIM 2021-12-03 09:32:53
OBNUC 13C
EXMOD single_pulse_dec
OBFRQ 100.53 MHz
OBSET 5.35 KHz
OBFIN 5.86 Hz
POINT 26214
FREQU 25125.24 Hz
SCANS 1024
ACQTM 1.0433 sec
PD 2.0000 sec
PW1 3.23 usec
IRNUC 1H
CTEMP 17.6 c
SLVNT CDCL3
EXREF 77.01 ppm
BF 1.20 Hz
RGAIN 60

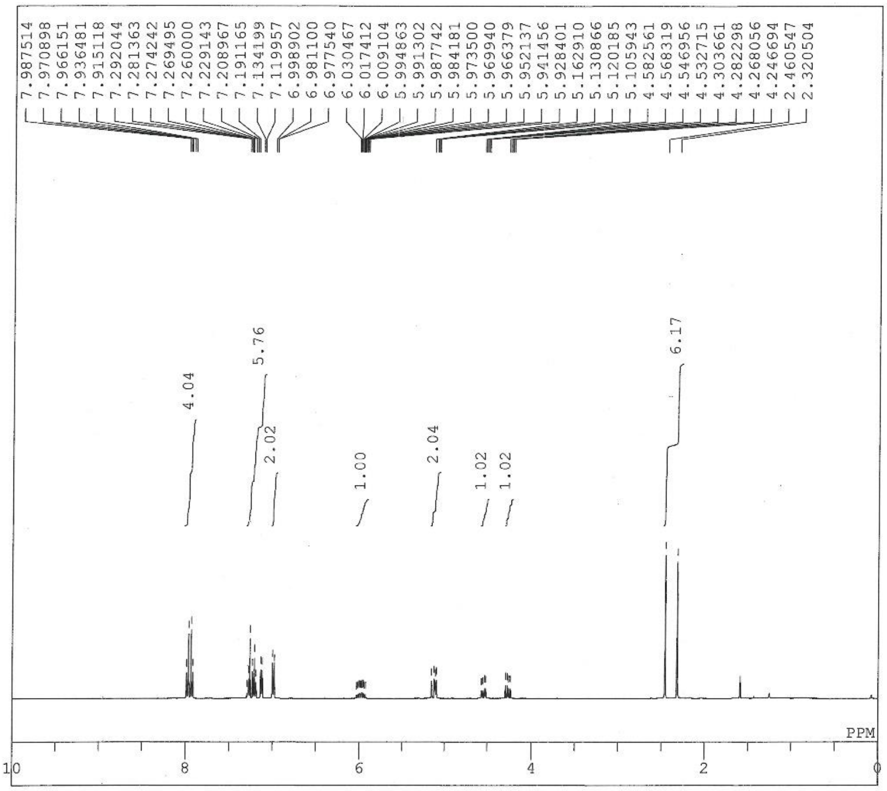

DFILE RK020 20211129-1.als
COMNT Qn-BOC 1H
DATIM 2021-11-29 12:21:25
OBNUC 1H
EXMOD single_pulse.ex2
OBFRQ 399.78 MHz
OBSET 4.62 KHz
OBFIN 5.98 Hz
POINT 13107
FREQU 6218.81 Hz
SCANS 32
ACQTM 2.1076 sec
PD 5.0000 sec
PW1 5.80 usec
IRNUC 1H
CTEMP 17.9 c
SLVNT CDCL3
EXREF 7.26 ppm
BF 0.12 Hz
RGAIN 30

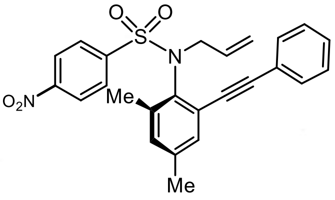

2p

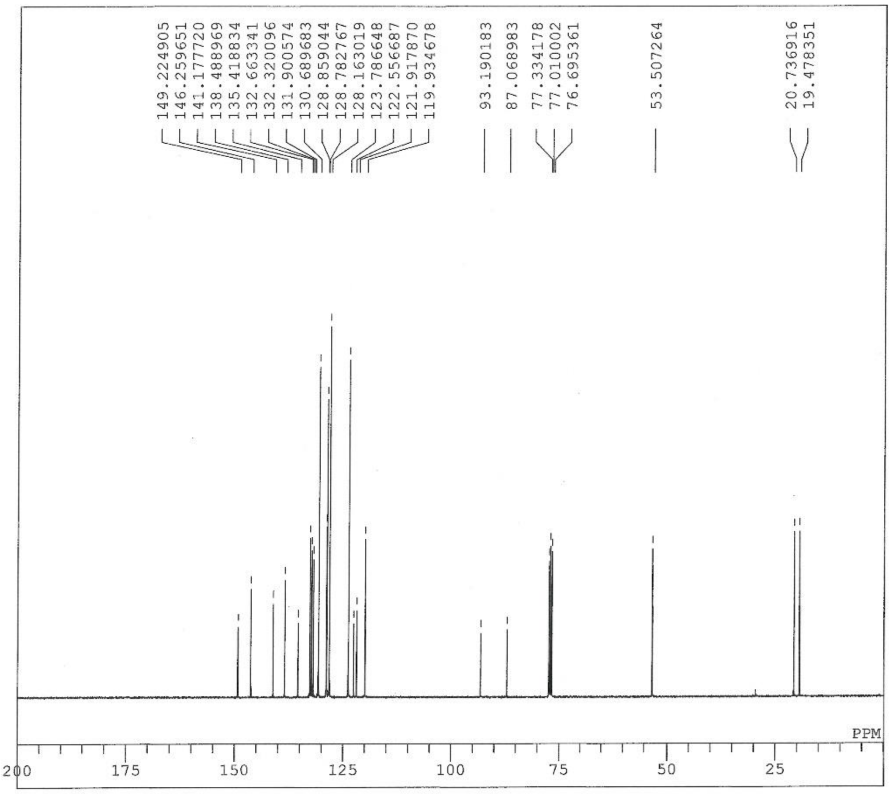

DFILE SF094 13C 20211202-1.als
COMNT
DATIM 2021-12-02 09:37:24
OBNUC 13C
EXMOD single_pulse_dec
OBFRQ 100.53 MHz
OBSET 5.35 KHz
OBFIN 5.86 Hz
POINT 26214
FREQU 25125.24 Hz
SCANS 1024
ACQTM 1.0433 sec
PD 2.0000 sec
PW1 3.23 usec
IRNUC 1H
CTEMP 17.9 c
SLVNT CDCL3
EXREF 77.01 ppm
BF 1.20 Hz
RGAIN 60

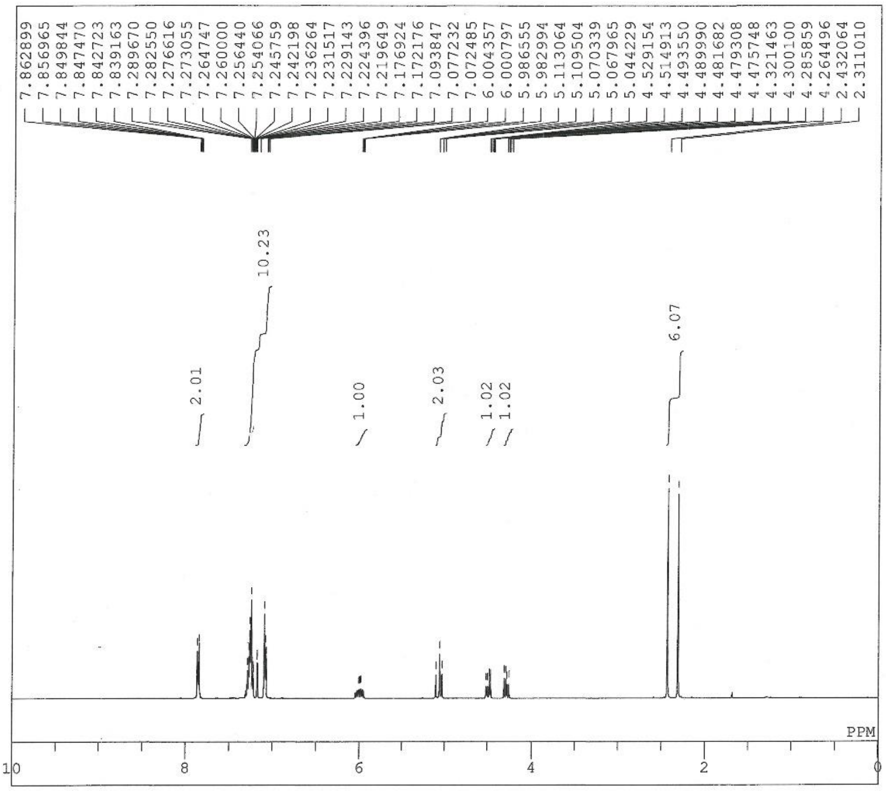

DFILE SF122 20210927-1.als
COMNT Qn-BOC 1H
DATIM 2021-09-27 11:33:23
OBNUC 1H
EXMOD single_pulse.ex2
OBFRQ 399.78 MHz
OBSET 4.62 KHz
OBFIN 5.98 Hz
POINT 13107
FREQU 6218.81 Hz
SCANS 32
ACQTM 2.1076 sec
PD 5.0000 sec
PW1 5.17 usec
IRNUC 1H
CTEMP 18.9 c
SLVNT CDCL3
EXREF 7.26 ppm
BF 0.12 Hz
RGAIN 22

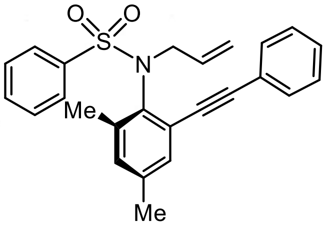

2q

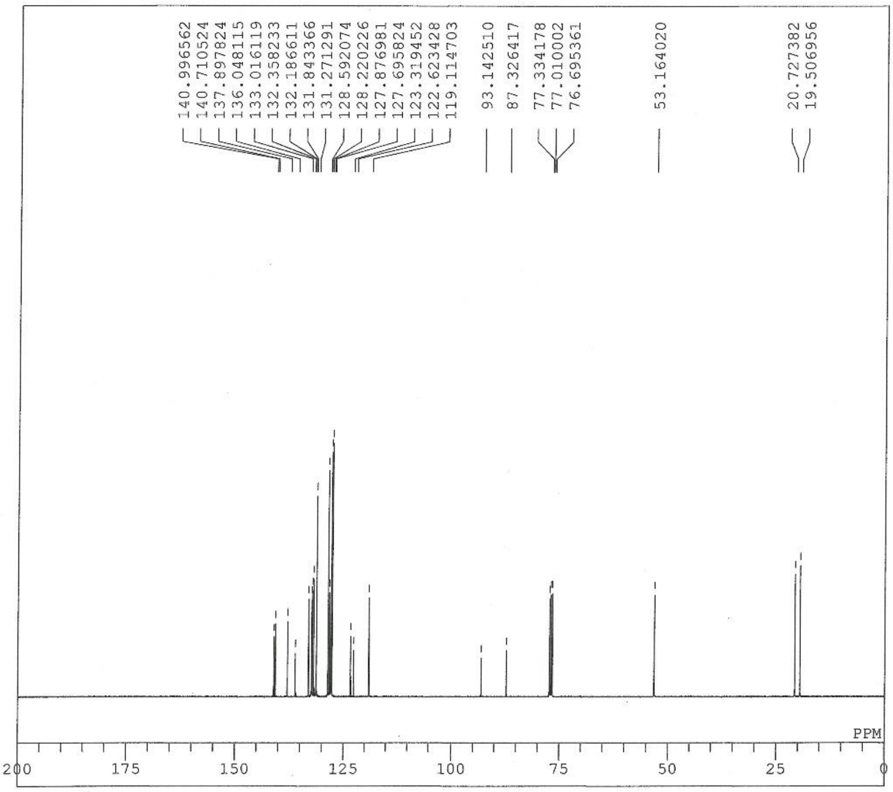

DFILE SF122-13C 20210928-1.als
COMNT
DATIM 2021-09-28 09:45:01
OBNUC 13C
EXMOD single_pulse_dec
OBFRQ 100.53 MHz
OBSET 5.35 KHz
OBFIN 5.86 Hz
POINT 26214
FREQU 25125.24 Hz
SCANS 1024
ACQTM 1.0433 sec
PD 2.0000 sec
PW1 3.03 usec
IRNUC 1H
CTEMP 19.2 c
SLVNT CDCL3
EXREF 77.01 ppm
BF 1.20 Hz
RGAIN 60

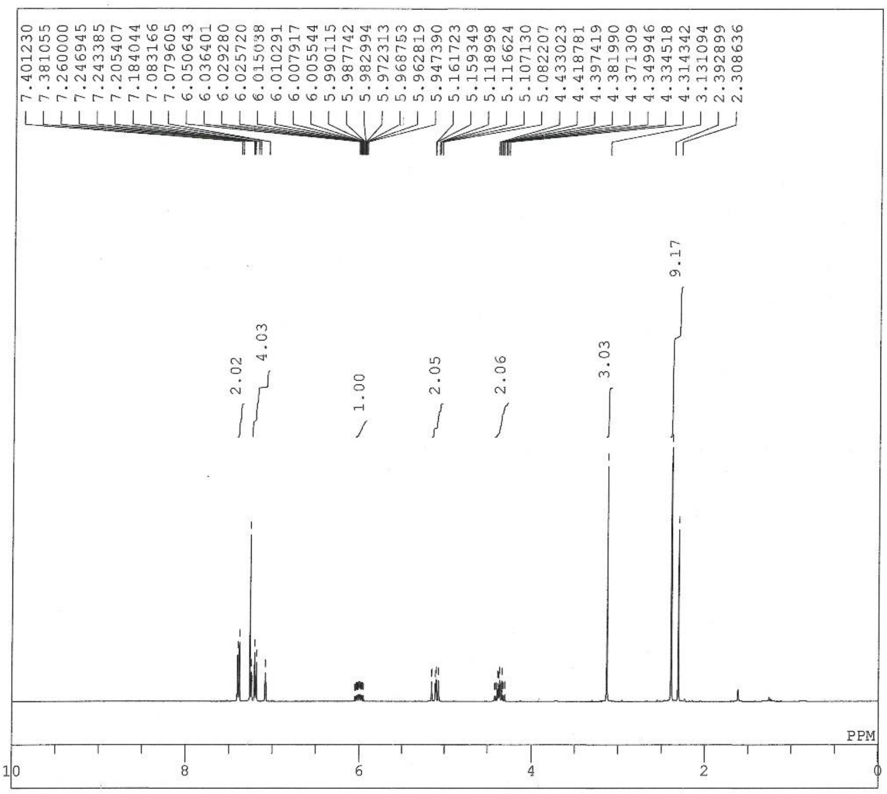

DFILE SF094 20211129-1.als
COMNT Qn-BOC 1H
DATIM 2021-11-29 12:09:12
OBNUC 1H
EXMOD single_pulse.ex2
OBFRQ 399.78 MHz
OBSET 4.62 KHz
OBFIN 5.98 Hz
POINT 13107
FREQU 6218.81 Hz
SCANS 32
ACQTM 2.1076 sec
PD 5.0000 sec
PW1 5.80 usec
IRNUC 1H
CTEMP 17.6 c
SLVNT CDCL3
EXREF 7.26 ppm
BF 0.12 Hz
RGAIN 30

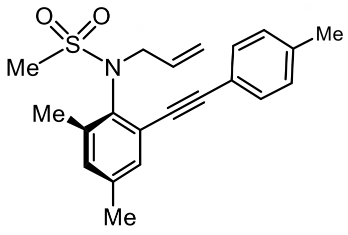

2r

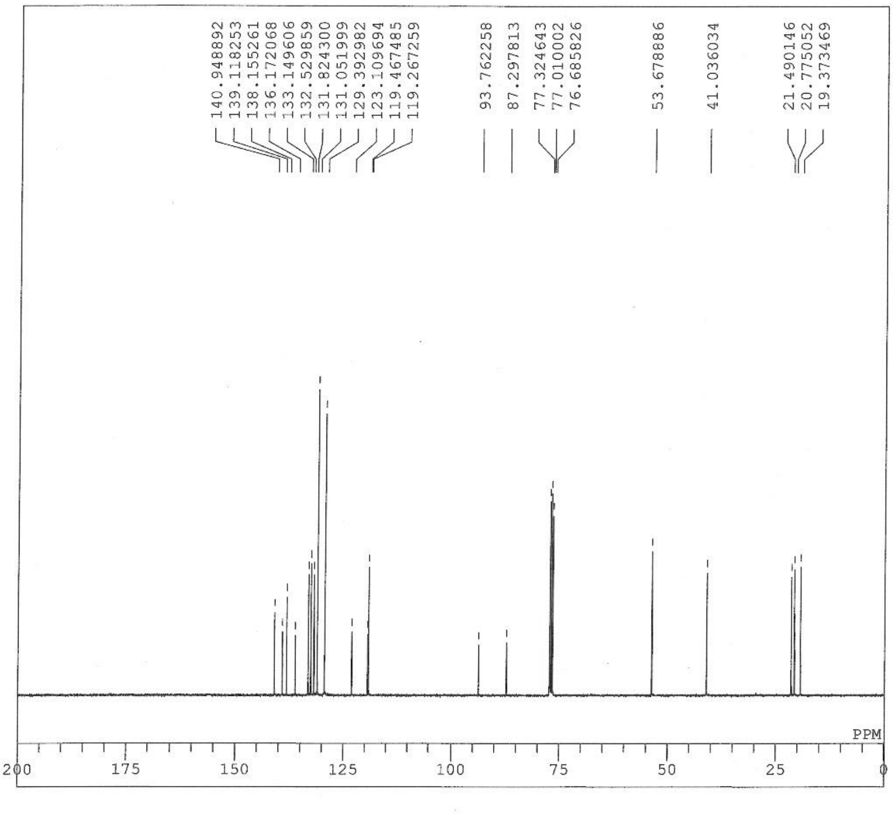

DFILE RK020 13C 20211201-1.als
COMNT
DATIM 2021-12-01 19:42:38
OBNUC 13C
EXMOD single_pulse_dec
OBFRQ 100.53 MHz
OBSET 5.35 KHz
OBFIN 5.86 Hz
POINT 26214
FREQU 25125.24 Hz
SCANS 1024
ACQTM 1.0433 sec
PD 2.0000 sec
PW1 3.23 usec
IRNUC 1H
CTEMP 18.8 c
SLVNT CDCL3
EXREF 77.01 ppm
BF 1.20 Hz
RGAIN 60

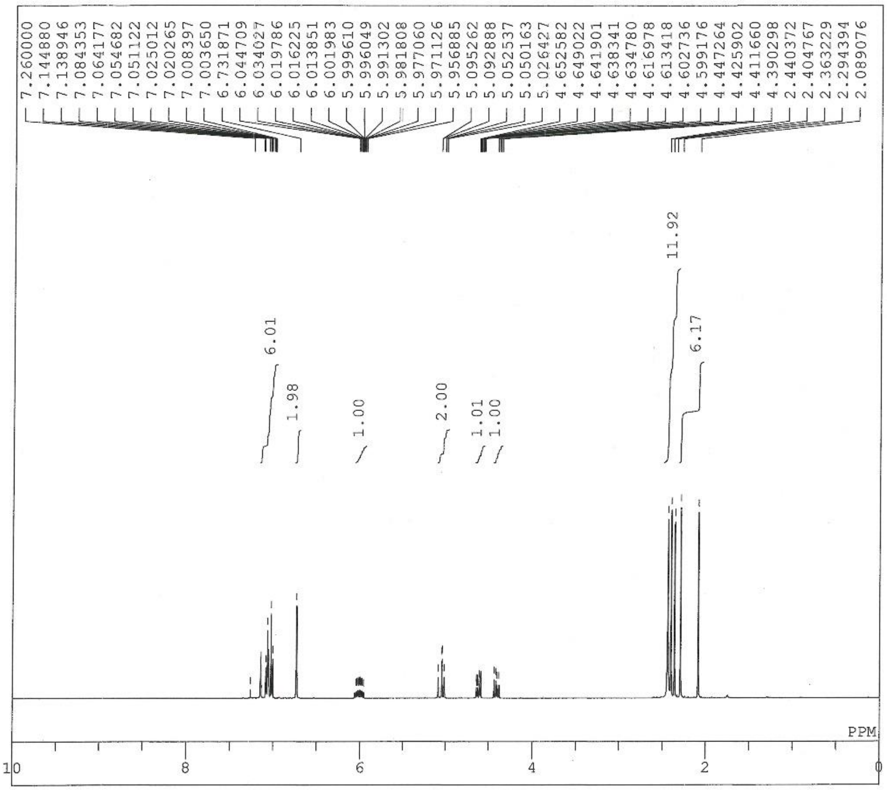


```
DFILE   TT020 20210922-1.als
COMNT   Qn-BOC 1H
DATIM   2021-09-22 17:22:21
OBNUC   1H
EXMOD   single_pulse.ex2
OBFRQ       399.78 MHz
OBSET         4.62 KHz
OBFIN         5.98 Hz
POINT        13107
FREQU      6218.81 Hz
SCANS           32
ACQTM       2.1076 sec
PD          5.0000 sec
PW1           5.17 usec
IRNUC   1H
CTEMP         20.4 c
SLVNT   CDCL3
EXREF         7.26 ppm
BF            0.12 Hz
RGAIN           30
```

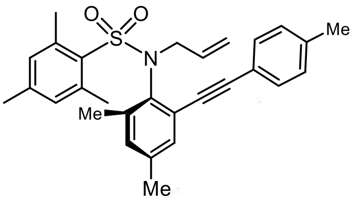

2s

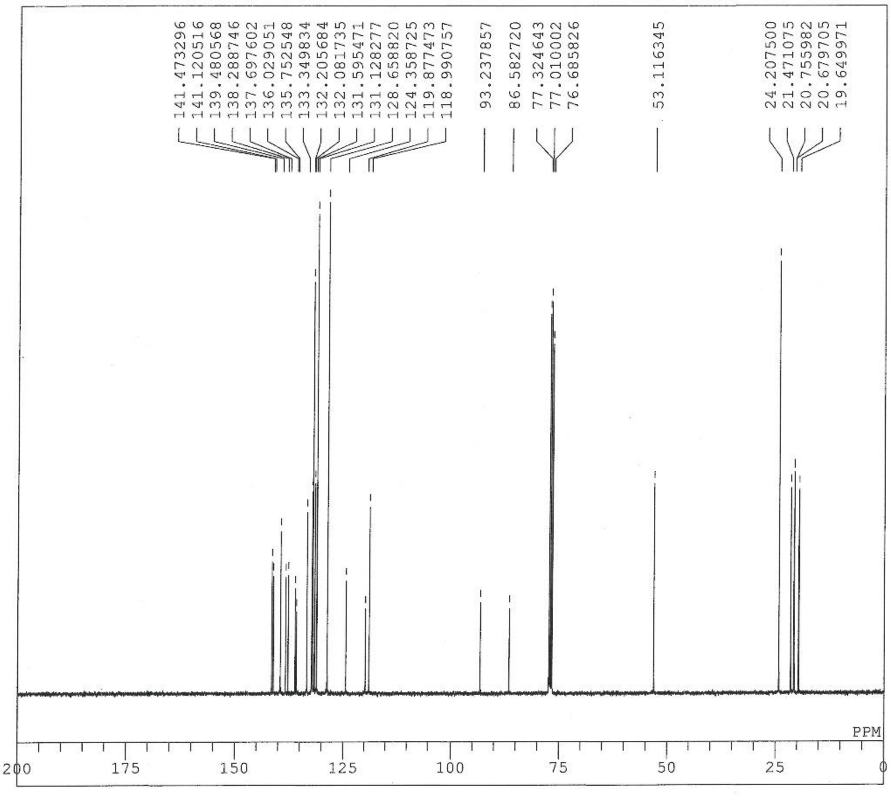


```
DFILE   TT020 13C 20211217-1.als
COMNT
DATIM   2021-12-17 09:43:06
OBNUC   13C
EXMOD   single_pulse_dec
OBFRQ       100.53 MHz
OBSET         5.35 KHz
OBFIN         5.86 Hz
POINT        32768
FREQU     31407.03 Hz
SCANS         1024
ACQTM       1.0433 sec
PD          2.0000 sec
PW1           3.23 usec
IRNUC   1H
CTEMP         18.0 c
SLVNT   CDCL3
EXREF        77.01 ppm
BF            1.20 Hz
RGAIN           60
```

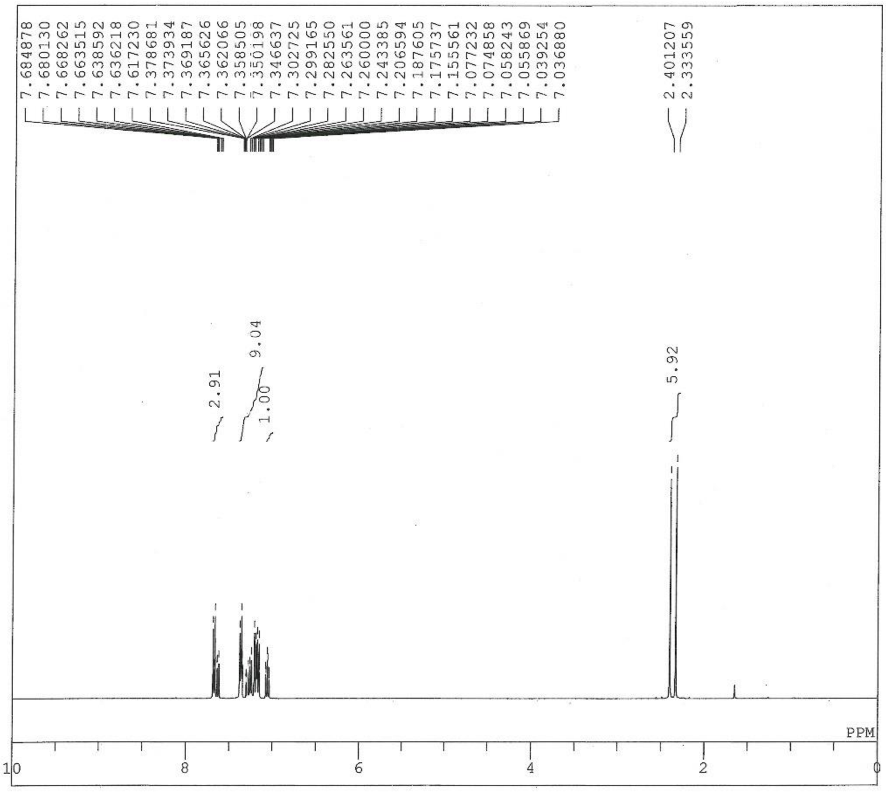

DFILE YO024-2 20211209-1.als
COMNT Qn-BOC 1H
DATIM 2021-12-09 15:50:05
OBNUC 1H
EXMOD single_pulse.ex2
OBFRQ 399.78 MHz
OBSET 4.62 KHz
OBFIN 5.98 Hz
POINT 16384
FREQU 7773.63 Hz
SCANS 32
ACQTM 2.1076 sec
PD 5.0000 sec
PW1 5.80 usec
IRNUC 1H
CTEMP 18.8 c
SLVNT CDCL3
EXREF 7.26 ppm
BF 0.12 Hz
RGAIN 30

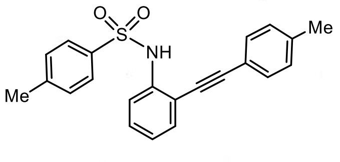

6

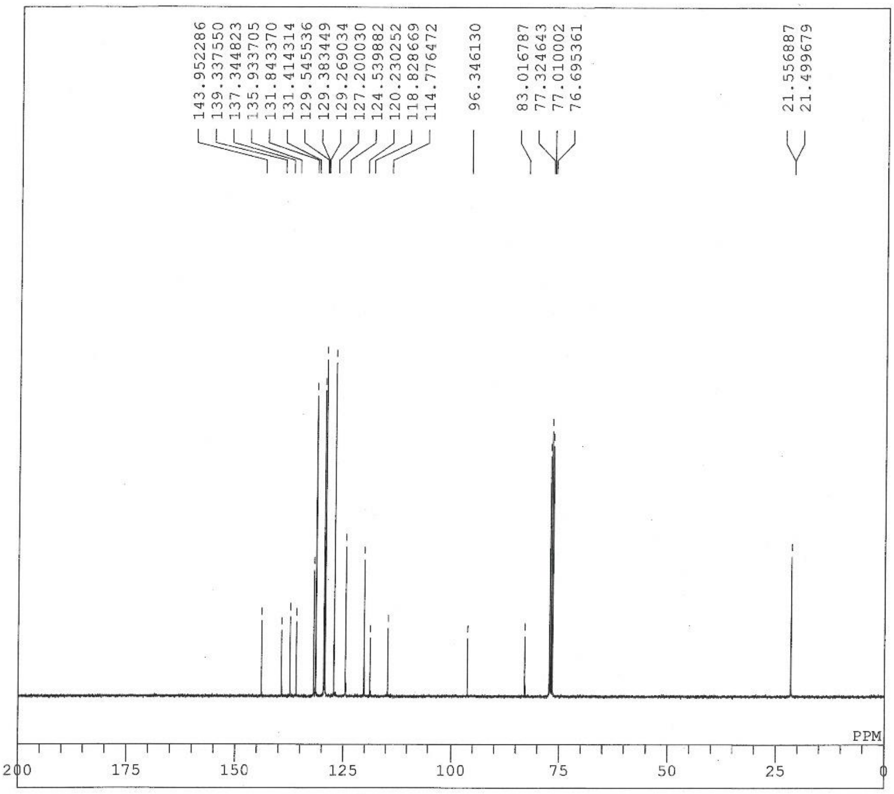

DFILE YO024-13C 20211209-1.jdf
COMNT
DATIM 2021-12-09 18:15:52
OBNUC 13C
EXMOD single_pulse_dec
OBFRQ 100.53 MHz
OBSET 5.35 KHz
OBFIN 5.86 Hz
POINT 32768
FREQU 31407.03 Hz
SCANS 1024
ACQTM 1.0433 sec
PD 2.0000 sec
PW1 3.23 usec
IRNUC 1H
CTEMP 18.7 c
SLVNT CDCL3
EXREF 77.01 ppm
BF 1.20 Hz
RGAIN 60

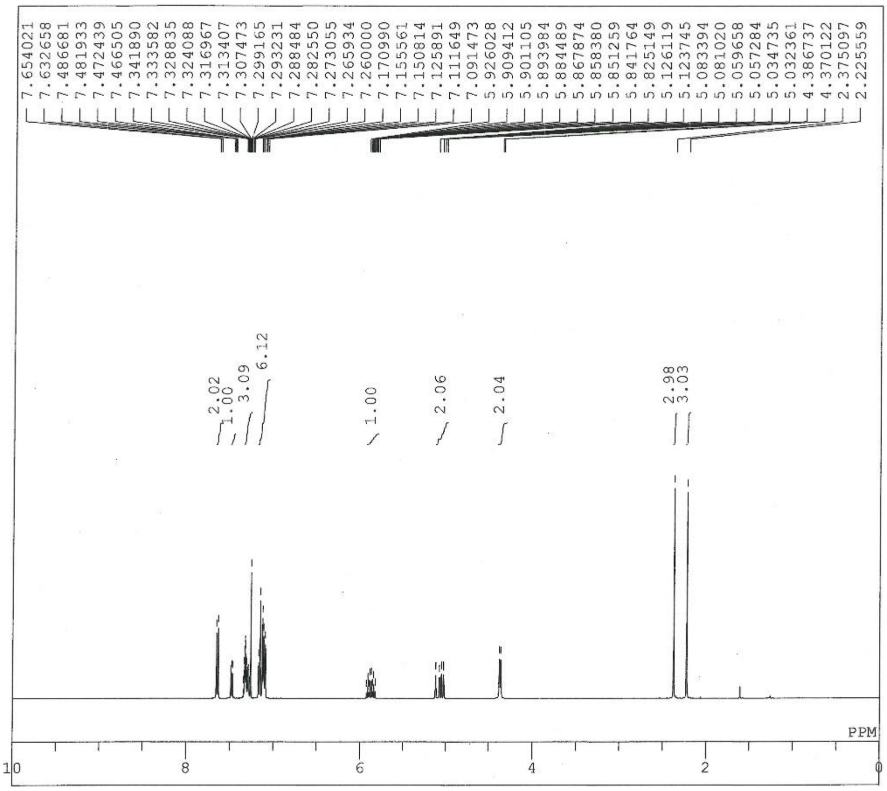

DFILE YO027 acetone removal 20
COMNT Qn-BOC 1H
DATIM 2021-12-15 14:44:50
OBNUC 1H
EXMOD single_pulse.ex2
OBFRQ 399.78 MHz
OBSET 4.62 KHz
OBFIN 5.98 Hz
POINT 16384
FREQU 7773.63 Hz
SCANS 32
ACQTM 2.1076 sec
PD 5.0000 sec
PW1 5.80 usec
IRNUC 1H
CTEMP 17.6 c
SLVNT CDCL3
EXREF 7.26 ppm
BF 0.12 Hz
RGAIN 30

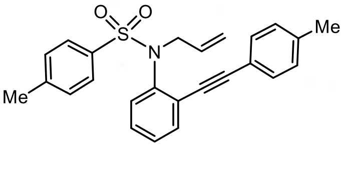

3

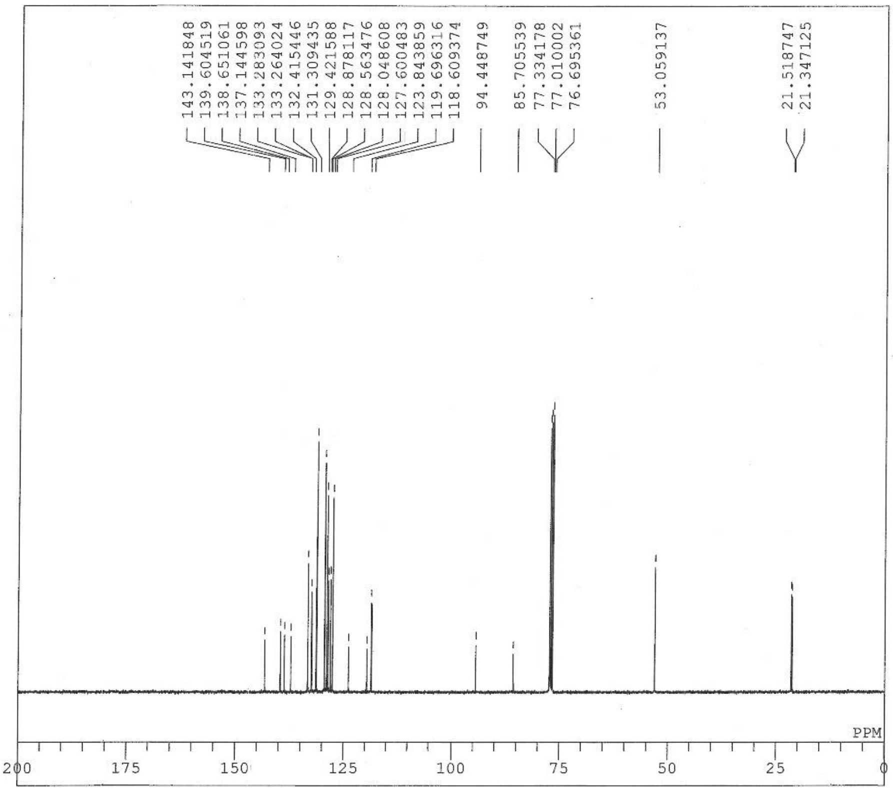

DFILE YO027 13C 20211215-1.als
COMNT
DATIM 2021-12-15 17:57:60
OBNUC 13C
EXMOD single_pulse_dec
OBFRQ 100.53 MHz
OBSET 5.35 KHz
OBFIN 5.86 Hz
POINT 32768
FREQU 31407.03 Hz
SCANS 1024
ACQTM 1.0433 sec
PD 2.0000 sec
PW1 3.23 usec
IRNUC 1H
CTEMP 18.9 c
SLVNT CDCL3
EXREF 77.01 ppm
BF 1.20 Hz
RGAIN 60

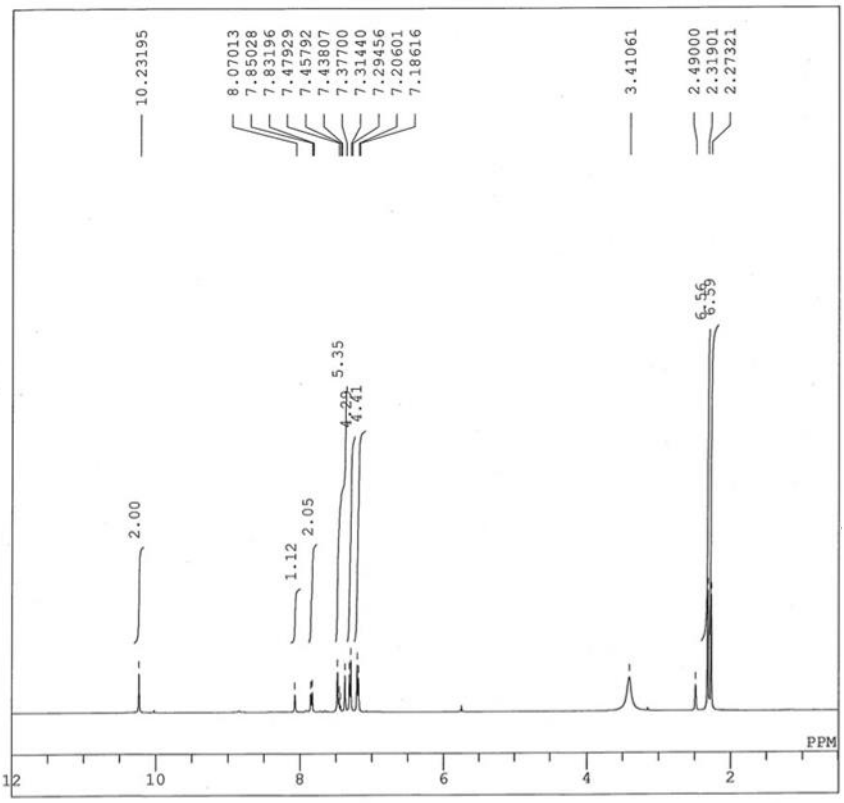

10.23195
8.07013
7.85028
7.83196
7.47929
7.45792
7.43807
7.37700
7.31440
7.29456
7.20601
7.18616
3.41061
2.49000
2.31901
2.27321
2.00
1.12
2.05
5.35
4.29
4.41
6.56
6.59
PPM
12
10
8
6
4
2
DFILE YO013 20201215 DMSO 2-1.al
COMNT Qn-BOC 1H
DATIM 2020-12-15 10:49:15
OBNUC 1H
EXMOD single_pulse.ex2
OBFRQ 399.78 MHz
OBSET 5.19 KHz
OBFIN 6.74 Hz
POINT 13107
FREQU 7999.88 Hz
SCANS 32
ACQTM 1.6384 sec
PD 5.0000 sec
PW1 5.17 usec
IRNUC 1H
CTEMP 17.1 c
SLVNT DMSO
EXREF 2.49 ppm
BF 0.12 Hz
RGAIN 36

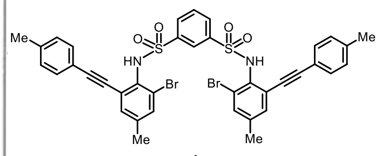

Me
HN
NH
Br
Br
Me
Me
Me

4

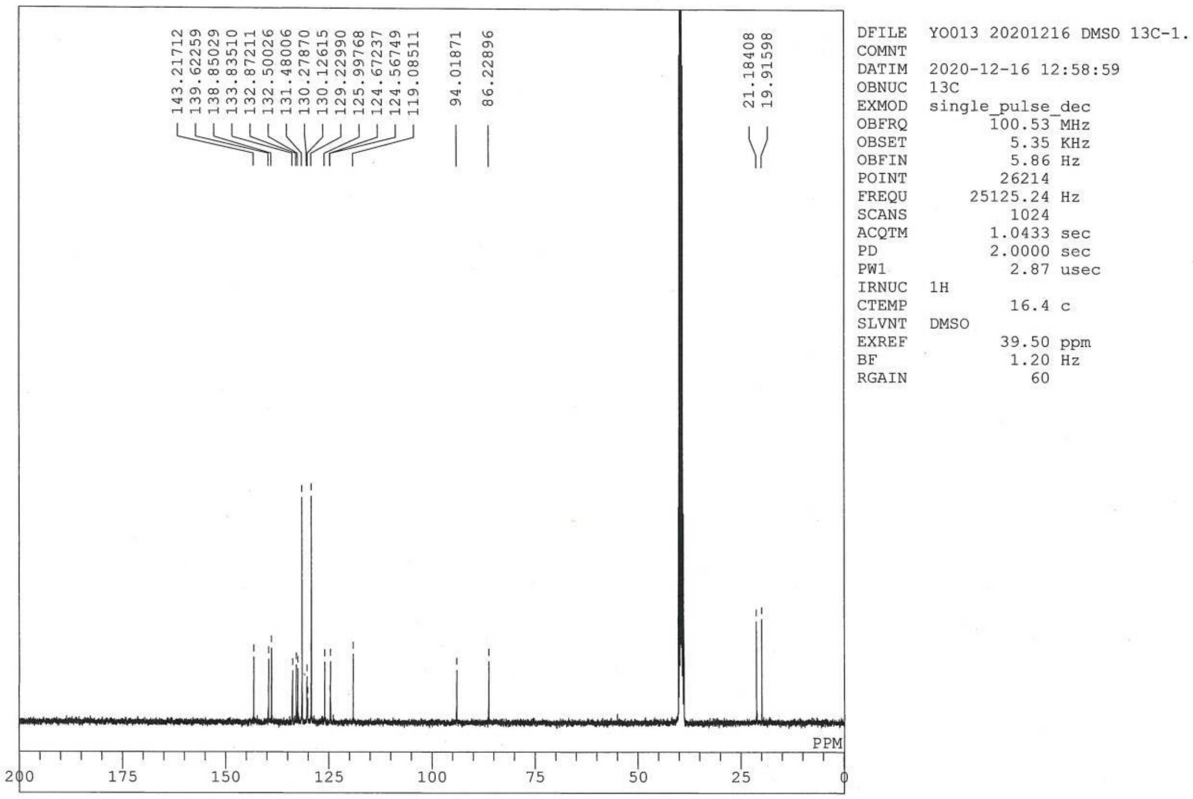

143.21712
139.62259
138.85029
133.83510
132.87211
132.50026
131.48006
130.27870
130.12615
129.22990
125.99768
124.67237
124.56749
119.08511
94.01871
86.22896
21.18408
19.91598
PPM
200
175
150
125
100
75
50
25
0
DFILE YO013 20201216 DMSO 13C-1.
COMNT
DATIM 2020-12-16 12:58:59
OBNUC 13C
EXMOD single_pulse_dec
OBFRQ 100.53 MHz
OBSET 5.35 KHz
OBFIN 5.86 Hz
POINT 26214
FREQU 25125.24 Hz
SCANS 1024
ACQTM 1.0433 sec
PD 2.0000 sec
PW1 2.87 usec
IRNUC 1H
CTEMP 16.4 c
SLVNT DMSO
EXREF 39.50 ppm
BF 1.20 Hz
RGAIN 60

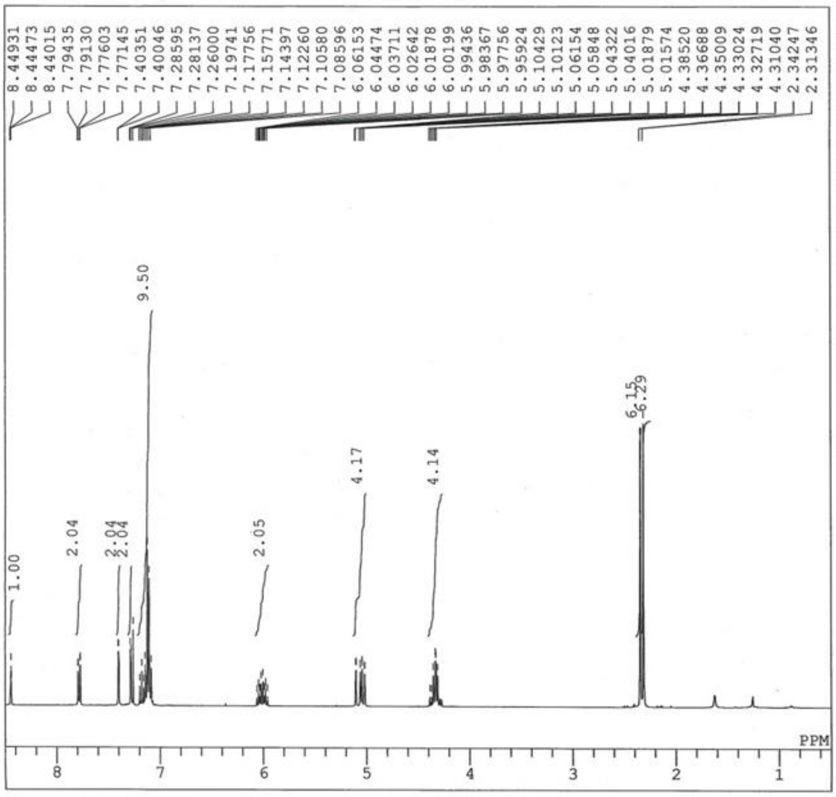

DFILE CN125 jyunnchiral 20201124
COMNT Qn-BOC 1H
DATIM 2020-11-24 13:26:23
OBNUC 1H
EXMOD single_pulse.ex2
OBFRQ 399.78 MHz
OBSET 5.19 KHz
OBFIN 6.74 Hz
POINT 13107
FREQU 7999.88 Hz
SCANS 32
ACQTM 1.6384 sec
PD 5.0000 sec
PW1 5.17 usec
IRNUC 1H
CTEMP 16.1 c
SLVNT CDCL3
EXREF 7.26 ppm
BF 0.12 Hz
RGAIN 30

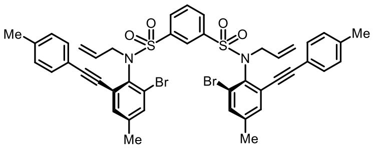

chiral-5

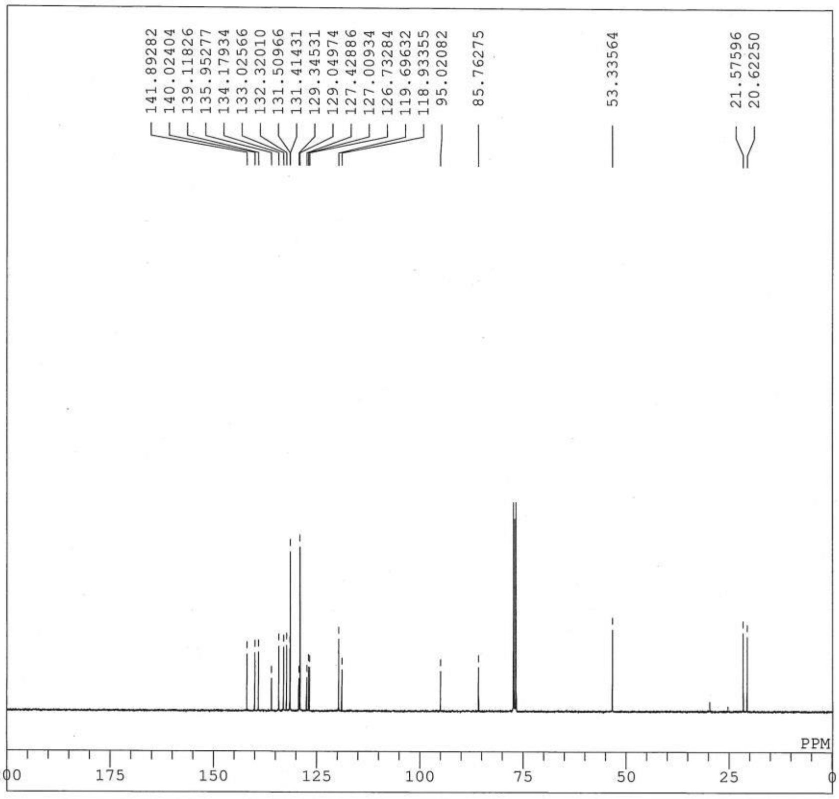

DFILE CN122 mplc 1st rac 13c 20
COMNT
DATIM 2020-09-29 13:42:03
OBNUC 13C
EXMOD single_pulse_dec
OBFRQ 100.53 MHz
OBSET 5.35 KHz
OBFIN 5.86 Hz
POINT 26214
FREQU 25125.24 Hz
SCANS 1024
ACQTM 1.0433 sec
PD 2.0000 sec
PW1 2.87 usec
IRNUC 1H
CTEMP 15.9 c
SLVNT CDCL3
EXREF 77.01 ppm
BF 1.20 Hz
RGAIN 60

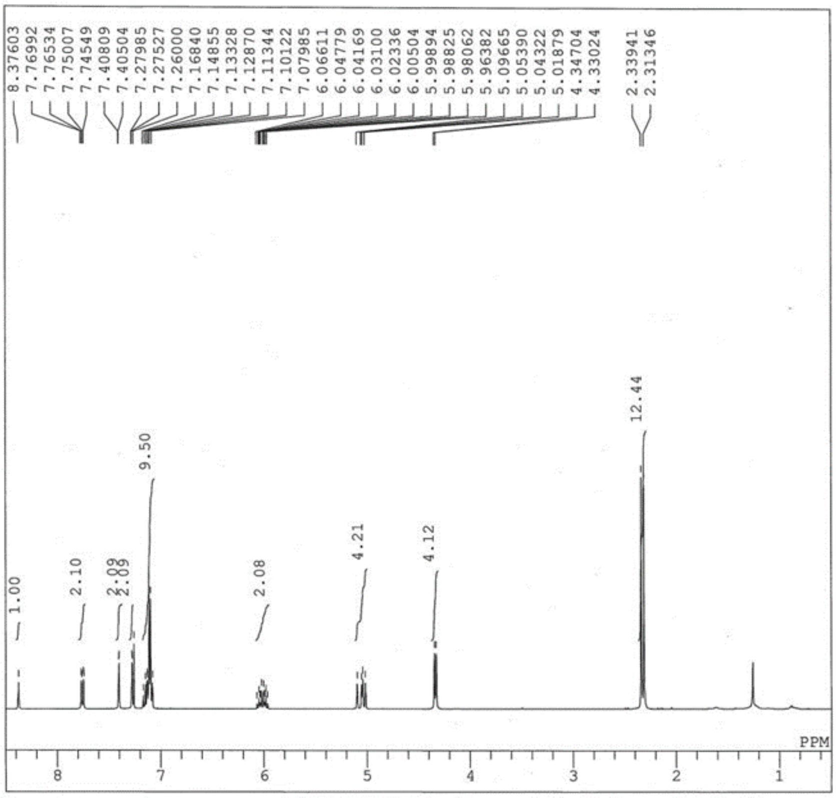

DFILE CN122 2nd 20201222-1.jdf
COMNT Qn-BOC 1H
DATIM 2020-12-22 13:48:33
OBNUC 1H
EXMOD single_pulse.ex2
OBFRQ 399.78 MHz
OBSET 5.19 KHz
OBFIN 6.74 Hz
POINT 16384
FREQU 10000.00 Hz
SCANS 32
ACQTM 1.6384 sec
PD 5.0000 sec
PW1 5.17 usec
IRNUC 1H
CTEMP 15.8 c
SLVNT CDCL3
EXREF 7.26 ppm
BF 0.12 Hz
RGAIN 30

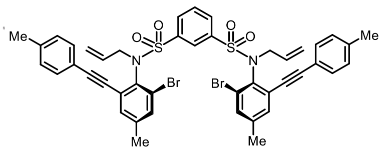

meso-5

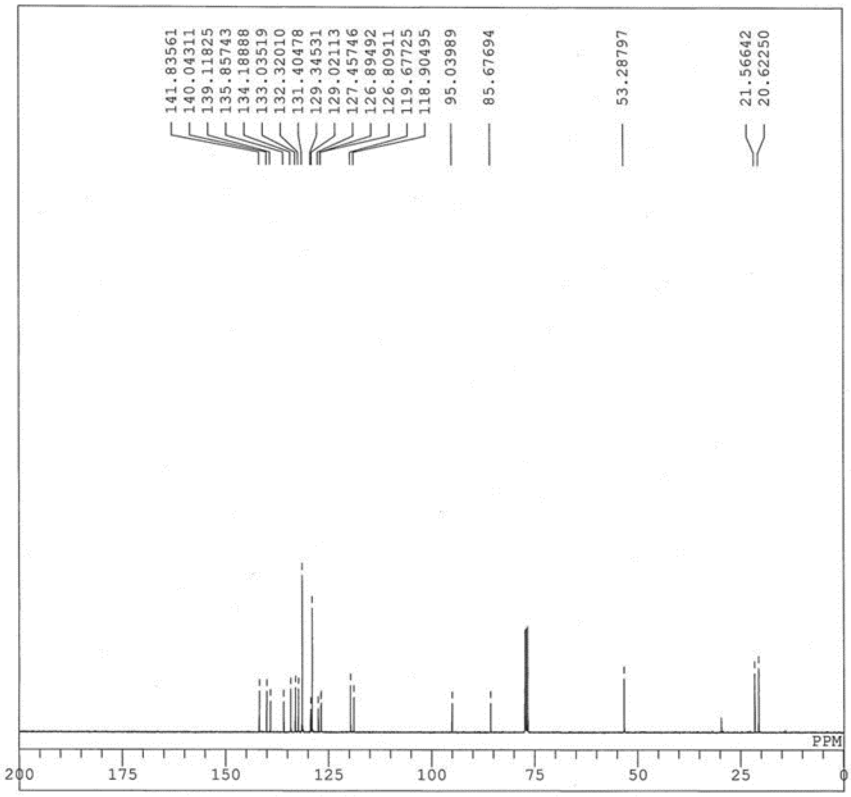

DFILE CN122 2nd 13c 20201222-1.a
COMNT
DATIM 2020-12-22 14:48:04
OBNUC 13C
EXMOD single_pulse_dec
OBFRQ 100.53 MHz
OBSET 5.35 KHz
OBFIN 5.86 Hz
POINT 26214
FREQU 25125.24 Hz
SCANS 1024
ACQTM 1.0433 sec
PD 2.0000 sec
PW1 2.87 usec
IRNUC 1H
CTEMP 16.8 c
SLVNT CDCL3
EXREF 77.01 ppm
BF 1.20 Hz
RGAIN 60

HPLC analysis using a chiral column for the determination of the ee

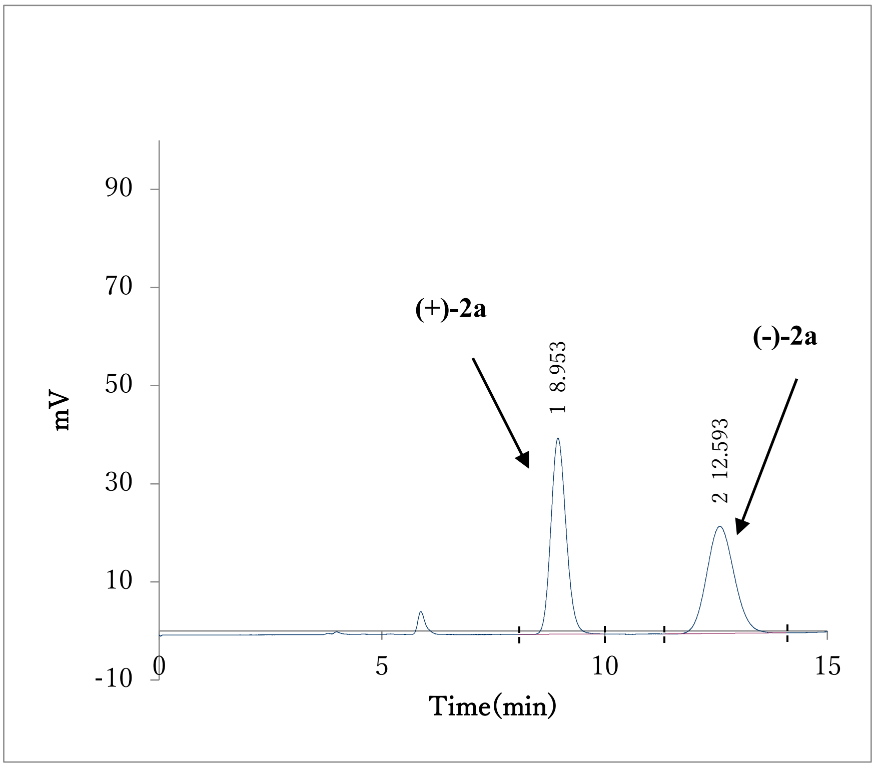

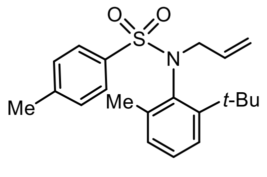

rac-**2a**

AS-H
eluent : 15% IPA in hexane
flow rate : 0.800 ml/min

No.	Rt	Area	Area(%)	Height
1	8.953	946346.6	50.3	39991
2	12.593	934059.8	49.7	21825
		1880406	100	61816

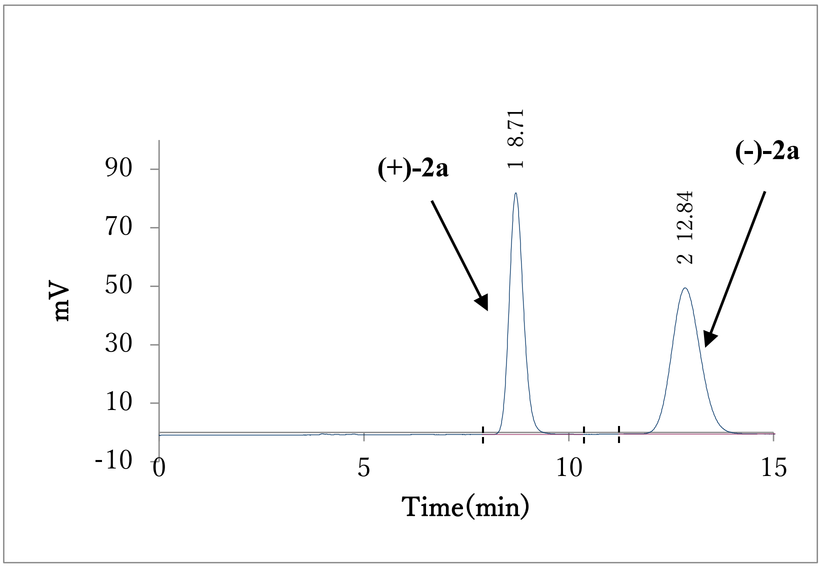

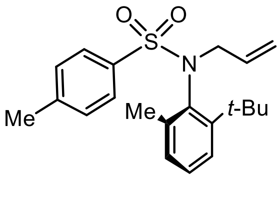

2a

AS-H
eluent : 15% IPA in hexane
flow rate : 0.800 ml/min

No.	Rt	Area	Area(%)	Height
1	8.71	1998473	45	82653
2	12.84	2447382	55	50024
		4445855	100	132677

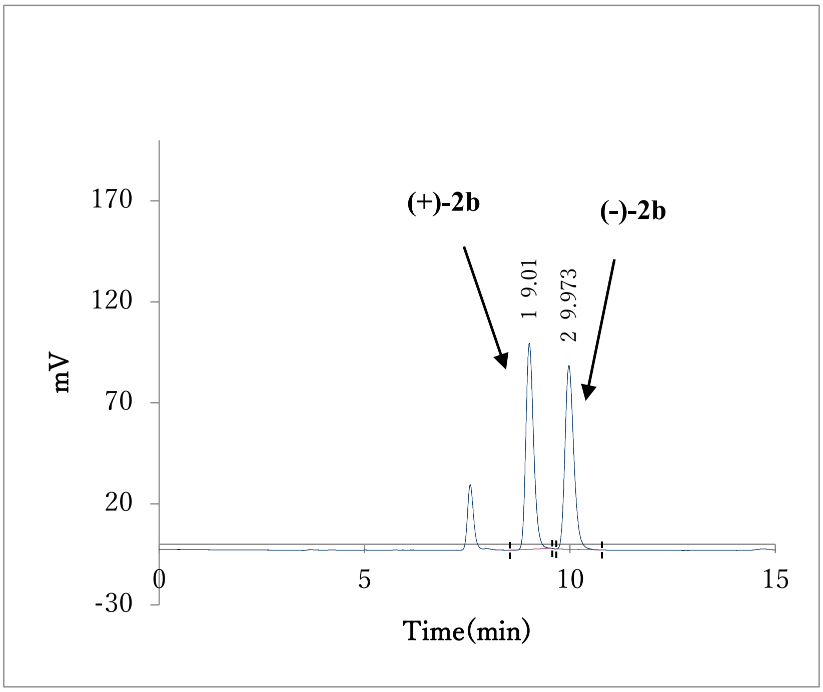

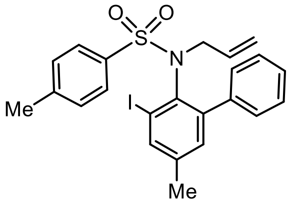

rac-**2b**

OD-3
eluent : 15% IPA in hexane
flow rate : 0.800 ml/min

No.	Rt	Area	Area(%)	Height
1	9.01	1335462	49.8	102013
2	9.973	1348567	50.2	90983
		2684030	100	192996

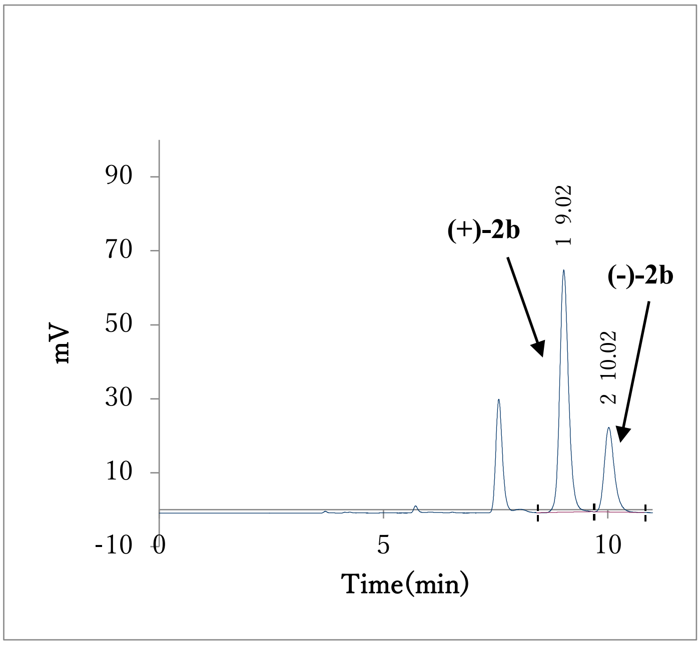

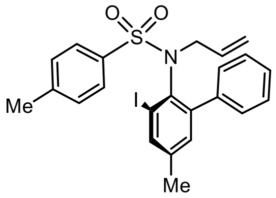

2b

OD-3
eluent : 15% IPA in hexane
flow rate : 0.800 ml/min

No.	Rt	Area	Area(%)	Height
1	9.02	897741.6	71.7	65538
2	10.02	353680.6	28.3	22887
		1251422	100	88425

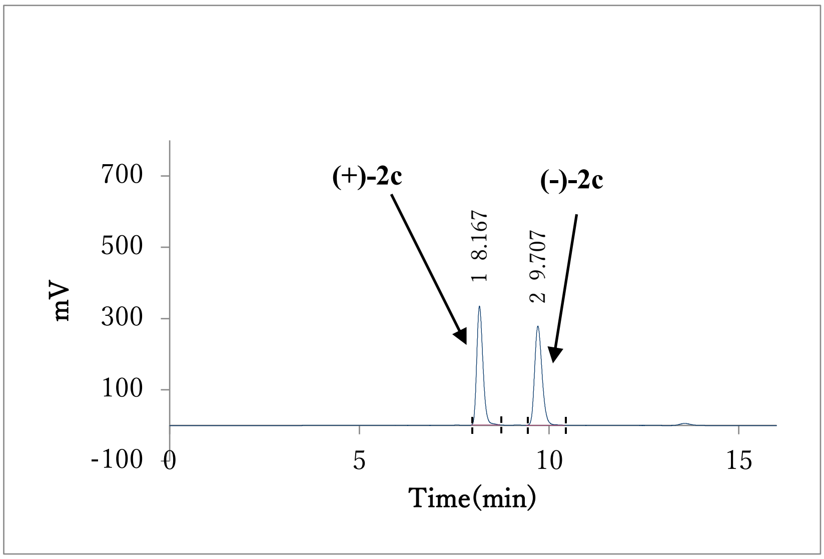

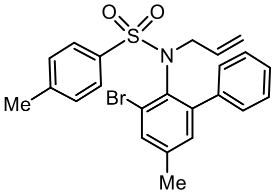

rac-**2c**

OD-3
eluent : 15% IPA in hexane
flow rate : 0.800 ml/min

No.	Rt	Area	Area(%)	Height
1	8.167	3774489	49.9	334226
2	9.707	3784213	50.1	278629
			7558702	100

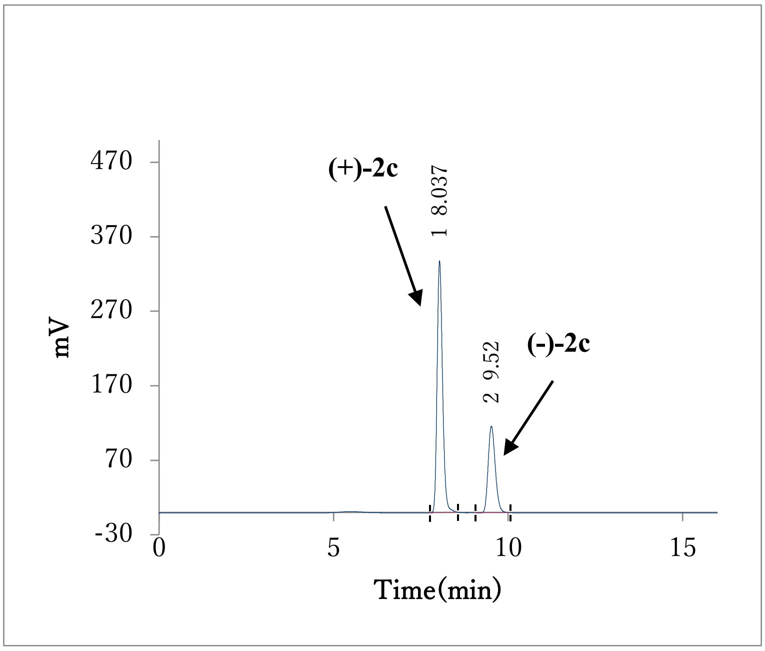

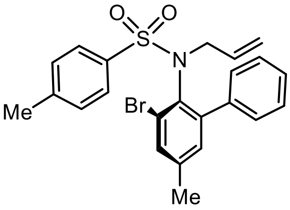

2c

OD-3
eluent : 15% IPA in hexane
flow rate : 0.800 ml/min

No.	Rt	Area	Area(%)	Height
1	8.037	3637341	69	337996
2	9.52	1631538	31	116315
		5268879	100	454311

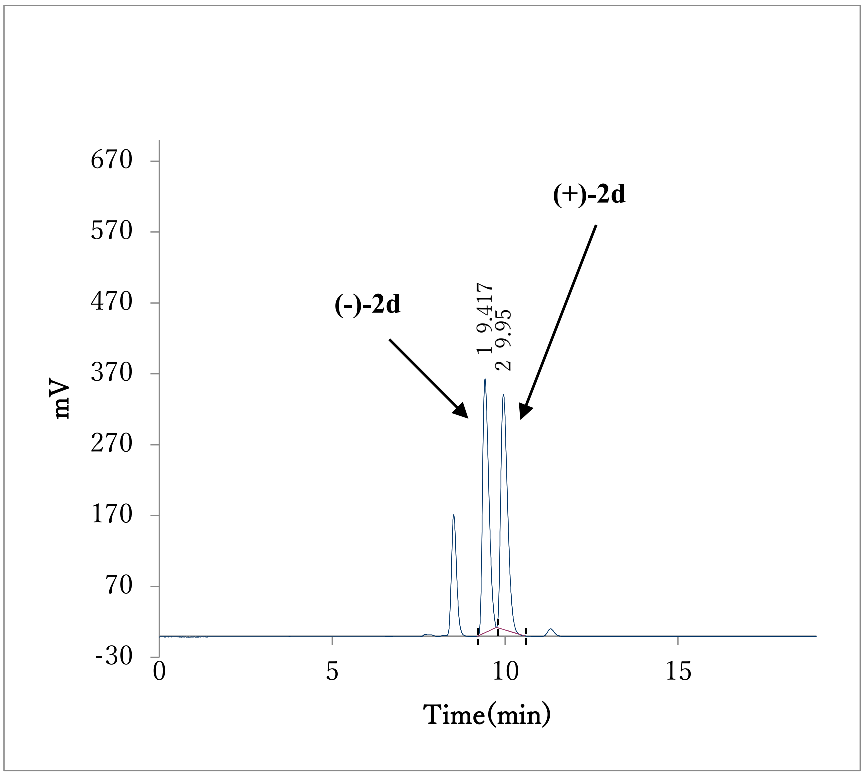

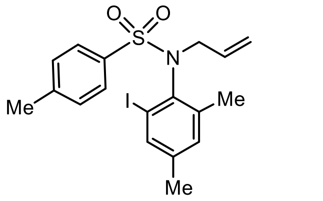

rac-**2d**

OD-3
eluent : 3% IPA in hexane
flow rate : 0.800 ml/min

No.	Rt	Area	Area(%)	Height
1	9.417	4555926	50.1	358224
2	9.95	4543942	49.9	332272
		9099868	100	690496

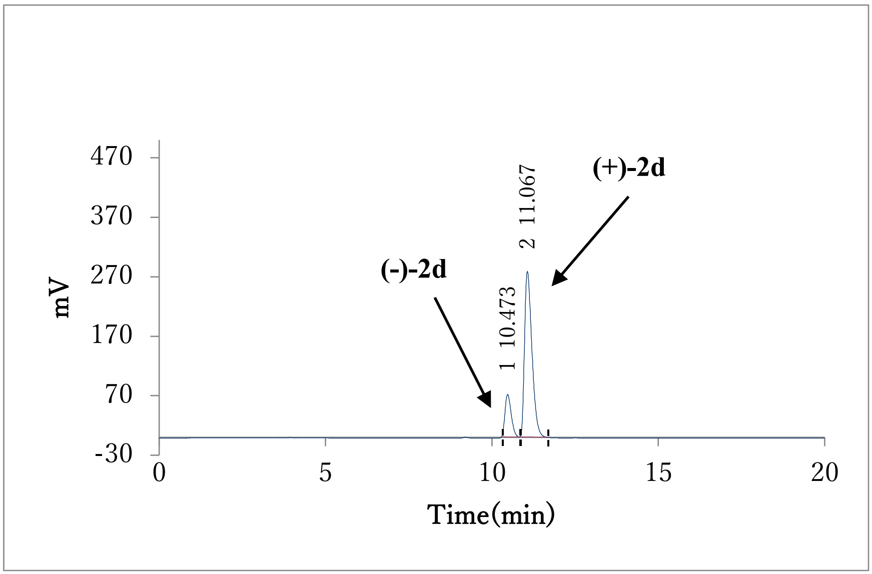

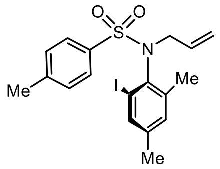

2d

OD-3
eluent : 3% IPA in hexane
flow rate : 0.800 ml/min

No.	Rt	Area	Area(%)	Height
1	10.473	917391.8	17.6	71148
2	11.067	4289202	82.4	278279
		5206593	100	349427

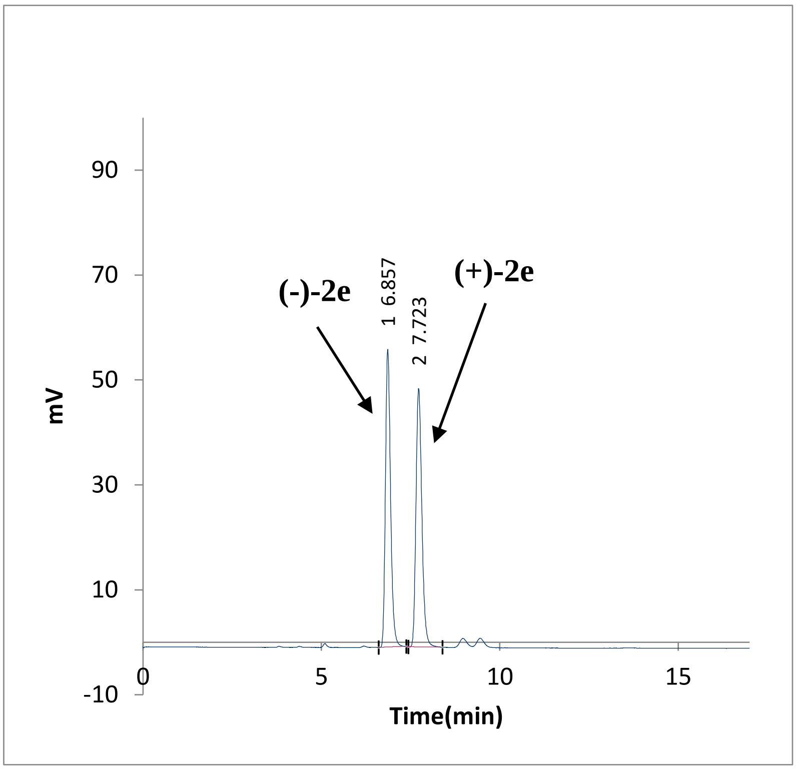

No.	Rt	Area	Area(%)	Height
1	6.857	566012	50.2	56774
2	7.723	562544.7	49.8	49290
		1128557	100	106064

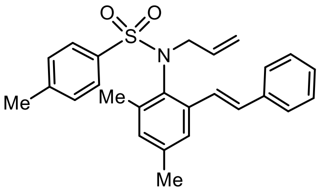

rac-**2e**

OD-3
eluent : 15% IPA in hexane
flow rate : 0.800 ml/min

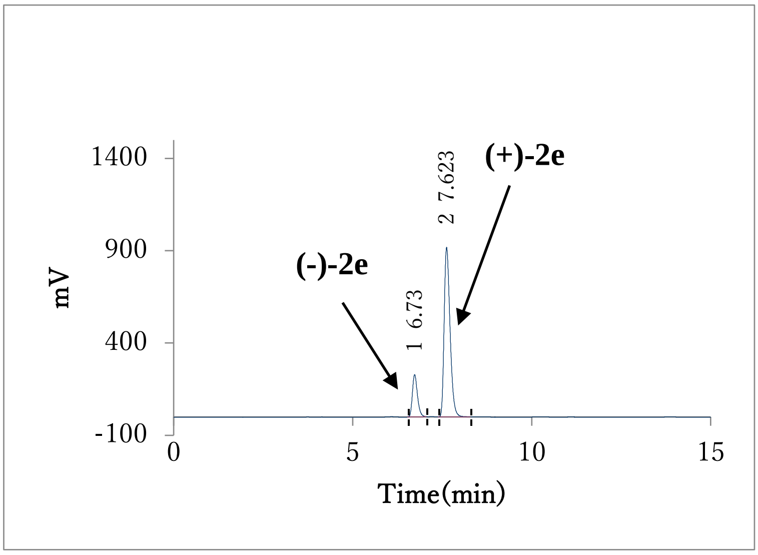

No.	Rt	Area	Area(%)	Height
1	6.73	2245655	17.6	229431
2	7.623	10527822	82.4	919828
		12773477	100	1149259

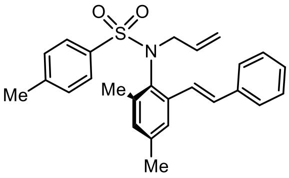

2e

OD-3
eluent : 15% IPA in hexane
flow rate : 0.800 ml/min

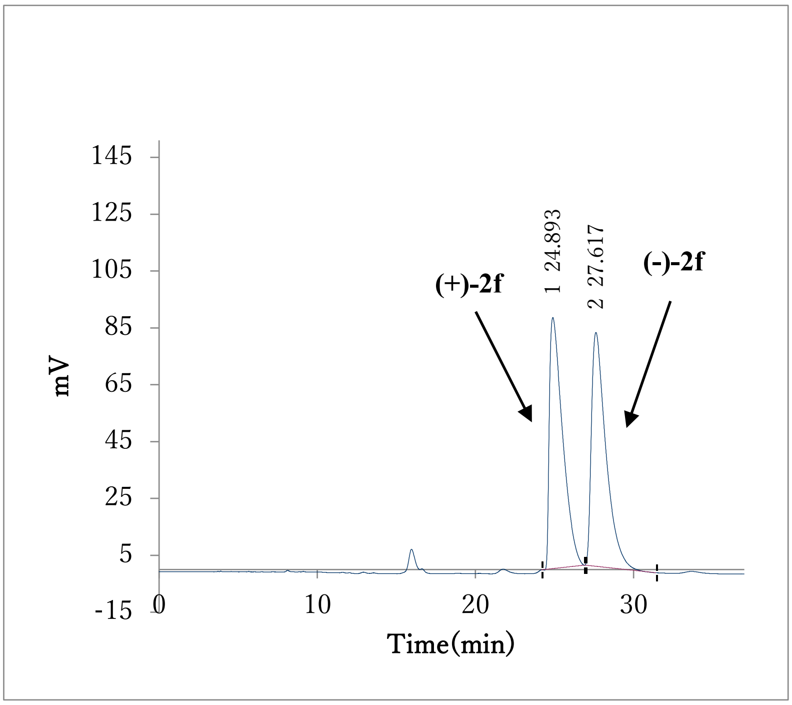

No.	Rt	Area	Area(%)	Height
1	24.893	5126023	49.4	88320
2	27.617	5252101	50.6	82317
		10378124	100	170637

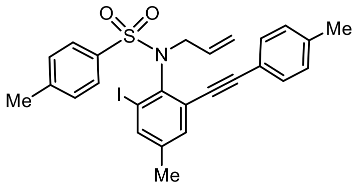

rac-**2f**

OD-3
eluent : 3% IPA in hexane
flow rate : 0.800 ml/min

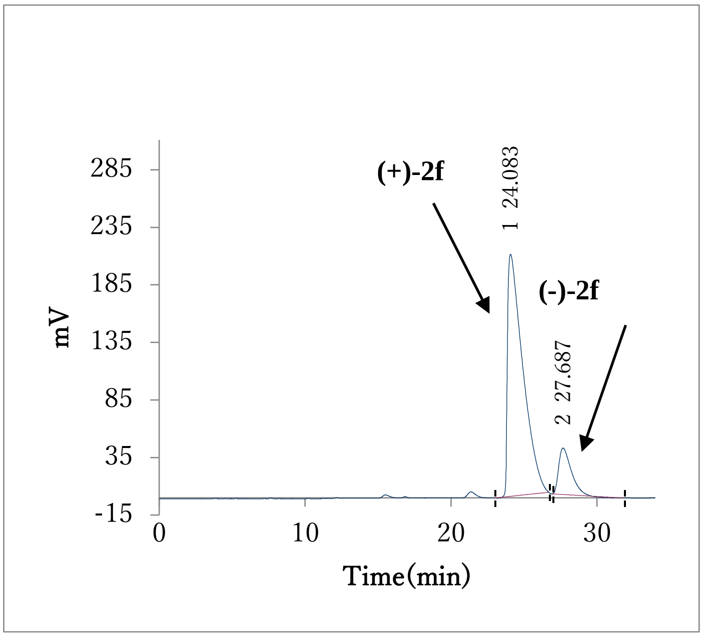

No.	Rt	Area	Area(%)	Height
1	24.083	15383704	86.5	210327
2	27.687	2392105	13.5	40430
		17775809	100	250757

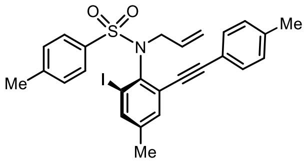

2f

OD-3
eluent : 3% IPA in hexane
flow rate : 0.800 ml/min

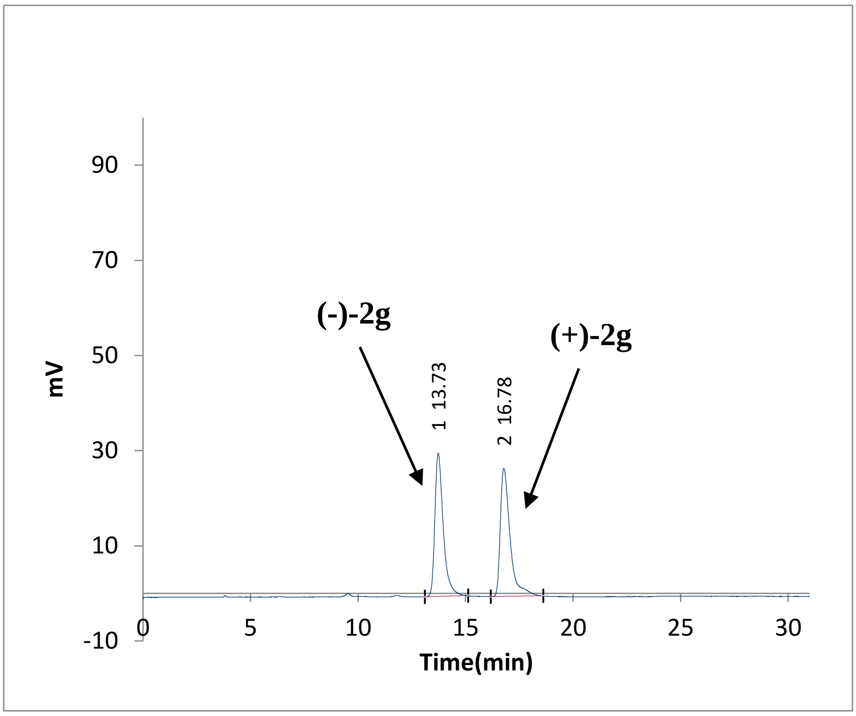

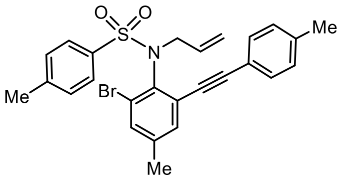

rac-**2g**
OD-3
eluent : 5% IPA in hexane
flow rate : 0.800 ml/min

No.	Rt	Area	Area(%)	Height
1	13.73	818781.6	49.2	30108
2	16.78	846494.6	50.8	26893
		1665276	100	57001

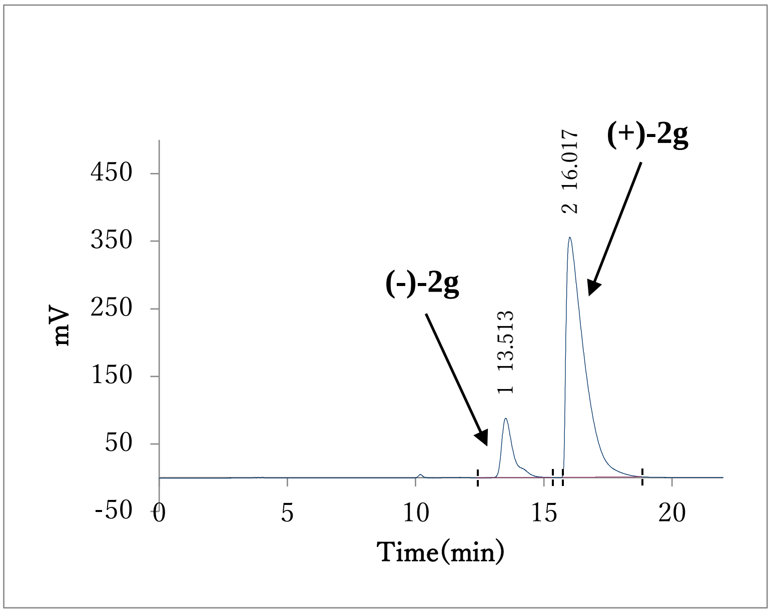

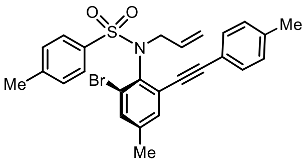

2g
OD-3
eluent : 5% IPA in hexane
flow rate : 0.800 ml/min

No.	Rt	Area	Area(%)	Height
1	13.513	2792384	13.8	88046
2	16.017	17404710	86.2	355501
		20197094	100	443547

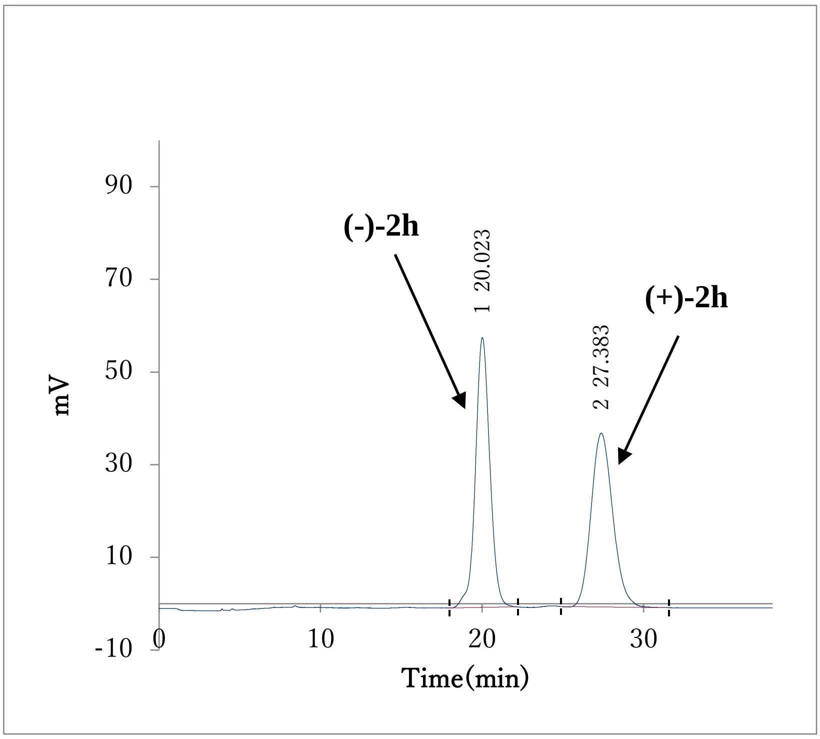

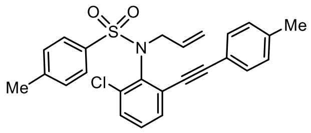

rac-**2h**

AS-H
eluent : 15% IPA in hexane
flow rate : 0.800 ml/min

No.	Rt	Area	Area(%)	Height
1	20.023	3607239	50.8	58265
2	27.383	3498382	49.2	37534
		7105622	100	95799

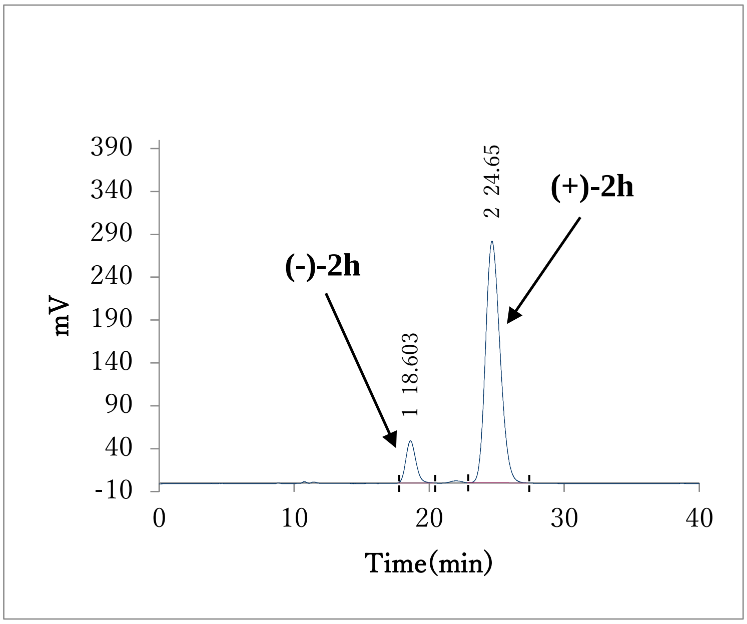

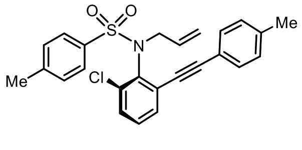

2h

AS-H
eluent : 15% IPA in hexane
flow rate : 0.800 ml/min

No.	Rt	Area	Area(%)	Height
1	18.603	2516148	10.7	49327
2	24.65	21061843	89.3	281780
		23577991	100	331107

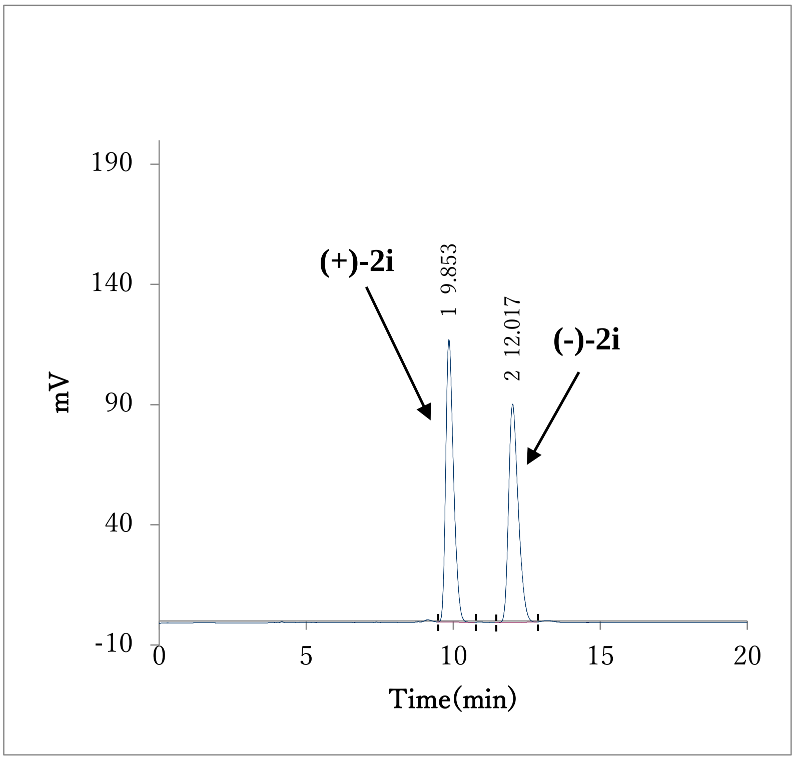

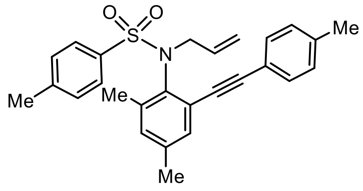

rac-**2i**

AD-H
eluent : 15% IPA in hexane
flow rate : 0.800 ml/min

No.	Rt	Area	Area(%)	Height
1	9.853	2160478	51.1	117545
2	12.017	2070706	48.9	90830
		4231184	100	208375

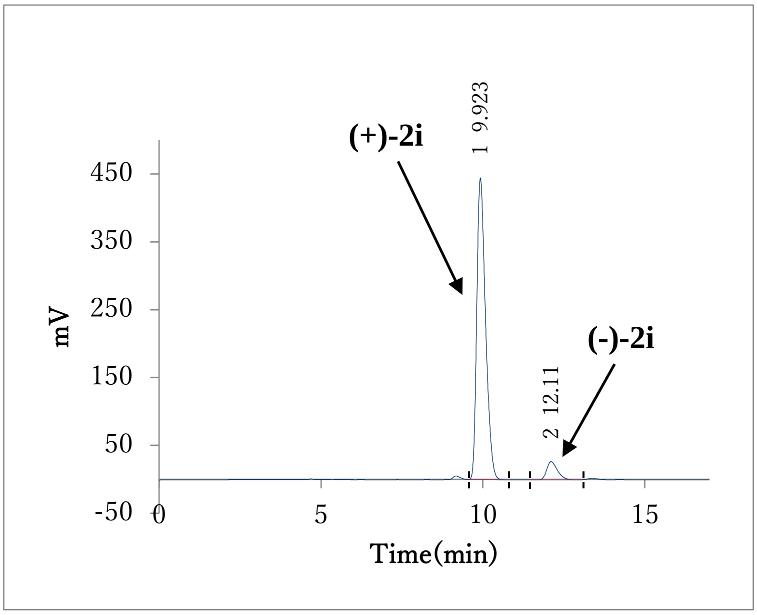

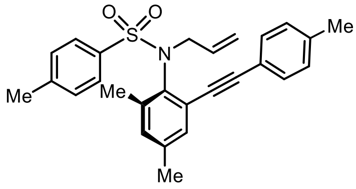

2i

AD-H
eluent : 15% IPA in hexane
flow rate : 0.800 ml/min

No.	Rt	Area	Area(%)	Height
1	9.923	8352218	93.1	444591
2	12.11	616146.6	6.9	26956
		8968364	100	471547

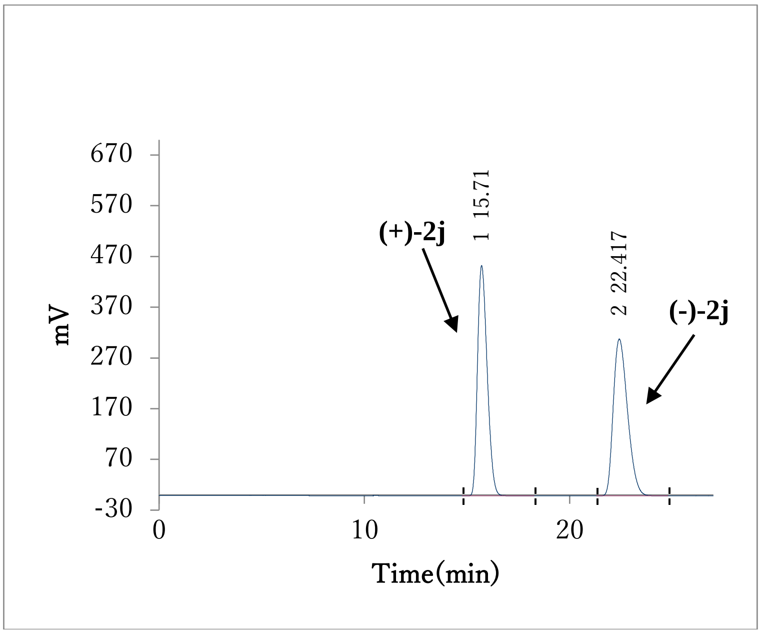

No.	Rt	Area	Area(%)	Height
1	15.71	14871225	50	454163
2	22.417	14882932	50	309231
		29754157	100	763394

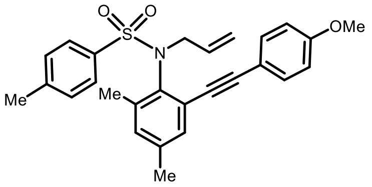

rac-**2j**

AD-H
eluent : 15% IPA in hexane
flow rate : 0.800 ml/min

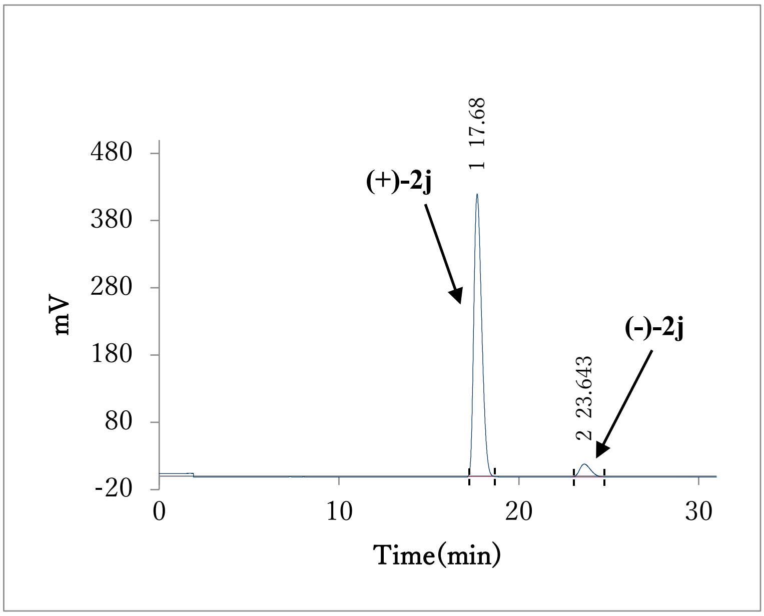

No.	Rt	Area	Area(%)	Height
1	17.68	12792973	94	420224
2	23.643	812021.8	6	19140
		13604994	100	439364

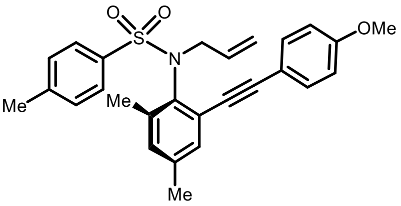

2j

AD-H
eluent : 15% IPA in hexane
flow rate : 0.800 ml/min

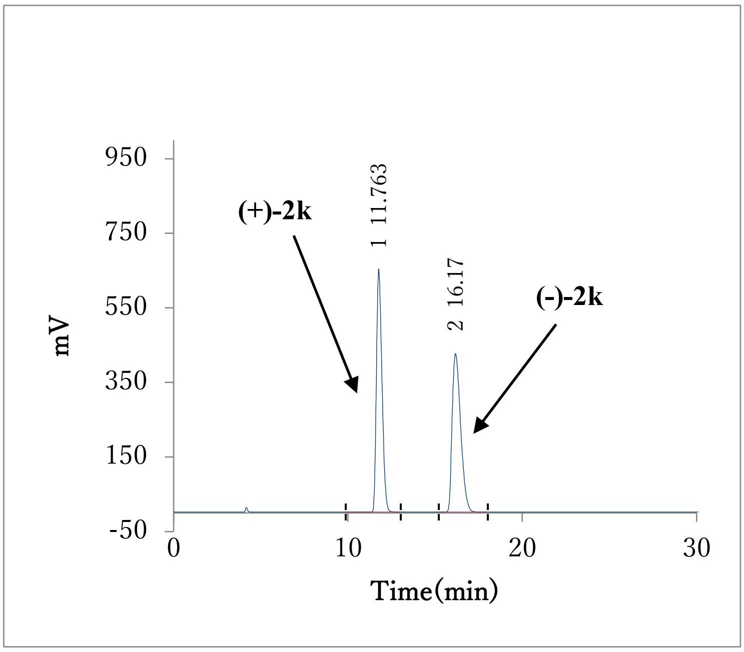

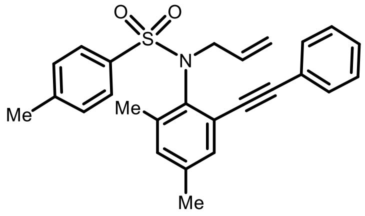

rac-**2k**

AD-H
eluent : 15% IPA in hexane
flow rate : 0.800 ml/min

No.	Rt	Area	Area(%)	Height
1	11.763	14414348	50	652379
2	16.17	14430555	50	425141
		28844903	100	1077520

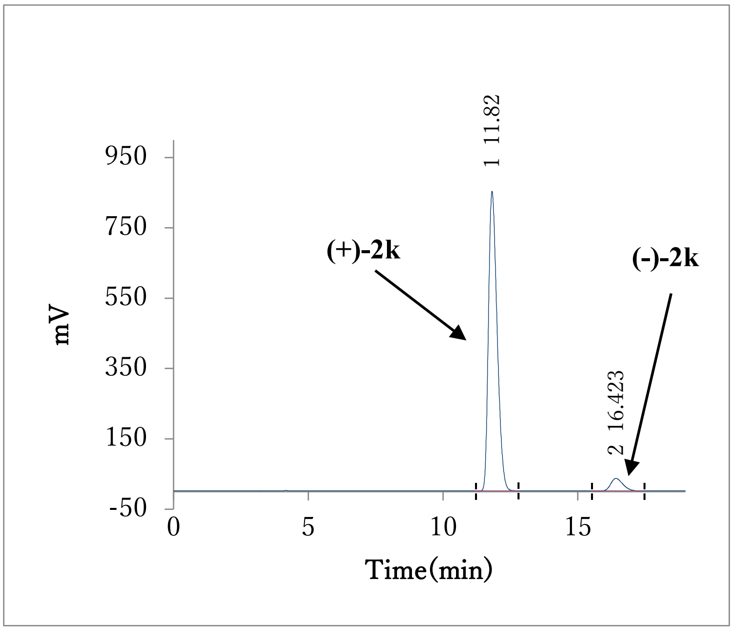

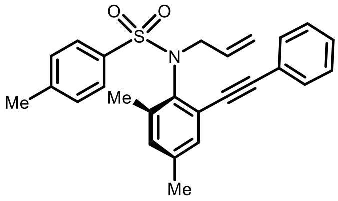

2k

AD-H
eluent : 15% IPA in hexane
flow rate : 0.800 ml/min

No.	Rt	Area	Area(%)	Height
1	11.82	19003569	94.5	851886
2	16.423	1097074	5.5	35370
		20100642	100	887256

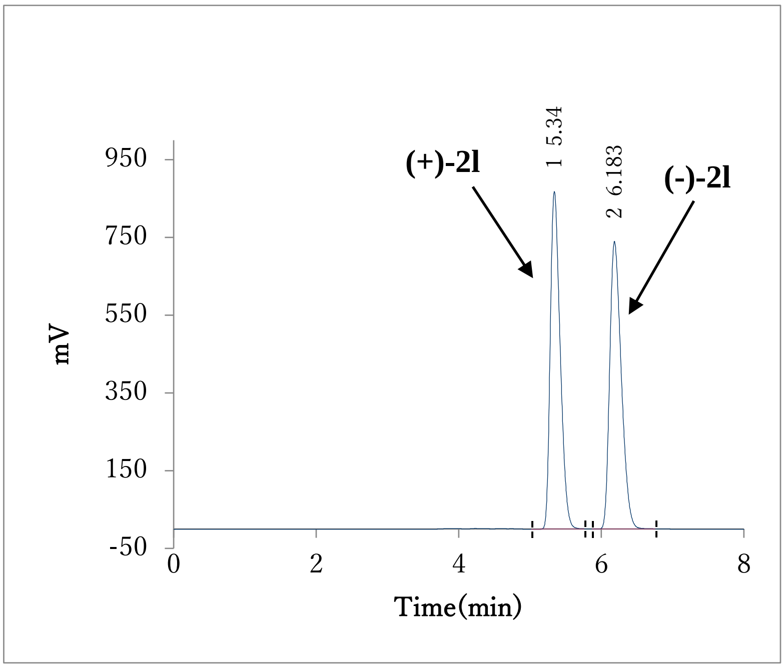

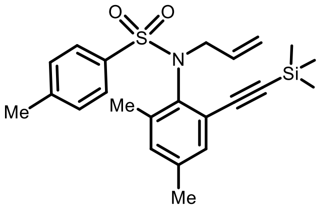

rac-**2l**

AD-H
eluent : 15% IPA in hexane
flow rate : 0.800 ml/min

No.	Rt	Area	Area(%)	Height
1	5.34	8351457	50	868501
2	6.183	8354624	50	739717
		16706081	100	1608218

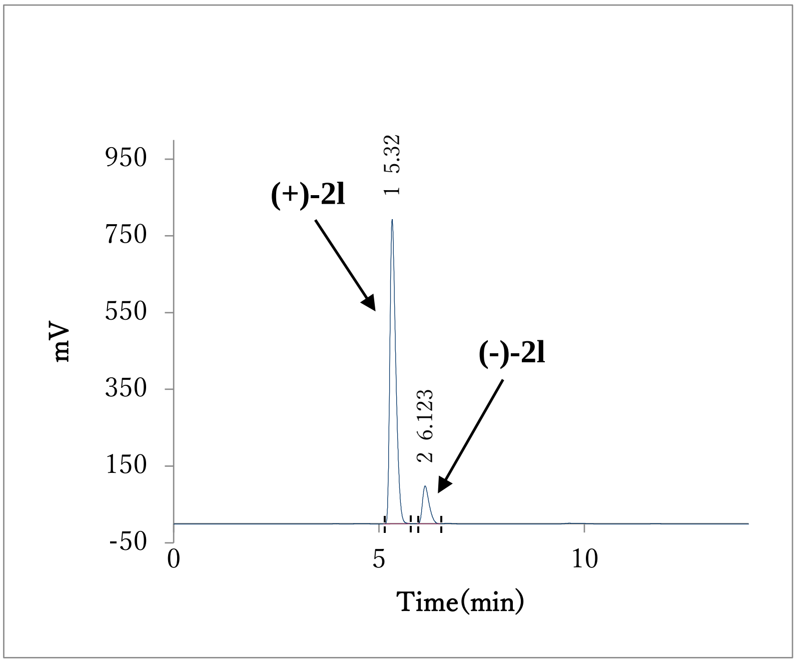

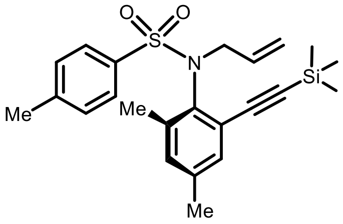

2l

AD-H
eluent : 15% IPA in hexane
flow rate : 0.800 ml/min

No.	Rt	Area	Area(%)	Height
1	5.32	7899865	87.6	793544
2	6.123	1116536	12.4	98740
		9016401	100	892284

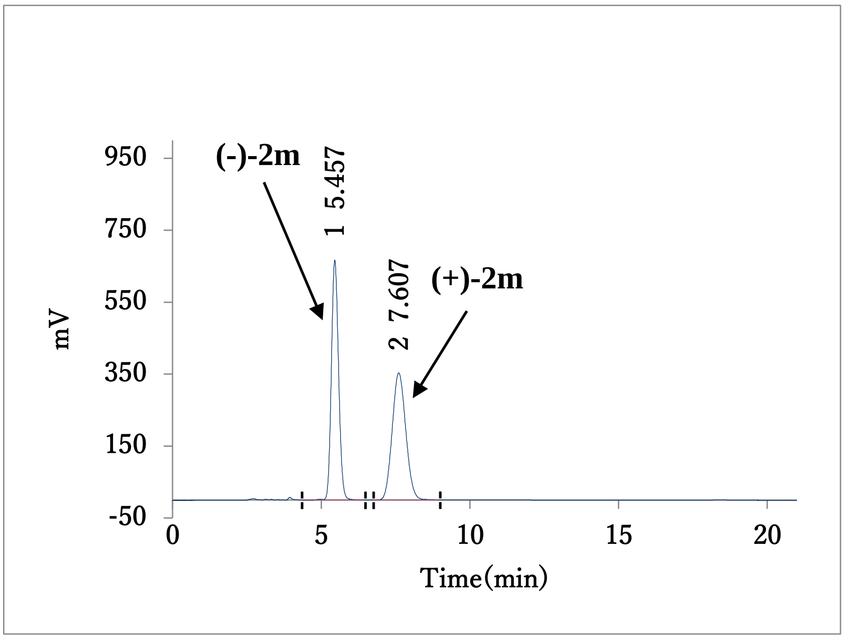

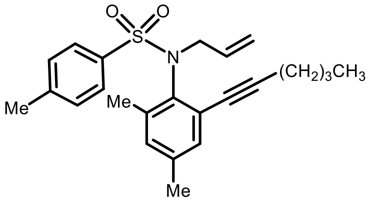

rac-**2m**

AS-H
eluent : 15% IPA in hexane
flow rate : 0.800 ml/min

No.	Rt	Area	Area(%)	Height
1	5.457	11207659	50	667207
2	7.607	11217198	50	353275
		22424857	100	1020482

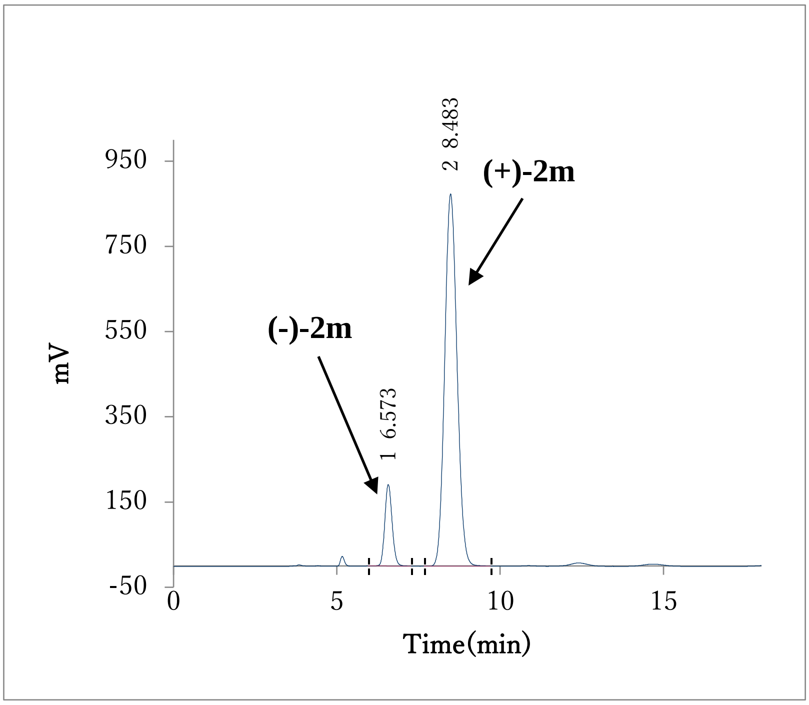

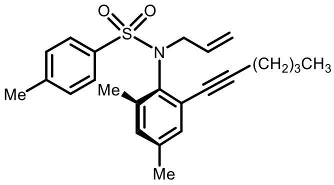

2m

AS-H
eluent : 15% IPA in hexane
flow rate : 0.800 ml/min

No.	Rt	Area	Area(%)	Height
1	6.573	2984160	11.5	191397
2	8.483	22926038	88.5	873678
		25910198	100	1065075

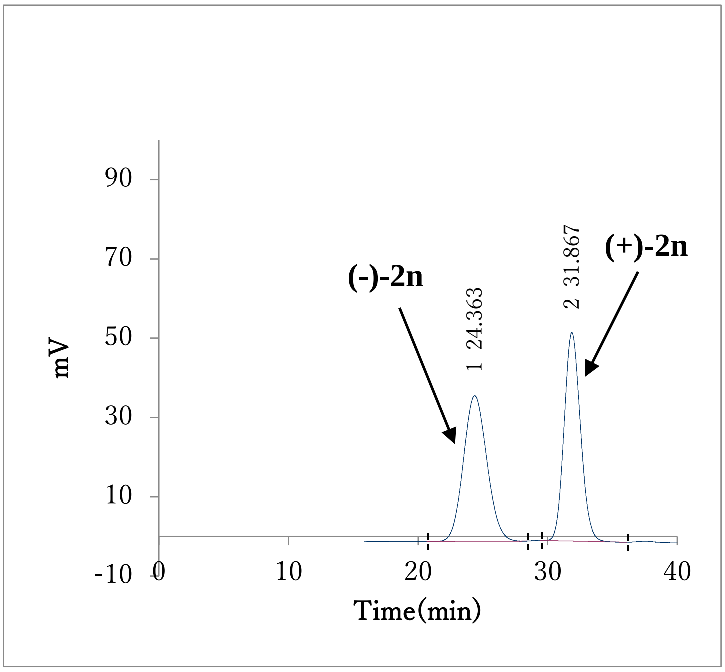

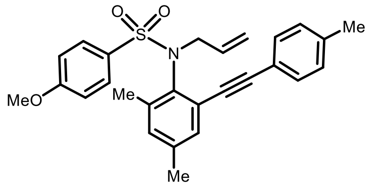

rac-**2n**

AS-H
eluent : 5% IPA in hexane
flow rate : 0.800 ml/min

No.	Rt	Area	Area(%)	Height
1	24.363	4648267	50.1	36764
2	31.867	4630188	49.9	52617
		9278454	100	89381

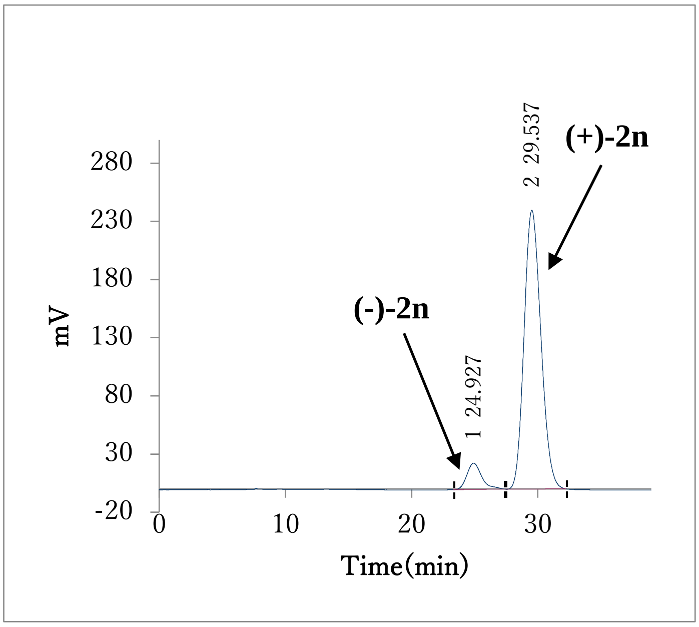

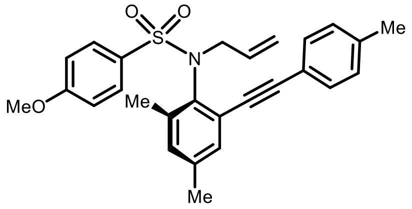

2n

AS-H
eluent : 5% IPA in hexane
flow rate : 0.800 ml/min

No.	Rt	Area	Area(%)	Height
1	24.927	1836889	7.6	22609
2	29.537	22208298	92.4	239673
		24045187	100	262282

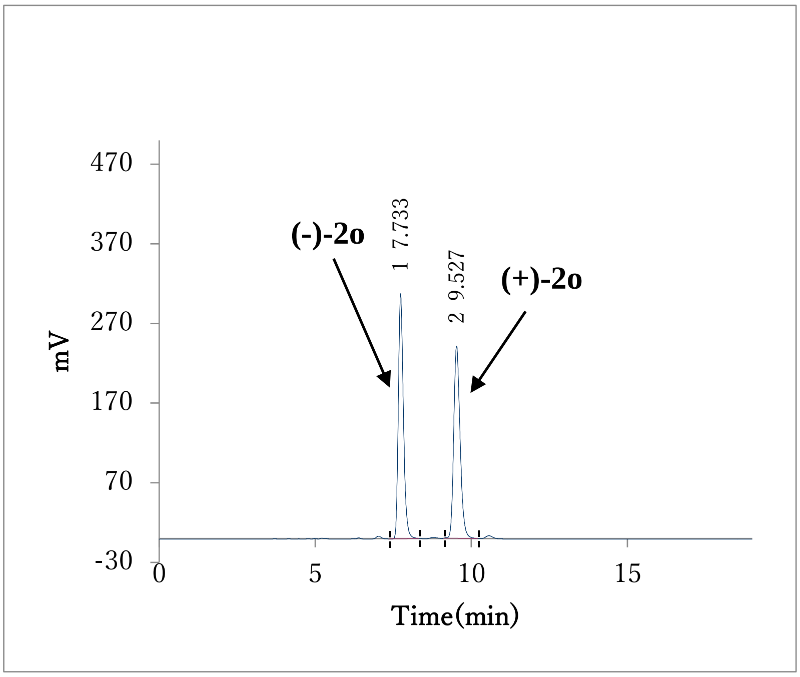

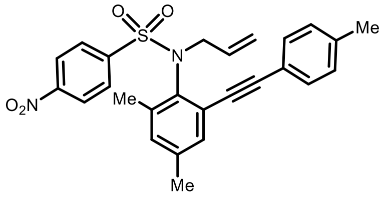

rac-**2o**

OD-3
eluent : 15% IPA in hexane
flow rate : 0.800 ml/min

No.	Rt	Area	Area(%)	Height
1	7.733	3467087	50.3	307779
2	9.527	3420459	49.7	241565
		6887546	100	549344

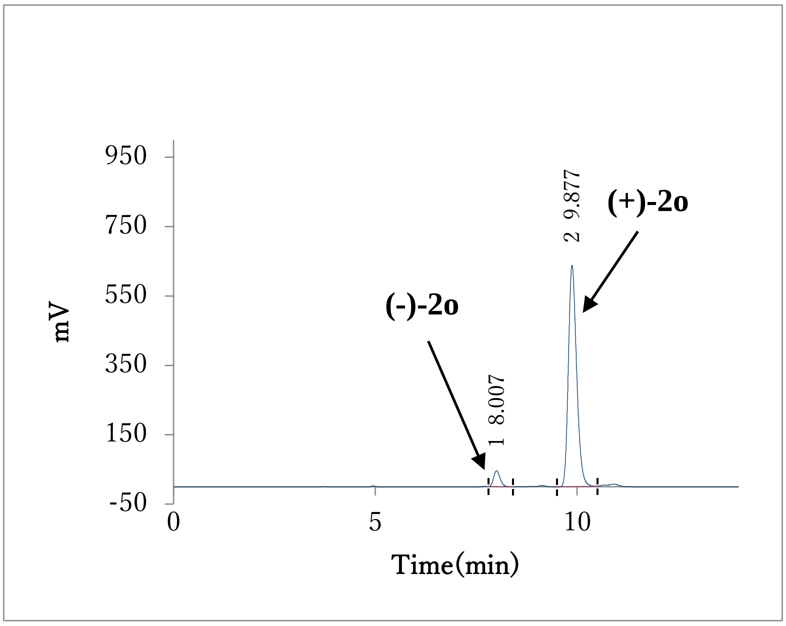

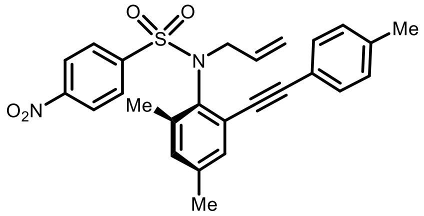

2o

OD-3
eluent : 15% IPA in hexane
flow rate : 0.800 ml/min

No.	Rt	Area	Area(%)	Height
1	8.007	511222.6	5.3	45935
2	9.877	9046738	94.7	638258
		9557961	100	684193

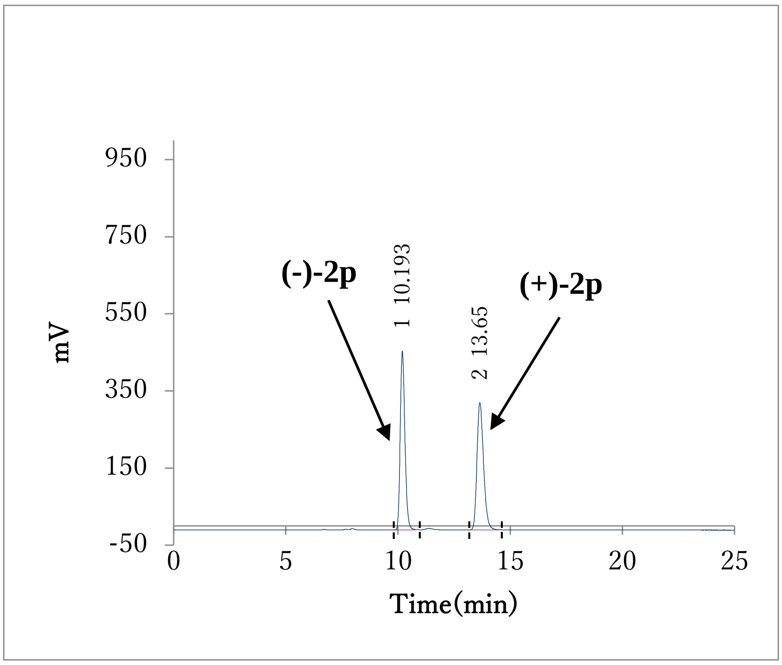

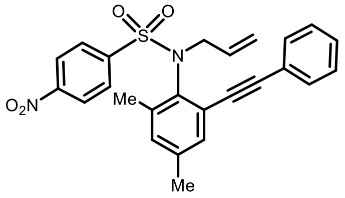

rac-**2p**

OD-3
eluent : 15% IPA in hexane
flow rate : 0.800 ml/min

No.	Rt	Area	Area(%)	Height
1	10.193	6732828	50	463875
2	13.65	6736503	50	329762
		13469331	100	793637

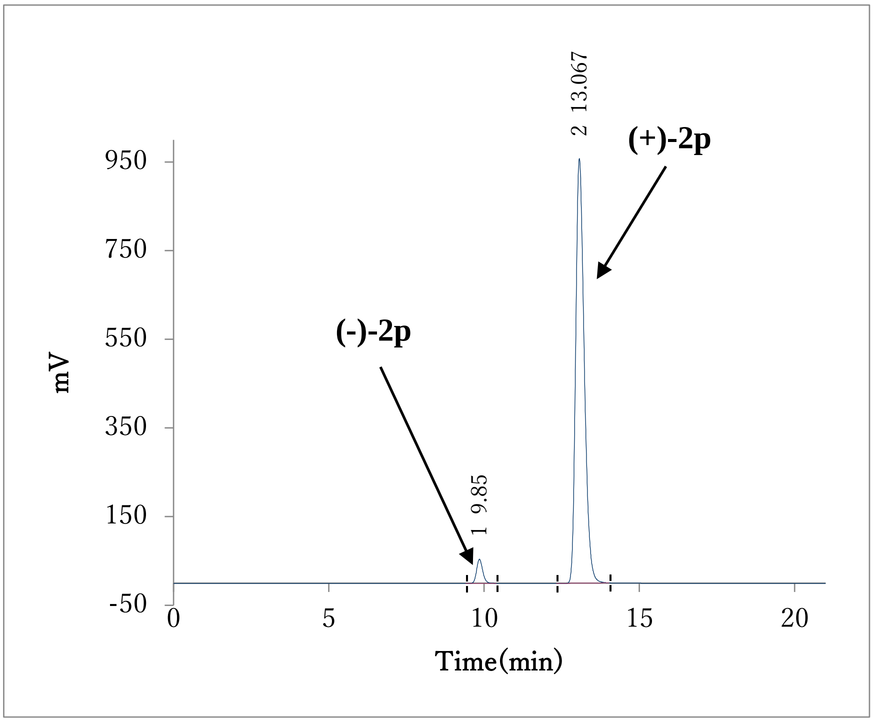

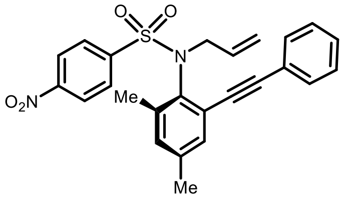

2p

OD-3
eluent : 15% IPA in hexane
flow rate : 0.800 ml/min

No.	Rt	Area	Area(%)	Height
1	9.85	742086.5	3.9	54500
2	13.067	18326906	96.1	958185
		19068993	100	1012685

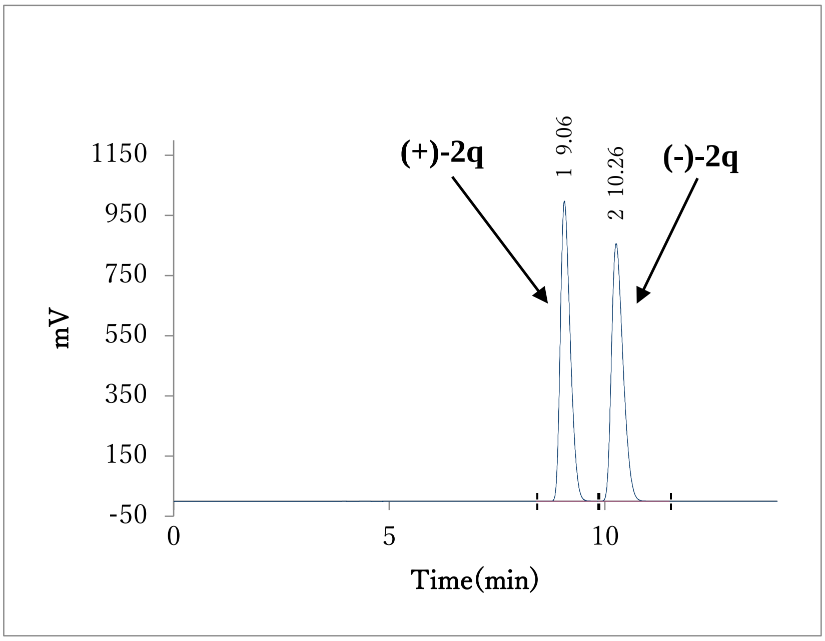

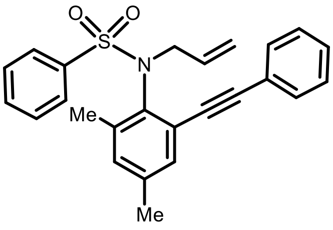

rac-**2q**

AD-H
eluent : 15% IPA in hexane
flow rate : 0.800 ml/min

No.	Rt	Area	Area(%)	Height
1	9.06	15948835	50	997995
2	10.26	15969247	50	856846
		31918082	100	1854841

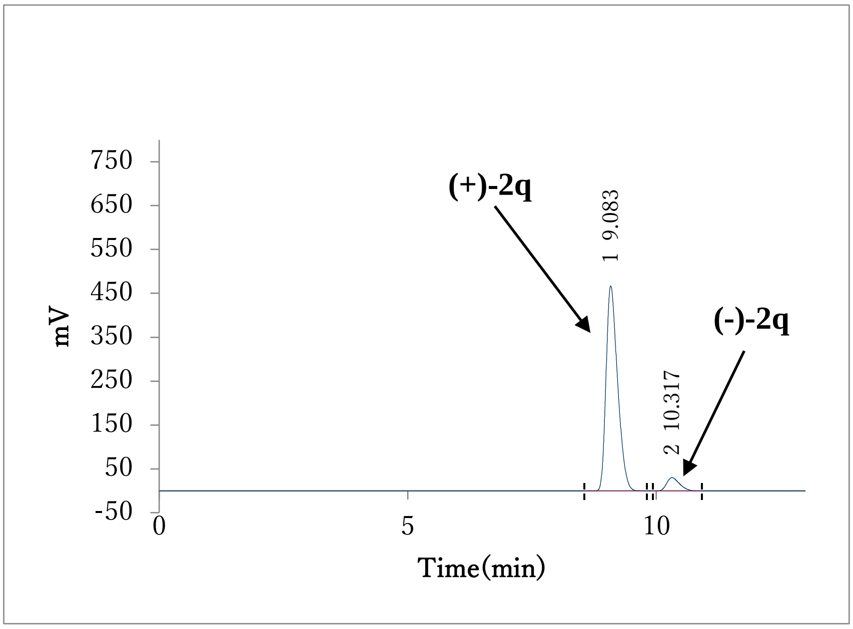

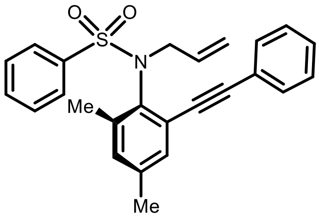

2q

AD-H
eluent : 15% IPA in hexane
flow rate : 0.800 ml/min

No.	Rt	Area	Area(%)	Height
1	9.083	7547631	93.1	467369
2	10.317	556773.4	6.9	30464
		8104404	100	497833

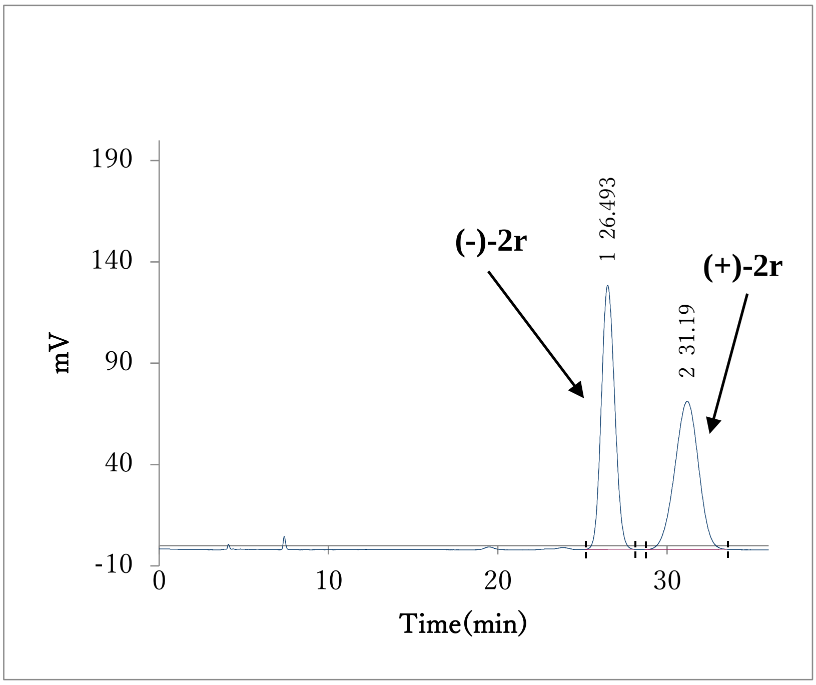

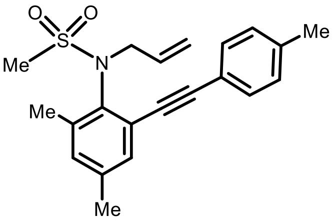

rac-**2r**
AS-H
eluent : 5% IPA in hexane
flow rate : 0.800 ml/min

No.	Rt	Area	Area(%)	Height
1	26.493	7173448	50.1	130271
2	31.19	7146590	49.9	73200
		14320038	100	203471

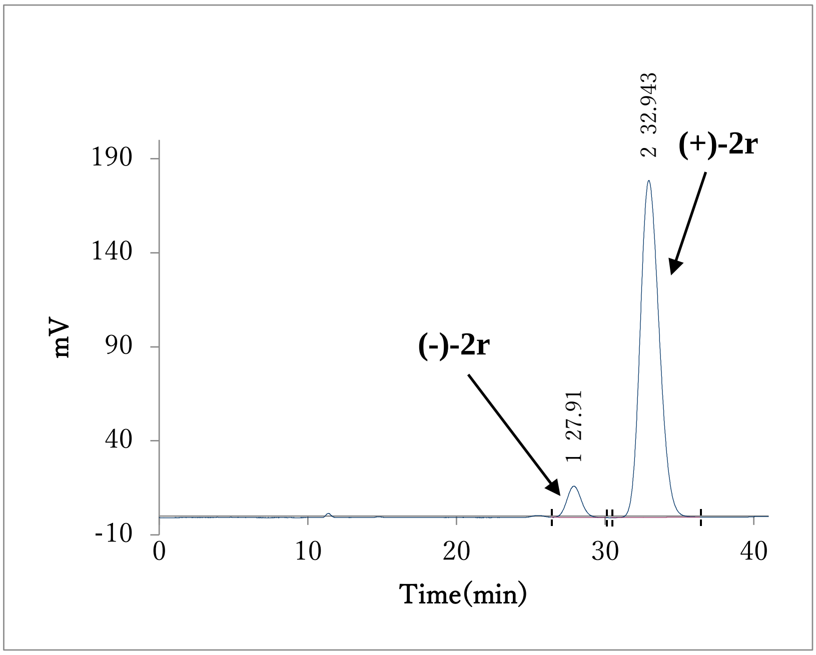

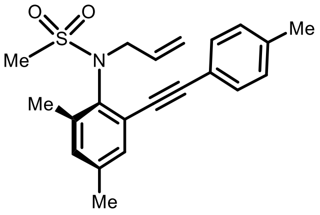

2r
AS-H
eluent : 5% IPA in hexane
flow rate : 0.800 ml/min

No.	Rt	Area	Area(%)	Height
1	27.91	1123997	6.6	16653
2	32.943	15902586	93.4	179203
		17026584	100	195856

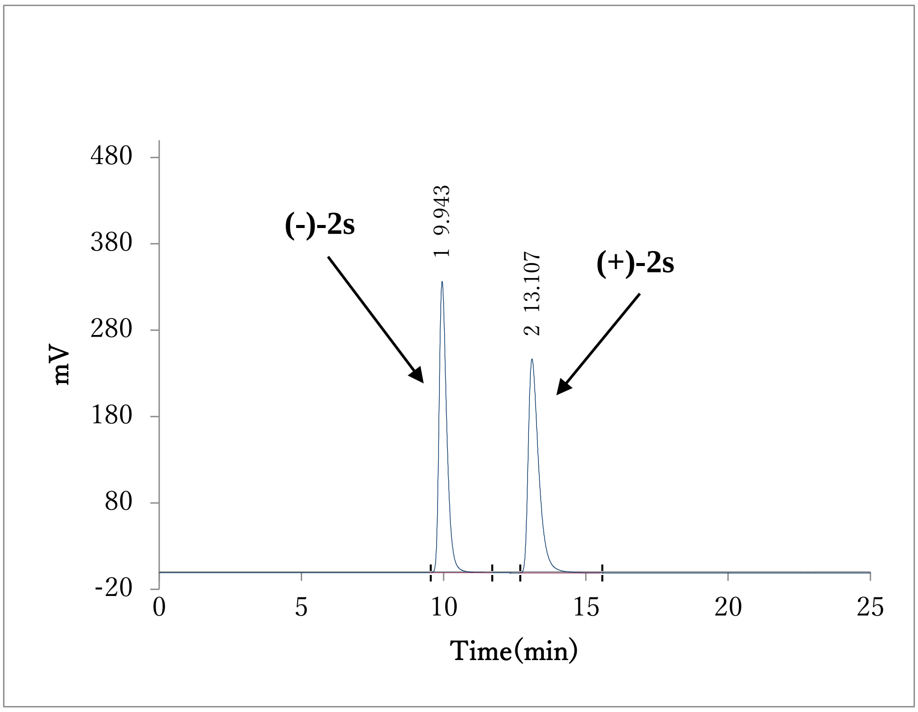

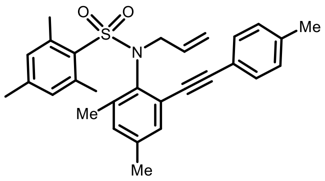

rac-**2s**

OD-3
eluent : 1% IPA in hexane
flow rate : 0.800 ml/min

No.	Rt	Area	Area(%)	Height
1	9.943	6343040	49.8	337423
2	13.107	6385348	50.2	247913
		12728388	100	585336

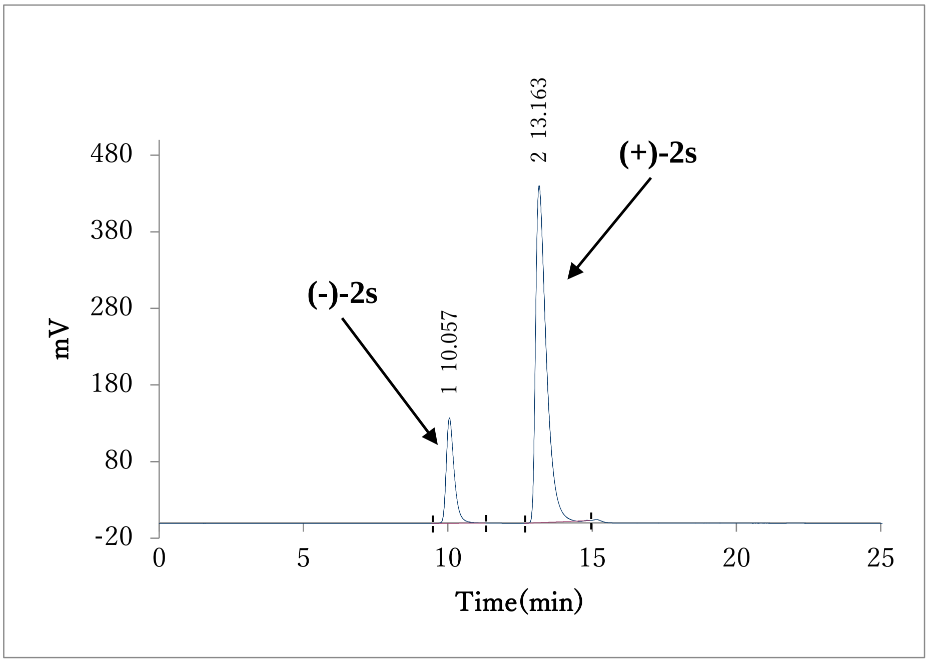

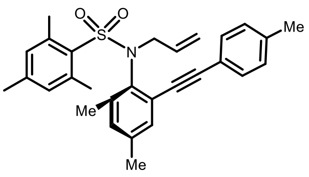

2s

OD-3
eluent : 1% IPA in hexane
flow rate : 0.800 ml/min

No.	Rt	Area	Area(%)	Height
1	10.057	2618016	18.7	137592
2	13.163	11401047	81.3	440314
		14019063	100	577906

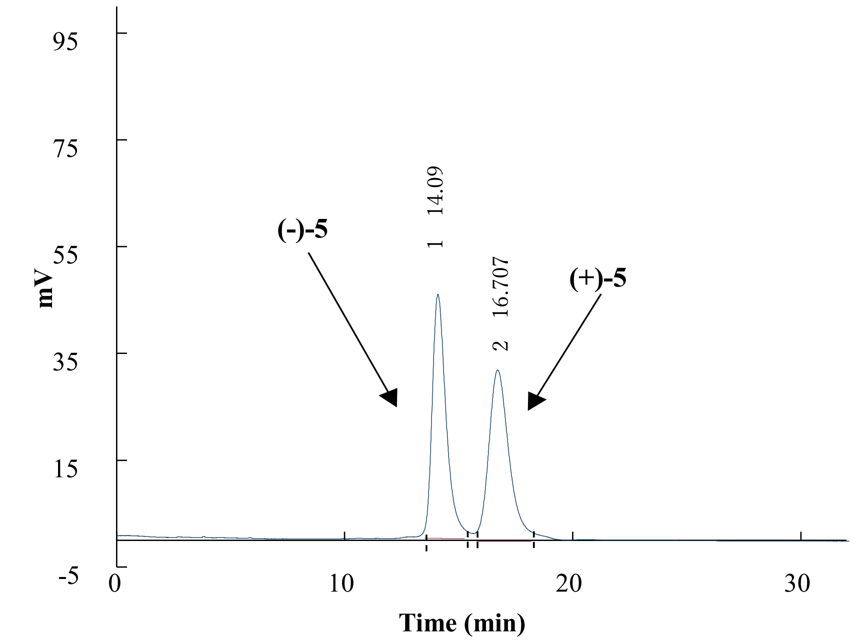

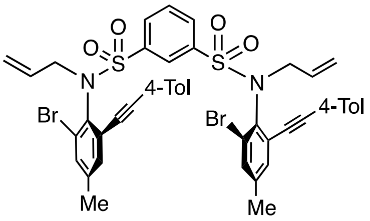

chiral-**5** (racemate)

AD-H
eluent: 10% IPA in hexane
flow rate: 0.80 min

No.	Rt	Area	Area(%)	Height
1	14.09	1749424	50.4	43960
2	16.707	1724361	49.6	30351
		3473784	100	74311

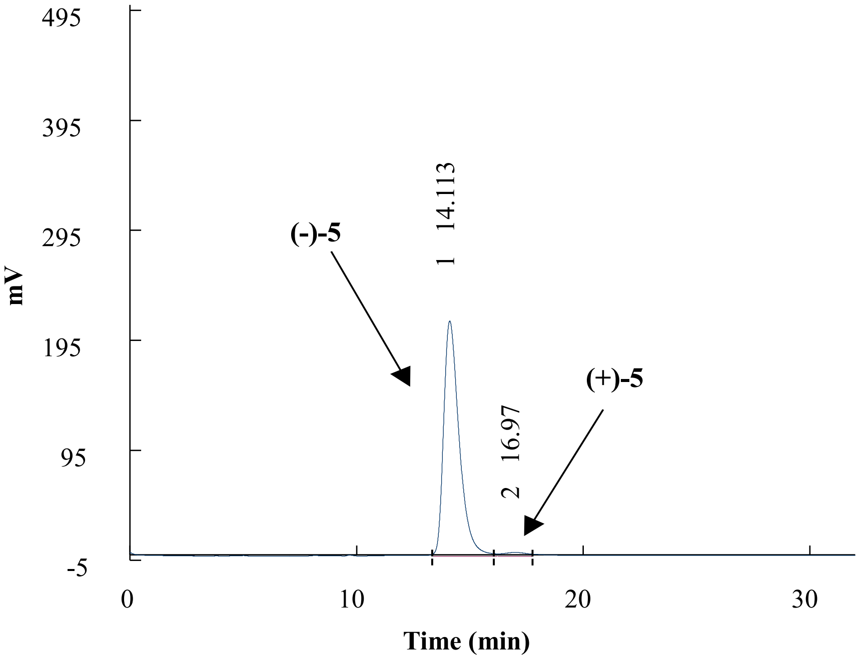

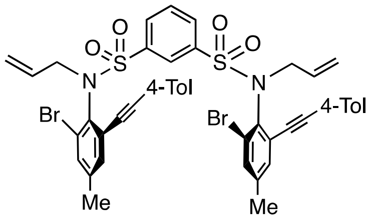

chiral-**5** (99% ee)

AD-H
eluent: 10% IPA in hexane
flow rate: 0.80 min

No.	Rt	Area	Area(%)	Height
1	14.113	9878409	99.3	211717
2	16.97	67729.6	0.7	1424
		9946139	100	213141

X-Ray crystal data of **2o** (CheckCIF/PLATON report)

checkCIF/PLATON report

Structure factors have been supplied for datablock(s) hk210406a_a

THIS REPORT IS FOR GUIDANCE ONLY. IF USED AS PART OF A REVIEW PROCEDURE FOR PUBLICATION, IT SHOULD NOT REPLACE THE EXPERTISE OF AN EXPERIENCED CRYSTALLOGRAPHIC REFEREE.

No syntax errors found. CIF dictionary Interpreting this report

Datablock: hk210406a_a

Bond precision:	C-C = 0.0070 A		Wavelength=0.71073
Cell:	a=10.3010(16)	b=9.0527(14)	c=25.570(4)
	alpha=90	beta=99.171(5)	gamma=90
Temperature:	100 K		

	Calculated	Reported
Volume	2354.0(6)	2354.0(6)
Space group	P 21	P 21
Hall group	P 2yb	P 2yb
Moiety formula	C26 H24 N2 O4 S	C26 H24 N2 O4 S
Sum formula	C26 H24 N2 O4 S	C26 H24 N2 O4 S
Mr	460.53	460.53
Dx,g cm-3	1.299	1.299
Z	4	4
Mu (mm-1)	0.173	0.173
F000	968.0	968.0
F000’	968.92	
h,k,lmax	12,10,30	12,10,30
Nref	8327[4452]	8236
Tmin,Tmax	0.946,0.986	0.830,0.990
Tmin’	0.936	

Correction method= # Reported T Limits: Tmin=0.830 Tmax=0.990
AbsCorr = MULTI-SCAN

Data completeness= 1.85/0.99 Theta(max)= 25.027

R(reflections)= 0.0511(6426) wR2(reflections)= 0.1191(8236)

S = 1.040 Npar= 601

The following ALERTS were generated. Each ALERT has the format

test-name_ALERT_alert-type_alert-level.

Click on the hyperlinks for more details of the test.

Alert level C

PLAT089_ALERT_3_C Poor Data / Parameter Ratio (Zmax < 18) .... 7.38 Note

PLAT220_ALERT_2_C NonSolvent Resd 2 C Ueq(max)/Ueq(min) Range 3.4 Ratio

PLAT340_ALERT_3_C Low Bond Precision on C-C Bonds .... 0.00698 Ang.

PLAT911_ALERT_3_C Missing FCF Refl Between Thmin & STh/L= 0.595 13 Report

---

Alert level G

PLAT371_ALERT_2_G Long C(sp2)-C(sp1) Bond C6 - C7 . 1.44 Ang.

PLAT371_ALERT_2_G Long C(sp2)-C(sp1) Bond C8 - C9 . 1.43 Ang.

PLAT371_ALERT_2_G Long C(sp2)-C(sp1) Bond C32 - C33 . 1.44 Ang.

PLAT371_ALERT_2_G Long C(sp2)-C(sp1) Bond C34 - C35 . 1.45 Ang.

PLAT883_ALERT_1_G No Info/Value for _atom_sites_solution_primary . Please Do !

PLAT909_ALERT_3_G Percentage of I>2sig(I) Data at Theta(Max) Still 47% Note

PLAT910_ALERT_3_G Missing # of FCF Reflection(s) Below Theta(Min). 2 Note

PLAT933_ALERT_2_G Number of HKL-OMIT Records in Embedded .res File 1 Note

PLAT978_ALERT_2_G Number C-C Bonds with Positive Residual Density. 0 Info

PLAT992_ALERT_5_G Repd & Actual _reflns_number_gt Values Differ by 3 Check

---

0 **ALERT level A** = Most likely a serious problem - resolve or explain

0 **ALERT level B** = A potentially serious problem, consider carefully

4 **ALERT level C** = Check. Ensure it is not caused by an omission or oversight

10 **ALERT level G** = General information/check it is not something unexpected

1 ALERT type 1 CIF construction/syntax error, inconsistent or missing data

7 ALERT type 2 Indicator that the structure model may be wrong or deficient

```
5 ALERT type 3 Indicator that the structure quality may be low
0 ALERT type 4 Improvement, methodology, query or suggestion
1 ALERT type 5 Informative message, check
```

It is advisable to attempt to resolve as many as possible of the alerts in all categories. Often the minor alerts point to easily fixed oversights, errors and omissions in your CIF or refinement strategy, so attention to these fine details can be worthwhile. In order to resolve some of the more serious problems it may be necessary to carry out additional measurements or structure refinements. However, the purpose of your study may justify the reported deviations and the more serious of these should normally be commented upon in the discussion or experimental section of a paper or in the "special_details" fields of the CIF. checkCIF was carefully designed to identify outliers and unusual parameters, but every test has its limitations and alerts that are not important in a particular case may appear. Conversely, the absence of alerts does not guarantee there are no aspects of the results needing attention. It is up to the individual to critically assess their own results and, if necessary, seek expert advice.

Publication of your CIF in IUCr journals

A basic structural check has been run on your CIF. These basic checks will be run on all CIFs submitted for publication in IUCr journals (*Acta Crystallographica*, *Journal of Applied Crystallography*, *Journal of Synchrotron Radiation*); however, if you intend to submit to *Acta Crystallographica Section C* or *E* or *IUCrData*, you should make sure that full publication checks are run on the final version of your CIF prior to submission.

Publication of your CIF in other journals

Please refer to the *Notes for Authors* of the relevant journal for any special instructions relating to CIF submission.

PLATON version of 12/09/2022; check.def file version of 09/08/2022

Datablock hk210406a_a - ellipsoid plot

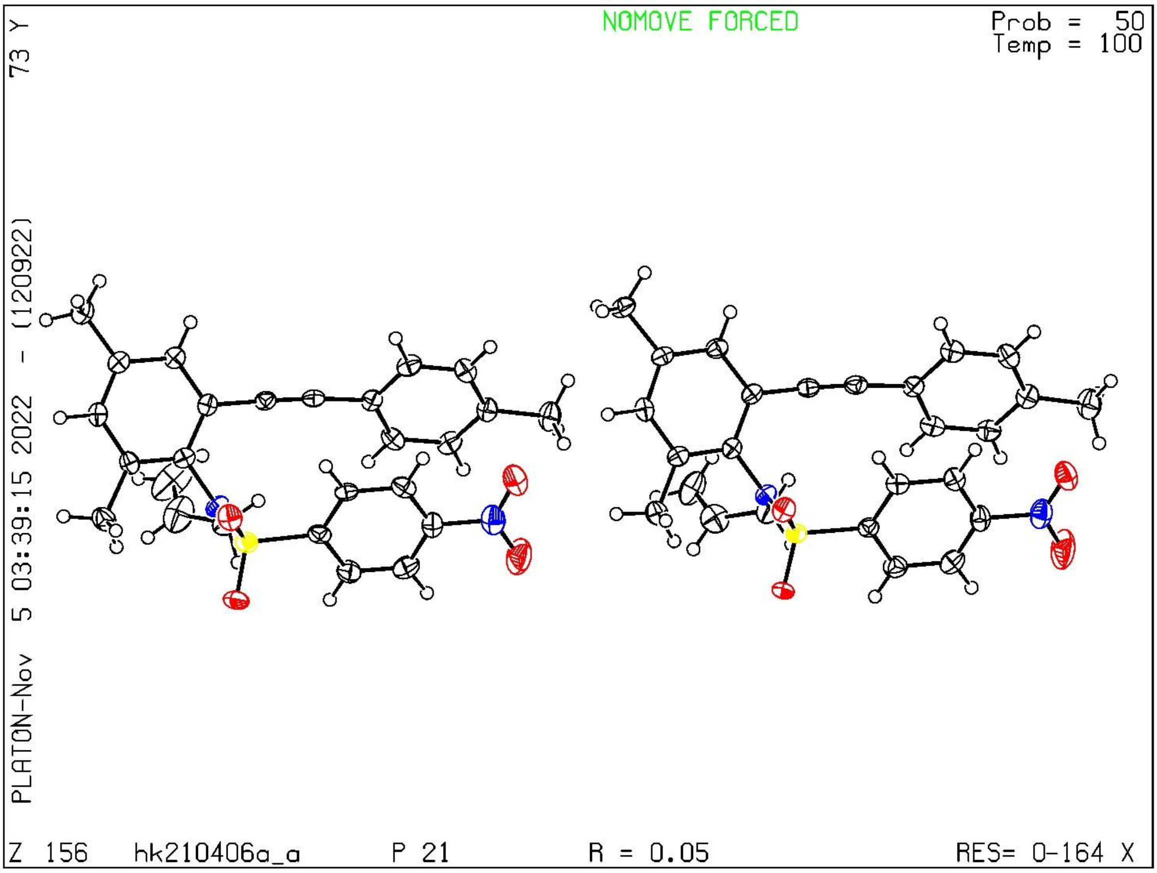

Evaluation of rotational barrier of **2i**, **2r**

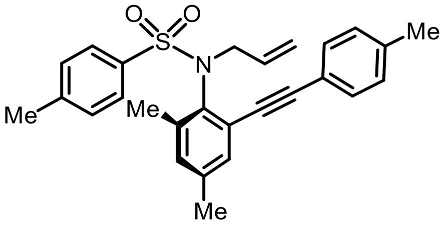

2i

Δ$G^‡$ = 28.7 kcal/mol

60 °C	
time (h)	ee (%)
0	87
3	85
6	83
12	79
30	69
42	63

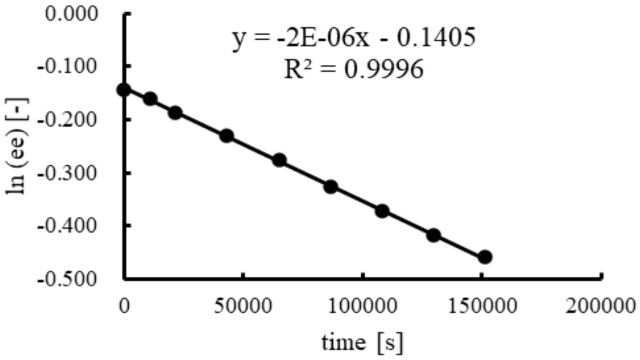

temp (°C)	k (s^{-1})	Δ$G^‡$ (kcal/mol)	$t_{1/2}$ (days)
60	1.06×10^{-6}	28.7	7.6

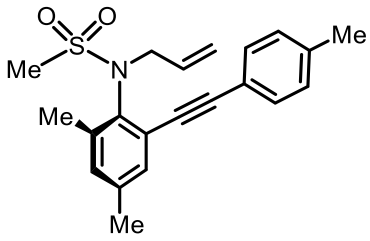

2r

Δ$G^‡$ = 28.3 kcal/mol

60 °C	
time (h)	ee (%)
0	87
3	84
6	80
12	74
30	58
33	57

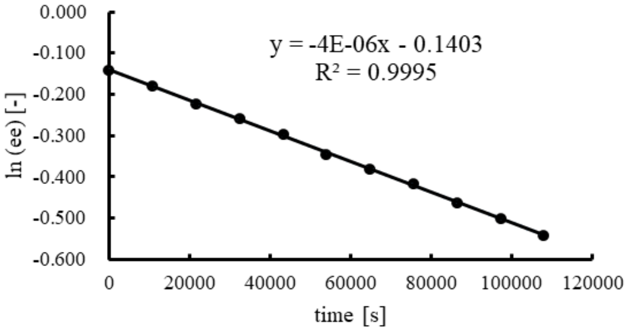

temp (°C)	k (s^{-1})	Δ$G^‡$ (kcal/mol)	$t_{1/2}$ (days)
60	1.89×10^{-6}	28.3	4.3
